# Factors associated with length of stay in care homes: a systematic review of international literature

**DOI:** 10.1186/s13643-019-0973-0

**Published:** 2019-02-20

**Authors:** Danni Collingridge Moore, Thomas J. Keegan, Lesley Dunleavy, Katherine Froggatt

**Affiliations:** International Observatory on End of Life Care, Lancaster University, Lancaster, UK

**Keywords:** Systematic review, Long-term care facility, Care home, Nursing home, Length of stay

## Abstract

**Background:**

A number of studies have explored factors associated with resident length of stay in care homes; however the findings of these studies have not been synthesized. The aim of this paper is to provide a systematic review of factors associated with length of stay until death and the strength of evidence supporting each of these factors.

**Methodology:**

This is a systematic review; databases included MEDLINE, EMBASE, PsycINFO, CINAHL, Proquest, the Cochrane Library and Web of Science were searched. Observational studies, either prospective or retrospective, that explored multiple factors associated with length of stay until death in care homes were included. Studies that met the inclusion criteria were sourced, data extracted and assessed for quality. Data synthesis combined the direction and significance of association with the quality of the study, resulting in strong, moderate, weak or inconclusive evidence for each factor identified.

**Results:**

Forty-seven studies were identified as meeting the inclusion criteria. After quality assessment, 14 studies were judged to be of a high quality, 31 of a moderate quality and 2 of a low quality. Three factors had strong evidence to support their association with shorter lengths of stay: shortness of breath, receipt of oxygen therapy and admission to a facility providing nursing care.

**Conclusions:**

This review summarized the factors associated with length of stay. It found stronger evidence for physical functioning being associated with shorter lengths of stay than for cognitive functioning. An understanding of expected length of stay for older adults admitted to a care home is important for estimating lifetime costs and the implications of reforming funding arrangements for social care. Further research is needed to explore heterogeneity in this area.

**Electronic supplementary material:**

The online version of this article (10.1186/s13643-019-0973-0) contains supplementary material, which is available to authorized users.

## Background

The global population is ageing; 35% of the European population and 28% of the North American population are expected to be aged 60 years or over by 2050 [1]. By 2030, the number of older persons in the world is estimated to increase to 1.4 billion, resulting in 2.1 billion in 2050 and potentially 3.1 billion in 2100 [2]. As a consequence of this growth, deaths in this population group will also increase; among those 80 years and older, deaths are projected to rise to over 15 million by 2030 [3]. Providing care for an ageing, and dying, population is, and will continue to be, a novel challenge for healthcare systems around the world.

Older adults are more likely to be frail, have multiple comorbidities and suffer from chronic diseases, including dementia, than younger adults. As end of life approaches, common preference among older adults is to remain in the home until death [4]; however, this may not be possible for those requiring high levels of care or without access to formal or informal care providers. The majority of deaths in older adults with dementia occur in long-term care facilities [5, 6]; in England and Wales, it is estimated that by 2040, care homes will become the most common place of death [7]. Although terminology and typology vary between countries, a care home or long-term care facility generally refers to a collective institutional setting where care is provided to older adults, who live there, 24 hours a day, 7 days a week [8]. The provision of care homes, in terms of type (with or without nursing), number of beds and staffing levels, provision of funding and relationship with the wider health care system, also varies between countries [8]. This paper will use the term care home throughout.

Transitions from living in the community to a care home can be varied, and reflective of individual circumstances, such as health status, access to care and financial circumstances. A systematic review by Luppa et al. identified characteristics associated with admission into care homes from 36 prospective observational studies of population samples, which followed older adults in the community to care home admission [9]. Older adults who enter care homes are more likely to be older, have lower self-rated health, functional impairments, cognitive impairments and dementia [9]. In some cases, admission may follow a long period of physical or cognitive decline leaving caregivers unable to provide the level of care required by the resident. In other cases, a trigger event, such as a stroke or fall, may lead to a resident being unable to return to living independently in their own home.

Compared to older adults residing in the community, care home residents have poorer health, including higher rates of dementia, stroke and severe mental illness [10]. An increased use of health services is common; care home residents also have high rates of hospital and emergency department admission, primary care contact and use of out of hours services [11–13]. In 2014, Barclay et al. conducted a prospective study following residents in six residential care homes until death and identified four trajectories towards end of life: anticipated dying, uncertain dying, unexpected death and unpredicted dying [14]. Briefly, the trajectories were based on whether the resident’s death was expected and the presence of a sudden illness or an acute event.

Despite these high healthcare needs, the availability of data on care homes and their residents varies internationally. Some countries have minimum datasets, such as Minimum Data Set in the USA [15], which provide a wealth of routinely collected data for potential research. Data on care home residents may also be available in larger cohort studies, such as the English Longitudinal Study of Ageing (ELSA) [16] or the Survey of Health, Ageing and Retirement in Europe (SHARE) [17]. However, care home residents are frequently excluded from datasets focusing on older adults; in the UK few observational studies either include care home residents at baseline or follow up community-dwelling residents into care homes [18]. Methodological challenges of conducting research with older adults in care homes have been described elsewhere, including recruitment barriers, difficulties engaging with staff and adapting to competing demands on time and resources [19].

Length of stay from admission until death is a simple measure that could inform our understanding of care home residents and identify variation in health service use. Length of stay is often reported as an outcome in care home research; however, there is little consensus on the factors associated with length of stay, or how length of stay varies among residents, nationally and internationally. Building on the European Association for Palliative Care Taskforce on Palliative Care in Long-term Care Settings for Older People [20], Froggatt et al. conducted a survey of palliative care provision in long-term care facilities in 29 European countries [21]. Data on average length of stay was returned by 14 countries (48%) and ranged from 63 days (Israel) to over 2000 days (Luxembourg). Using an average to report length of stay can be misleading; a minority of residents residing in care homes for an extended period, sometimes over 10 years, can skew an average measure, eclipsing residents admitted for very short periods of time.

An understanding of length of stay data has several potential benefits: it can inform service planning to accommodate a growing number of residents and, combined with other measures, be used to inform the provision of care within the wider health system. It can be used to identify variation across care homes, highlighting facilities with lengths of stay either below or above the expected based on the resident profile. Residents, their relatives and healthcare professionals could also benefit from this information to inform decision-making regarding relocation into long-term care, and as a guide to support the provision and delivery of palliative care. Unlike mortality prediction tools, which have been developed to aid the identification of residents likely to die within a specified time frame [22–24], an understanding of length of stay within the care home population provides an overview of how care homes are being utilized by the older adults.

One previously conducted systematic review on length of stay in care home residents, conducted in 2013, identified five studies conducted in nursing homes; however, the review was limited to short-term mortality on health-related characteristics [25]. The aim of this paper is to systematically review the factors identified in observational studies as associated with length of stay in care homes.

## Methodology

### Identification of papers

A protocol for the systematic review was prepared prior to conducting the review. A systematic search strategy was developed and reported using criteria established in the Preferred Reporting Items for Systematic Review and Meta-Analysis Protocols (PRISMA-P) 2015 statement [26]. The search strategy included a combination of free-text terms and subject indexing terms, such as MeSH and EMTREE (Table 1). The search strategy was developed through identification of key terms in the titles and abstracts of relevant studies identified in an initial scoping search of the literature.

**Table 1:** Example search strategy

Ovid MEDLINE 1. exp. Nursing Homes/ 2. exp. Homes for the Aged/ 3. “care home*”.ti. 4. “nursing home*”.ti. 5. “nursing facilit*”.ti. 6. “residential home*”.ti. 7. “residential care”.ti. 8. “residential long term care”.ti. 9. “institutionali?ed”.ti. 10. “institutional* residen*”.ti. 11. “institutional* care*”.ti. 12. (“long term” adj1 “care facilit*”).ti. 13. (“long term” adj1 “care residen*”).ti. 14. (“long term” adj1 “care institution*”).ti. 15. (“long term” adj1 “institution* care*”).ti. 16. (“institution*” adj1 “long term care*”).ti. 17. “survival”.ti,ab. 18. “mortality”.ti,ab. 19. “death”.ti,ab. 20. “length” adj1 “stay”.ti,ab. 21. “life” adj1 “expectanc*”.ti,ab. 22. 1 or 2 or 3 or 4 or 5 or 6 or 7 or 8 or 9 or 10 or 11 or 12 or 13 or 14 or 15 or 16 23. 17 or 18 or 19 or 20 or 21 24. 22 and 23	

The following electronic databases were searched for articles published in peer-reviewed journals, from database inception to September 2016, and were not limited by language or publication restrictions: MEDLINE, EMBASE, PsycINFO, Cumulative Index to Nursing and Allied Health Literature (CINAHL), Proquest, the Cochrane Library, including the Cochrane Methodology Register, Cochrane Central Register of Controlled Trials (CENTRAL), Cochrane Database of Systematic Reviews (CDSR), Database of Abstracts of Reviews of Effect (DARE), Health Technology Assessment (HTA) database and NHS Economic Evaluation Database (NHS EED), Web of Science, the Campbell Library, SCOPUS and Social Care Online.

In cases where a conference abstract or unpublished research met the inclusion criteria, the lead author was contacted where possible. Additional papers were identified through other sources, including reviewing the reference lists of publications that met the inclusion criteria, reverse citation searches and grey literature. Reverse citation searches were undertaken on included papers using the ISI Web of Science Citation Databases. Grey literature was searched using OpenGrey. Websites were searched using an abbreviated search strategy; these included the World Health Organization, European Association of Palliative Care and Age UK.

### Inclusion and exclusion criteria

The review focused on older adults, defined as any adult or groups of adults aged 65 years or above. Participants were included in the review if they resided in a care home or long-term care facility. As previously stated, the term “long-term care facility” generally refers to a collective institutional setting where care is provided to older people, who live there, 24 hours a day, 7 days a week, as defined previously [8]. In the review, this definition was also applied to care homes. Studies in hospitals, assisted living facilities, sheltered housing and hospices were excluded.

The review had one outcome measure, length of stay, defined as length of stay within a care home until death. Length of stay could be measured in days, months, years or any other unit of time; and measured from any time point after admission. Studies exploring length of stay until discharge were excluded. The review was restricted to observational studies, including retrospective or prospective cohort studies and case-control studies.

In the first stage of screening, a decision on whether a paper met the inclusion criteria was based on the study title and abstract. In the second stage of screening, a final decision on inclusion was made based on reading the full paper.

**Fig. 1:**
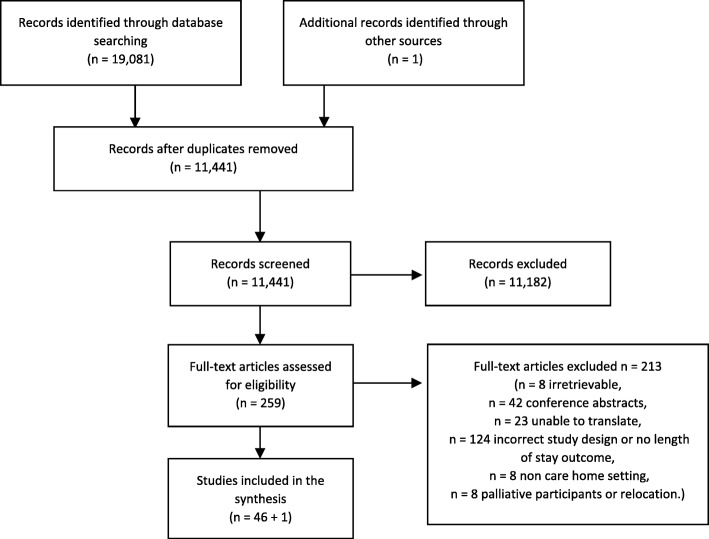
PRISMA flowchart

In both stages, screening was conducted by one reviewer (DCM), and decisions checked by a blinded second reviewer (LD) on a subsample of 10% of the papers. Discrepancies were discussed, and a final decision was made by a third member of the research team (KF). Reasons for excluding full papers were recorded and reported (Fig. 1).

### Data extraction

Data from the included studies were extracted using a data extraction form specifically developed for this review, informed by STROBE statement on the reporting of observational studies [27]. Data was extracted by one reviewer (DCM). The data collected from each study included: information on participants (number of residents, age of residents, gender of residents), the care home (type of care home, number of care homes), the methodology (study setting, type of study, dataset used, data collection period, follow up period, how length of stay was defined, variables included in model, statistical method used) and information on missing data. The results of the paper, including risk measure used and its value, confidence intervals and measure of significance were also extracted.

If a study contained more than one cohort, both cohorts were included as separate groups, such as Engle and Graney [28]. In development and validation studies, data from the development cohort was extracted. In studies where men and women were listed separately, such as Hedinger, Hamming and Bopp, [29], the cohort that reflected the sample majority was used. In cases where the results were significant, either through significance testing or confidence intervals, the result was classed as significant. In cases were no data on an included variable was reported in the results, the result was not significant or the confidence interval indicated none significance, the result was classed as not significant. If a study reported more than one result for the same outcome, i.e. age reported in groups, the result with either the most significant or the largest ratio was used to avoid duplication. Factors included in less than three studies were excluded from the analysis to avoid bias in the evidence synthesis, which required a minimum of three studies to apply the data synthesis.

### Quality assessment

There are numerous tools to assess the methodological quality and risk of bias in randomized controlled trials; however, few are designed specifically for observational studies [30]. To ensure that the quality assessment tool was appropriate for the topic area, the review used a modified version of the quality assessment tool used by Luppa et al., adapted for application to studies on factors associated with length of stay in care homes. The adapted tool has 14 items, scored as either 0 (not meeting the criteria) or 1 (meeting the criteria) (see Additional file 1). A score of 75% or more of the assessment criteria was defined as high-quality studies, between 50% and 75% was defined as moderate quality and less than 50% was defined as low quality.

### Data synthesis

Due to heterogeneity in the setting, sample and tools used, a meta-analysis was not appropriate to synthesize the data. Data synthesis was split into two stages. Firstly, the characteristics of the study were described, including the study design, sample size and variables reported. Numerical data was tabulated and presented for each factor, showing effect size and direction.

Secondly, in a similar approach used by Luppa et al., all factors identified across the studies were pooled and grouped, alongside the direction of the variables effect on length of stay and their significance (positive, negative and not significant). These findings were then cross-referenced with the quality assessment score for the study (see Additional files 2 and 3).

A judgment was made on the strength of the evidence for each risk factor from this cross tabulation. A factor was classed as being supported by strong evidence if there are consistent findings in at least 75% of studies in at least three high-quality studies. Moderate evidence was classed as consistent findings in at least 50% of studies in at least two high-quality studies. Weak evidence was classed as findings of one high-quality study and of at least two moderate- to low-quality studies or consistent findings (≥ 75%) in at least four or more moderate- to low-quality studies. If a risk factor was not classed as having strong, moderate or weak evidence, it was classed as inconclusive.

## Results

The searches of the electronic databases identified 19,081 titles and abstracts, resulting in 11,441 after deduplication. Based on applying the inclusion criteria to the abstract of the paper, 11,182 abstracts were excluded, identifying 259 abstracts for which full papers were sourced. Eight papers could not be sourced, 42 were excluded as they were conference proceedings, 124 were excluded based on study design or did not have a risk measure, 23 were in a language which could not be translated by the research team, 8 were not conducted in care homes and 8 were exclusively in patients approaching end of life or concerned relocation. One paper was identified through other sources.

In total, 47 studies met the inclusion criteria for the review (Additional file 4). Study designs as reported by the papers included prospective studies (16), retrospective studies (13), longitudinal, cohort or follow-up studies (8), secondary analysis, linked observational or population-based studies (4) and case-control studies (2). Four papers did not report study design.

Seven of the 47 papers were split into more than 1 cohort, mostly through reporting follow-up for more than 1 time point; each cohort was included in the synthesis separately resulting in 57 included cohorts from 47 papers. One year follow-up was based on 26 cohorts.

Eighteen studies were conducted in the USA, 10 in Europe, 7 in the UK, 4 in Hong Kong, 2 in New Zealand and 5 elsewhere. Total sample size was 942,626, sample sizes ranged from 49 to 218,088, with 9 studies including residents with dementia, Alzheimer’s disease or Parkinson’s disease only. The majority of studies included residents aged over 65 years who were newly admitted to the facility. Average age ranged from 76.03 (10.08) to 92.9 (3.0), and the percentage of the sample that was female ranged from 59.6 to 89.7%, where reported. In terms of how the study described the facility in which data were collected, 32 studies were based in nursing homes, 6 in long-term care facilities and 9 in residential homes, care homes or other. Length of follow-up ranged from 1 month to 11 years, with three studies collecting data retrospectively or until death (see Additional file 2).

### Methodological quality

Fourteen of the 47 studies were judged to be of a high quality, 31 of a moderate quality and 2 of a low quality. The lowest scores on the methodological quality were on reporting the training and quality control methods for interviewers’ technique (reported in 8/47 papers) and the reliability and/or validity of study instruments (reported in 8/47 papers).

### Factors associated with length of stay

Factors associated with length of stay, minimum and maximum risk results and strength of evidence have been summarized in Table 2. Three factors had strong evidence to support their association with shorter lengths of stay: shortness of breath, receipt of oxygen therapy and admission to a nursing home. Nine factors had moderate evidence to support their association with shorter lengths of stay: cancer, increased contact with primary care, poor general health, poor mobility, low BMI or malnutrition, poor physical functioning, presence of pressure ulcers, older age and being male.

**Table 2:** Factors with evidence to support a relation to length of stay

Predictors	Lowest ratio		Highest ratio		Strong evidence	Moderate evidence	Weak evidence
Care home characteristics—nursing	1.14 (1.01–1.30)	HR (CI)	1.85 (1.50–2.23)	OR (CI)	X		
Clinical intervention—oxygen therapy	1.6 (1.4–1.8)	HR (CI)	2.61 (1.30–5.21)	OR (CI)	X		
Shortness of breath	1.5 (1.3–1.9)	HR (CI)	4.88	OR	X		
Age	0.70 (0.53–0.93)	OR (CI)	3.25 (2.39–4.41)	HR (CI)		X	
Cancer	1.36 (1.21–1.53)	HR (CI)	374 (174–804)	OR (CI)		X	
Contact with primary care—number of contacts	1.65 (1.43–1.92)	HR (CI)	1.90 (1.2–3.2)	HR (CI)		X	
Gender—being female^a^	0.49 (0.36–0.66)	HR (CI)	2.10 (1.22–3.60)	RR		X	
General health	0.609 (0.416–0.891)	HR (CI)	16.18 (11.41–22.95)	HR (CI)		X	
Mobility	0.93 (0.84–1.02)	RR (CI)	4.6 (2.3–12.7)	OR (CI)		X	
Nutrition—low BMI or malnutrition	0.81 (0.57–1.16)	HR (CI)	2.26 (1.56–3.28)	RR (CI)		X	
Physical functioning	0.23 (0.10–0.50)	HR (CI)	8.0 (2.2–47.8)	OR (CI)		X	
Pressure ulcers	1.03 (1.00–1.06)	HR (CI)	2.7 (1.37–5.1)	OR (CI)		X	
Admission source—hospital	0.81 (0.43–1.52)	HR (CI)	2.02 (1.2–3.3)	HR (CI)			X
Behaviour problems	0.90 (0.78–1.05)	RR (CI)	3.95	OR			X
Biochemical indicators	0.19 (0.10–0.36)	HR (CI)	3.207 (1.023–0.060)	OR (CI)			X
Cognitive function	0.8	OR	10.5 (1.02–1.08)	HR (CI)			X
Dementia or Alzheimer’s disease	0.48 (0.52–1.05)	OR (CI)	1.96 (1.86–2.06)	IRR (CI)			X
Depression	0.91 (0.82–1.01)	RR (CI)	1.26 (1.00–1.58)	HR (CI)			X
Diabetes	0.99 (0.88–1.12)	HR (CI)	3.789 (1.266–1.336)	OR (CI)			X
Ethnicity—white^a^	0.69 (0.57–0.85)	RR (CI)	0.89 (0.76–1.06)	RR (CI)			X
Falls and fractures^a^	0.40 (0.21–0.74)	OR (CI)	1.2	OR			X
Feeding—appetite	1.39 (1.37–1.41)	OR (CI)	2.16 (1.59–2.93)	HR (CI)			X
Feeding—feeding tube or help with feeding or diet	0.53 (0.31–0.90)	HR (CI)	4.05 (1.40–1.73)	HR (CI)			X
Hallucinations, delusions, wandering or delirium	0.74 (0.77–1.15)	RR (CI)	2.97 (1.50–5.88)	RR			X
Incontinence or catheter use	0.93	RR	3.2 (1.46–7.2)	OR (CI)			X
Marital status—not married ^a^	0.90 (0.78–1.05)	RR (CI)	1.31 (1.09–1.59)	RR (CI)			X
Respiratory disorders/COPD	1.17 (1.04–1.33)	HR (CI)	3.4 (1.3–8.8)	OR (CI)			X
Stroke	0.79 (0.33–0.70)	OR (CI)	1.79 (1.68–1.90)	IRR (CI)			X
Vaccinations^a^	0.439 (0.208–0.924)	HR (CI)	0.47 (0.28–0.78)	OR (CI)			X
Vision impairment	0.94 (0.84–1.05)	RR (CI)	1.38 (1.20–1.57)	RR (CI)			X

Combined results of evidence rating for each factor identified: strong, moderate, weak and inconclusive evidence

^a^Associated with longer length of stay

Weak evidence to support their association with shorter lengths of stay was identified for admission from hospital, behaviour problems, biochemical indicators, poor cognitive function, dementia or Alzheimer’s disease, depression, diabetes, poor appetite, presence of a feeding tube, help with feeding or diet, hallucinations, delusions, wandering or delirium, incontinence or catheter use, respiratory disorders or COPD, history of stroke, vision impairment, and being married. History of fractures or falls, being of white ethnicity and vaccinations decreased the risk of shorter lengths of stay.

### Subgroup analysis—studies with follow-up periods of 1 year or less

A subgroup analysis was conducted on studies that investigated a follow-up period of 1 year or less (Table 3). Twenty-six papers were included, with the same criteria as applied to the full sample. Within 1 year of follow-up, oxygen therapy remained strongly associated with shorter stays, although residence in a nursing home and shortness of breath were associated with moderate evidence. Increased age, cancer, poor appetite, being male, poor general health, low BMI or malnutrition, poor physical functioning remained supported by moderate evidence, respiratory disorders or COPD increased from weak to moderate evidence.

**Table 3:** Factors with evidence to support a relation to length of stay up to 1 year

Predictors	Lowest ratio		Highest ratio		Strong evidence	Moderate evidence	Weak evidence
Clinical intervention—oxygen therapy	1.6 (1.4–1.8)	HR (CI)	2.61 (1.30–5.21)	OR (CI)	X		
Age	1.02 (0.98–1.07)	HR (CI)	3.05	HR		X	
Cancer	1.36 (1.21–1.53)	HR (CI)	374 (174–804)	OR (CI)		X	
Care home characteristics—nursing	1.14 (1.01–1.30)	HR (CI)	1.48 (1.36–1.61)	HR (CI)		X	
Feeding—appetite	1.39 (1.37–1.41)	OR (CI)	2.16 (1.59–2.93)	HR (CI)		X	
Gender—being female^a^	0.49 (0.36–0.66)	HR (CI)	2.10 (1.22–3.60)	RR		X	
General health	1.1	OR	6.04 (4.19–8.71)	OR (CI)		X	
Nutrition—low BMI or malnutrition	0.844 (0.766–0.930)	HR (CI)	2.26 (1.56–3.28)	RR (CI)		X	
Physical functioning	0.718 (0.644–0.801)	HR (CI)	8.0 (2.2–47.8)	OR (CI)		X	
Respiratory disorders/COPD	1.17 (1.04–1.33)	HR (CI)	3.4 (1.3–8.8)	OR (CI)		X	
Shortness of breath	1.5 (1.3–1.9)	HR (CI)	2.69 (2.20–3.29)	HR (CI)		X	
Admission source—hospital	0.81 (0.43–1.52)	HR (CI)	2.02 (1.2–3.3)	HR (CI)			X
Biochemical indicators	0.25	PCC	1.55 (1.12–2.13)	OR (CI)			X
Cognitive function	0.8	OR	10.5 (1.02–1.08)	HR (CI)			X
Falls and fractures^a^	0.40 (0.21–0.74)	OR (CI)	1.2	OR			X
Feeding—feeding tube or help with feeding or diet	0.7	OR	4.05 (1.40–1.73)	HR (CI)			X
Incontinence or catheter use	0.93	RR	1.1	OR			X
Pressure ulcers	1.03 (1.00–1.06)	HR (CI)	2.7 (1.37–5.1)	OR (CI)			X

Combined results of evidence rating for each factor identified: strong, moderate, weak and inconclusive evidence—limited to studies with 1 year follow-up or less

^a^Associated with longer length of stay

## Discussion

The aim of this study was to identify factors associated with length of stay in care homes; the discussion will focus on factors identified as having strong or moderate evidence and notable exceptions.

Unsurprisingly, shorter lengths of stay were associated with characteristics related to end of life. Shortness of breath is common in dying residents, and oxygen therapy provides symptom relief associated with breathlessness, both of which were supported by strong evidence [31]. Low BMI and malnutrition were supported by moderate evidence, which are also common in residents approaching death [32]. Admission to a facility providing nursing care was associated with shorter lengths of stay compared to a residential-only facility. It is possible that older adults admitted to residential care homes are more able to function independently than those requiring nursing care, and those who do require nursing care subsequently have higher health needs on admission [33].

It is understandable that older adults die sooner after admission; increased age is associated with frailty, multiple comorbidities and greater healthcare needs. Shorter lengths of stay were associated with contact with primary care, which could reflect greater general practitioner involvement, either as resident health deteriorates or through the provision of palliative care [34]. There was inconclusive evidence to support an association between admission to hospital and shorter lengths of stay. One explanation could be that residents with a poorer diagnosis at admission may have advance care planning in place, including choosing not to be admitted to a hospital [35]. It could also reflect variation in the services offered by different types of facilities; facilities with onsite geriatricians and nursing facilities may be better equipped to provide care and avoid hospital admissions compared to residential facilities; however, this cannot be explored using the current data.

There was moderate evidence to suggest that men had shorter lengths of stay in care homes than women. In all studies that reported the gender profile of the sample, there were substantially more women in the samples than men. This finding could reflect a gender imbalance in admission to care homes; women generally live longer than men, and the sample characteristics could reflect widowed women who are unable to live independently after the death of a spouse [36].

Two disease diagnoses were associated with shorter lengths of stay, cancer and, at 1 year follow-up, respiratory disorders or COPD. Functional impairment and characteristics associated with poor functioning, such as poor mobility and pressure ulcers, were identified as having moderate evidence to support them. Poor general health was associated with shorter lengths of stay, indicating that general measures of health and functioning may be more accurate predictors of length of stay than individual diagnoses.

The notable finding of this review is the weak evidence for poor cognitive function and dementia or Alzheimer’s disease being associated with shorter lengths of stay. In Luppa et al.’s review of predictors of care home admission, strong evidence was found to support both the association of cognitive impairment or dementia and poor physical functioning with care home admission [9], however post-admission, this review found neither cognitive impairment nor dementia nor Alzheimer’s disease to be strongly associated with shorter lengths of stay.

One explanation could be related to life expectancy and disease trajectory. Compression of morbidity in ageing populations is an ongoing trend [37], whereby the onset of chronic illness is occurring later in life, for a relatively short time period before death [38]. However, the median survival time for an older adult with dementia from onset to death is 4.1 years (IQR 2.5–7.6) for men and 4.6 years (IQR 2.9–7.0) for women [39]. Survival varies substantially dependent on age of onset, with those diagnosed younger (between 65 and 69 years) potentially living over 10 years [39]. It is possible that residents with dementia are living longer than those with no cognitive impairment post-care home admission.

Another explanation for these findings could be related to characteristics prior to admission, in particular, caregiver burden. Cognitive impairment is a long-term, chronic condition, which reduces one’s ability to live independently. Research suggests that variation in caregiver burden is associated with caregiver characteristics rather than patient characteristics [40], stress among caregivers of those with dementia has been found to be higher than for caregivers caring for older adults without dementia [41]. Although individual experiences vary, caregivers to those with dementia provide more hours per week spent on caregiving tasks and support a higher number of activities of daily living, as well as being affected by negative consequences of caregiving, such as employment complications, caregiver strain and mental and physical health problems [42]. Residents with physical impairment may be surviving in the community longer than those with cognitive impairment due to lower caregiver burden, and are subsequently admitted to care homes later, leading to shorter lengths of stay.

Finally, the availability of formal home care services may explain this finding. Formal home care services provide support for older adults to remain living in their own homes, undertaking domestic and personal care based on individual needs. It is possible that older adults with functional impairments are more able to locate and access services to support their remaining in the community than those with cognitive impairment, further delaying care home admission.

### Strengths and limitations

To the authors’ knowledge, this is the first systematic review of international literature on factors associated with length of stay in care homes. The review had broad inclusion criteria and was not limited to one type of care home. Efforts to ensure that the studies identified were comparable limited the review to study designs that followed residents until death, meaning that residents who were discharged or moved to another care home were not included. The inclusion of variables related to resident and care home characteristics adds an additional dimension to the review.

The review is potentially limited by the heterogeneity in the terminology used in studies in care homes. Efforts were made to include a multitude of terms related to care homes in the search strategy used to identify studies from a variety of settings; however, it is possible that some studies that included less common terms for care homes were missed. The levels of care provided by different care homes, their admission criteria and the terminology used to define care homes vary between countries making the synthesis of data published on care homes problematic. For example, the definition of long-term care facilities applied in this review excluded studies in assisted living facilities and sheltered accommodation. Further discussion is needed to refine the terms used in this area and improve subsequent reporting. In addition, it is difficult to make meaningful comparisons between countries without considering the national policies and provision of care offered by care homes within a country’s wider health care system. The review also does not capture a resident’s living arrangement prior to admission that may affect the decision to enter a care home and their subsequent experience.

The focus of the review centered on studies that had explored a number of factors as their aim, without any prior hypothesis. Studies that explored the role of one factor on length of stay, such as depression or malnutrition, were excluded, for two reasons. Firstly, it would be impossible to develop a search strategy that could identify all factors associated with length of stay, without first having a basis on which to justify the inclusion of search terms for each factor. Secondly, the number of studies identified would be very large and difficult to synthesize.

The interpretation of the results is also limited by the limitations of the individual studies. Most studies only collected data at baseline, with follow-up restricted to the outcome measure of time until death. It is possible that changes in time-dependent characteristics, such as cognitive impairment, which may get progressively worse, were missed. There were numerous measures used to assess factors, for example, at least 6 tools were used to assess physical functioning and 12 for cognitive functioning; however, the analytical approach used in this review allowed these findings to be combined and weighted into a meaningful measure of association and methodological quality. Finally, the review did not perform study selection and study extraction in duplicate on the full dataset.

### Implications for further research

The study highlighted the varied trajectories of care home residents approaching end of life and the need for flexible, appropriate palliative care provision to accommodate different trajectories. This review has synthesized factors associated with variation in length of stay in care homes, and identified similar homogeneity within the care home population from admission to death, which is not yet fully understood.

Increasingly, care homes are taking on a complex role within the wider health system, catering for the diversities of an ageing population that can no longer live in the community. At one end of the spectrum, care homes are acting as proxy hospices for short-stay residents approaching end of life. At the other end, care homes are accommodating residents with cognitive impairments who may survive for many years post-admission. Supporting care homes in negotiating these two roles; delivering palliative care for short-stay residents while simultaneously providing a residential home for long-stay residents, in the same space, is imperative and requires further research. In addition, further thought should be given to the suitability of care homes in catering for such a wide variation in needs. The potential for other types of services, such as specialist dementia care units and assisted living facilities, in providing care for subgroups of care home residents could be examined, although there is debate as to whether such services provide better care [43, 44].

The findings of this review have identified numerous questions requiring further investigation. Firstly, further research is needed to explore the relationship between factors associated with care home admission and factors associated with length of stay. Longitudinal studies which follow community-dwelling older adults post-admission are required to fully understand this relationship. Secondly, in this review, characteristics related to the care home were only collected in eight studies, further data on variation in length of stay and care home-related factors could identify ways to improve the delivery of care. It is imperative that research on care home residents contextualizes the data within the long-term care setting to inform the generalizability of the findings internationally. Finally, inclusion and identification of care home residents in existing national datasets would allow comparisons within and between countries, and enable time-dependent variables to be monitored.

## Conclusion

Care home residents remain a growing, diverse population. An understanding of the factors associated with shorter and longer lengths of residence within care homes can be used to inform residents and their families about their potential use of health care services. Clinicians can use these findings to inform treatment decisions for older residents residing in care homes, and if required, organize palliative care. On a wider scale, policy-makers can use these findings to inform service planning for the future and to identify facilities in which lengths of stay deviate from the expected. Good quality, replicable research on the health needs of care home residents is a priority, now and in the future.

## Additional files

Additional file 1: Criteria for assessing methodological quality of studies. (DOCX 34 kb)

Additional file 2: Factors associated with length of stay before death in care home residents in all studies and split between high-, moderate- and low-quality studies. (DOCX 45 kb)

Additional file 3: Factors associated with length of stay before death in care home residents in all studies and split between high-, moderate- and low-quality studies-limited to 1 year follow-up. (DOCX 47 kb)

Additional file 4: Factors associated with length of stay before death in care home residents—data extracted from included papers. (DOCX 349 kb)

## Abbreviations

BMI: Body mass index
COPD: Chronic obstructive pulmonary disease
