# Supplementary material for: Factors associated with length of stay in care homes: a systematic review of international literature
Source: Syst Rev. 2019 Feb 20;8:56. doi: 10.1186/s13643-019-0973-0 (PMC6381725; doi:10.1186/s13643-019-0973-0)
Supplement: Supplementary file 1 — Criteria for assessing methodological quality of studies. (DOCX 34 kb) [file 13643_2019_973_MOESM1_ESM.docx]

Additional file 1: Criteria for assessing methodological quality of studies.

| **Item** | **Question** | **Notes** |
| --- | --- | --- |
| 1 | Study sample is nationally or regionally representative of the care home population of the country. | **AWARD 1 IF:**   - More than one LTCF which is representative of the area.   **AWARD 0 IF:**   - There is one site/LTCF OR - There are a number of sites with the same characteristics i.e. ran by social services, Medicaid |
| 2 | Sample inclusion and/or exclusion criteria are formulated for care homes: Facility types (i.e. nursing home and/or other facilities) is reported. | **AWARD 1 IF:**   - There is discussion of the type of care home(s) and the services offered.     **AWARD 0 IF:**   - There is no discussion of the care home facility. |
| 3 | Sample inclusion and/or exclusion criteria are formulated for care home residents. | **AWARD 1 IF:**   - There is discussion of the resident age, or admission status or diagnoses.   **AWARD 0 IF:**   - There is little or no discussion of resident inclusion criteria |
| 4 | Information on participant’s lost-to-follow-up is reported. | **AWARD 1 IF:**   - The number included in the baseline and final sample is the same OR - The dropouts are discussed.   **AWARD 0 IF:**   - The numbers differ without explanation. |
| 5 | The process of data collection is described (e.g. interview or self-report). | **AWARD 1 IF:**   - The characteristics of the dataset or the process of data collection is discussed   **AWARD 0 IF:**   - There is no discussion of how the data was collected |
| 6 | Training and quality control methods for interviewers’ technique are applied. | **AWARD 1 IF:**   - The validity of the dataset is discussed, or the training/ experience of the data collectors is discussed.   **AWARD 0 IF:**   - There is no discussion of training/ data quality OR - The role of the data collector is discussed i.e. geriatrician, but no training OR - The data collector is described as trained or experienced, with no further information. |
| 7 | Definition of the outcome criteria of death is provided. | **AWARD 1 IF:**   - The follow up period is discussed AND there is information on how death is notified, i.e. through death certificates or on a dataset.   **AWARD 0 IF:**   - Only one or neither of these are discussed. |
| 8 | Descriptive data are provided on survival (e.g. number of  individuals died/survived time to death). | **AWARD 1 IF:**   - The study reports the number of residents who died during follow up.   **AWARD 0 IF:**   - There is no discussion of the overall number who survived. |
| 9 | Characteristics of study participants (socio-demographic, clinical, social) are given. | **AWARD 1 IF:**   - The characteristics of the dataset at baseline are provided, and discuss at least two of the three: socio-demographic, clinical, social characteristics.   **AWARD 0 IF:**   - None are discussed, or the discussion is brief. |
| 10 | For each variable of interest, sources of data and details of methods of assessment are given. | **AWARD 1 IF:**   - The measurement and collection of all the variables included in the study is discussed.   **AWARD 0 IF:**   - There is no or little discussion of the variables and how they are measured/ collected. |
| 11 | Reliability and/or validity of study instruments is reported. | **AWARD 1 IF:**   - The reliability and/or validity of at least one measure used is reported.   **AWARD 0 IF:**   - None are reported. |
| 12 | Detailed description of statistical analyses is given. | **AWARD 1 IF:**   - There is a discussion of the statistical measures used.   **AWARD 0 IF:**   - The discussion is absent or brief. |
| 13 | Information on non-significant predictor variables is reported. | **AWARD 1 IF:**   - There non-significant results are reported either at univariate or multivariate analysis.   **AWARD 0 IF:**   - No significance is reported in the paper |
| 14 | Precision of estimates is given (e.g. 95% confidence interval). | **AWARD 1 IF:**   - A 95% confidence interval or equivalent is provided in the data extracted.   **AWARD 0 IF:**   - No 95% confidence interval or equivalent is provided. |

Current version modified from original developed by Luppa M, Luck T, Weyerer S, König H-H, Brähler E, Riedel-Heller S. Prediction of institutionalization in the elderly. A systematic review. Age and Ageing. 2010;39(1):31-8
