# Supplementary material for: Factors associated with length of stay in care homes: a systematic review of international literature
Source: Syst Rev. 2019 Feb 20;8:56. doi: 10.1186/s13643-019-0973-0 (PMC6381725; doi:10.1186/s13643-019-0973-0)
Supplement: Supplementary file 2 — Factors associated with length of stay before death in care home residents in all studies and split between high-, moderate- and low-quality studies. (DOCX 45 kb) [file 13643_2019_973_MOESM2_ESM.docx]

Additional file 2: Factors associated with length of stay before death in care home residents in all studies and split between high, moderate and low quality studies.

|  | **All** | | | | | | | **High** | | | | | **Medium** | | | | | **Low** | | | | |
| --- | --- | --- | --- | --- | --- | --- | --- | --- | --- | --- | --- | --- | --- | --- | --- | --- | --- | --- | --- | --- | --- | --- |
| **Predictor** | **Total** | **+** | | **NS** | | **-** | | **Total** | | **+** | **NS** | **-** | **Total** | | **+** | **NS** | **-** | **Total** | | **+** | **NS** | **-** |
|  | **N** | **N** | **%** | **N** | **%** | **N** | **%** | **N** | **%** | **N** | **N** | **N** | **N** | **%** | **N** | **N** | **N** | **N** | **%** | **N** | **N** | **N** |
| Admission source - care home /assisted living | 10 | 1 | 10 | 7 | 70 | 2 | 20 | 2 | 20 | 0 | 2 | 0 | 8 | 80 | 1 | 5 | 2 | 0 | 0 | 0 | 0 | 0 |
| Admission source - home | 5 | 0 | 0 | 3 | 60 | 2 | 40 | 2 | 40 | 0 | 2 | 0 | 3 | 60 | 0 | 1 | 2 | 0 | 0 | 0 | 0 | 0 |
| Admission source - hospital | 14 | 6 | 43 | 6 | 43 | 2 | 14 | 3 | 21 | 2 | 1 | 0 | 11 | 79 | 4 | 5 | 2 | 0 | 0 | 0 | 0 | 0 |
| Age | 53 | 35 | 66 | 18 | 34 | 0 | 0 | 14 | 26 | 8 | 6 | 0 | 37 | 70 | 25 | 12 | 0 | 2 | 4 | 2 | 0 | 0 |
| Alcohol | 4 | 0 | 0 | 3 | 75 | 1 | 25 | 3 | 75 | 0 | 2 | 1 | 1 | 25 | 0 | 1 | 0 | 0 | 0 | 0 | 0 | 0 |
| Anaemia | 8 | 1 | 13 | 7 | 88 | 0 | 0 | 4 | 50 | 1 | 3 | 0 | 4 | 50 | 0 | 4 | 0 | 0 | 0 | 0 | 0 | 0 |
| Anxiety | 3 | 0 | 0 | 3 | 100 | 0 | 0 | 0 | 0 | 0 | 0 | 0 | 3 | 100 | 0 | 3 | 0 | 0 | 0 | 0 | 0 | 0 |
| Arthritis | 8 | 0 | 0 | 8 | 100 | 0 | 0 | 5 | 63 | 0 | 5 | 0 | 3 | 38 | 0 | 3 | 0 | 0 | 0 | 0 | 0 | 0 |
| Behaviour problems | 36 | 3 | 8 | 33 | 92 | 0 | 0 | 8 | 22 | 1 | 7 | 0 | 28 | 78 | 2 | 26 | 0 | 0 | 0 | 0 | 0 | 0 |
| Biochemical indicators* | 97 | 8 | 8 | 84 | 87 | 5 | 5 | 4 | 4 | 1 | 3 | 0 | 93 | 96 | 7 | 81 | 5 | 0 | 0 | 0 | 0 | 0 |
| Blood pressure and hypertension | 19 | 1 | 5 | 18 | 95 | 0 | 0 | 7 | 37 | 0 | 7 | 0 | 12 | 63 | 1 | 11 | 0 | 0 | 0 | 0 | 0 | 0 |
| Cancer | 27 | 16 | 59 | 11 | 41 | 0 | 0 | 10 | 37 | 5 | 5 | 0 | 17 | 63 | 11 | 6 | 0 | 0 | 0 | 0 | 0 | 0 |
| Cardiovascular disorders* | 78 | 21 | 27 | 54 | 69 | 3 | 4 | 29 | 37 | 6 | 22 | 1 | 49 | 63 | 15 | 32 | 2 | 0 | 0 | 0 | 0 | 0 |
| Care home characteristics - nursing | 10 | 8 | 80 | 2 | 20 | 0 | 0 | 4 | 40 | 2 | 2 | 0 | 6 | 60 | 6 | 0 | 0 | 0 | 0 | 0 | 0 | 0 |
| Care home characteristics - ownership | 7 | 2 | 29 | 2 | 29 | 3 | 43 | 0 | 0 | 0 | 0 | 0 | 7 | 100 | 2 | 2 | 3 | 0 | 0 | 0 | 0 | 0 |
| Care home characteristics - size | 3 | 0 | 0 | 3 | 100 | 0 | 0 | 0 | 0 | 0 | 0 | 0 | 3 | 100 | 0 | 3 | 0 | 0 | 0 | 0 | 0 | 0 |
| Clinical intervention - aspiration | 5 | 0 | 0 | 5 | 100 | 0 | 0 | 2 | 40 | 0 | 2 | 0 | 3 | 60 | 0 | 3 | 0 | 0 | 0 | 0 | 0 | 0 |
| Clinical intervention - oxygen therapy | 4 | 4 | 100 | 0 | 0 | 0 | 0 | 3 | 75 | 3 | 0 | 0 | 1 | 25 | 1 | 0 | 0 | 0 | 0 | 0 | 0 | 0 |
| Cognitive function* | 59 | 14 | 24 | 41 | 69 | 4 | 7 | 10 | 17 | 2 | 8 | 0 | 49 | 83 | 12 | 33 | 4 | 0 | 0 | 0 | 0 | 0 |
| Communication problems | 11 | 2 | 18 | 9 | 82 | 0 | 0 | 3 | 27 | 0 | 3 | 0 | 8 | 73 | 2 | 6 | 0 | 0 | 0 | 0 | 0 | 0 |
| Contact with primary care - number of contacts | 3 | 2 | 67 | 1 | 33 | 0 | 0 | 2 | 67 | 2 | 0 | 0 | 1 | 33 | 0 | 1 | 0 | 0 | 0 | 0 | 0 | 0 |
| Dehydration | 8 | 1 | 13 | 7 | 88 | 0 | 0 | 4 | 50 | 1 | 3 | 0 | 4 | 50 | 0 | 4 | 0 | 0 | 0 | 0 | 0 | 0 |
| Dementia or Alzheimer’s Disease | 32 | 3 | 9 | 27 | 84 | 2 | 6 | 10 | 31 | 1 | 8 | 1 | 21 | 66 | 2 | 18 | 1 | 1 | 3 | 0 | 1 | 0 |
| Depression | 19 | 5 | 26 | 14 | 74 | 0 | 0 | 4 | 21 | 1 | 3 | 0 | 15 | 79 | 4 | 11 | 0 | 0 | 0 | 0 | 0 | 0 |
| Diabetes | 26 | 10 | 38 | 16 | 62 | 0 | 0 | 10 | 38 | 2 | 8 | 0 | 16 | 62 | 8 | 8 | 0 | 0 | 0 | 0 | 0 | 0 |
| Education - low | 9 | 1 | 11 | 7 | 78 | 1 | 11 | 3 | 33 | 1 | 1 | 1 | 6 | 67 | 0 | 6 | 0 | 0 | 0 | 0 | 0 | 0 |
| Ethnicity - white | 15 | 0 | 0 | 11 | 73 | 4 | 27 | 11 | 73 | 0 | 9 | 2 | 4 | 27 | 0 | 2 | 2 | 0 | 0 | 0 | 0 | 0 |
| Falls and fractures | 30 | 2 | 7 | 23 | 77 | 5 | 17 | 13 | 43 | 0 | 11 | 2 | 17 | 57 | 2 | 12 | 3 | 0 | 0 | 0 | 0 | 0 |
| Feeding - appetite | 11 | 5 | 45 | 6 | 55 | 0 | 0 | 2 | 18 | 2 | 0 | 0 | 9 | 82 | 3 | 6 | 0 | 0 | 0 | 0 | 0 | 0 |
| Feeding - feeding tube, help with feeding or diet | 21 | 6 | 29 | 13 | 62 | 2 | 10 | 7 | 33 | 2 | 5 | 0 | 14 | 67 | 4 | 8 | 2 | 0 | 0 | 0 | 0 | 0 |
| Feeding - swallowing problems | 8 | 2 | 25 | 6 | 75 | 0 | 0 | 1 | 13 | 0 | 1 | 0 | 7 | 88 | 2 | 5 | 0 | 0 | 0 | 0 | 0 | 0 |
| Fever | 4 | 0 | 0 | 4 | 100 | 0 | 0 | 1 | 25 | 0 | 1 | 0 | 3 | 75 | 0 | 3 | 0 | 0 | 0 | 0 | 0 | 0 |
| Gastrointestinal disorder | 4 | 0 | 0 | 3 | 75 | 1 | 25 | 0 | 0 | 0 | 0 | 0 | 4 | 100 | 0 | 3 | 1 | 0 | 0 | 0 | 0 | 0 |
| Gender - being female | 46 | 0 | 0 | 21 | 46 | 25 | 54 | 14 | 30 | 0 | 9 | 5 | 30 | 65 | 0 | 11 | 19 | 2 | 4 | 0 | 1 | 1 |
| General health | 21 | 12 | 57 | 9 | 43 | 0 | 0 | 7 | 33 | 5 | 2 | 0 | 14 | 67 | 7 | 7 | 0 | 0 | 0 | 0 | 0 | 0 |
| Genitourinary problems (including UTIs) | 11 | 2 | 18 | 9 | 82 | 0 | 0 | 6 | 55 | 1 | 5 | 0 | 5 | 45 | 1 | 4 | 0 | 0 | 0 | 0 | 0 | 0 |
| Hallucinations, delusions, wandering or delirium | 17 | 5 | 29 | 12 | 71 | 0 | 0 | 3 | 18 | 1 | 2 | 0 | 14 | 82 | 4 | 10 | 0 | 0 | 0 | 0 | 0 | 0 |
| Hearing impairment | 17 | 2 | 12 | 14 | 82 | 1 | 6 | 3 | 18 | 1 | 1 | 1 | 14 | 82 | 1 | 13 | 0 | 0 | 0 | 0 | 0 | 0 |
| Hospitalisation | 16 | 3 | 19 | 12 | 75 | 1 | 6 | 11 | 69 | 2 | 9 | 0 | 5 | 31 | 1 | 3 | 1 | 0 | 0 | 0 | 0 | 0 |
| Incontinence or catheter use | 34 | 13 | 38 | 21 | 62 | 0 | 0 | 8 | 24 | 3 | 5 | 0 | 26 | 76 | 10 | 16 | 0 | 0 | 0 | 0 | 0 | 0 |
| Infections | 18 | 4 | 22 | 14 | 78 | 0 | 0 | 0 | 0 | 0 | 0 | 0 | 12 | 67 | 2 | 10 | 0 | 6 | 33 | 2 | 4 | 0 |
| Involvement - activity | 11 | 0 | 0 | 10 | 91 | 1 | 9 | 0 | 0 | 0 | 0 | 0 | 11 | 100 | 0 | 10 | 1 | 0 | 0 | 0 | 0 | 0 |
| Involvement - children and visits | 8 | 2 | 25 | 6 | 75 | 0 | 0 | 2 | 25 | 0 | 2 | 0 | 6 | 75 | 2 | 4 | 0 | 0 | 0 | 0 | 0 | 0 |
| Involvement - social engagement | 6 | 0 | 0 | 4 | 67 | 2 | 33 | 1 | 17 | 0 | 0 | 1 | 5 | 83 | 0 | 4 | 1 | 0 | 0 | 0 | 0 | 0 |
| Kidney or liver disorder | 13 | 3 | 23 | 10 | 77 | 0 | 0 | 7 | 54 | 2 | 5 | 0 | 6 | 46 | 1 | 5 | 0 | 0 | 0 | 0 | 0 | 0 |
| Length of stay in care home** | 7 | 1 | 14 | 6 | 86 | 0 | 0 | 2 | 29 | 1 | 1 | 0 | 5 | 71 | 0 | 5 | 0 | 0 | 0 | 0 | 0 | 0 |
| Level of care | 6 | 2 | 33 | 3 | 50 | 1 | 17 | 3 | 50 | 1 | 2 | 0 | 3 | 50 | 1 | 1 | 1 | 0 | 0 | 0 | 0 | 0 |
| Marital status - being married | 15 | 2 | 13 | 12 | 80 | 1 | 7 | 8 | 53 | 2 | 5 | 1 | 7 | 47 | 0 | 7 | 0 | 0 | 0 | 0 | 0 | 0 |
| Marital status - not married (other) | 7 | 0 | 0 | 4 | 57 | 3 | 43 | 4 | 57 | 0 | 3 | 1 | 3 | 43 | 0 | 1 | 2 | 0 | 0 | 0 | 0 | 0 |
| Medicine use | 45 | 3 | 7 | 40 | 89 | 2 | 4 | 15 | 33 | 2 | 13 | 0 | 30 | 67 | 1 | 27 | 2 | 0 | 0 | 0 | 0 | 0 |
| Mobility | 10 | 5 | 50 | 5 | 50 | 0 | 0 | 4 | 40 | 2 | 2 | 0 | 6 | 60 | 3 | 3 | 0 | 0 | 0 | 0 | 0 | 0 |
| Multimorbidity or comorbidity | 12 | 3 | 25 | 9 | 75 | 0 | 0 | 4 | 33 | 0 | 4 | 0 | 8 | 67 | 3 | 5 | 0 | 0 | 0 | 0 | 0 | 0 |
| Musculoskeletal problem | 3 | 0 | 0 | 3 | 100 | 0 | 0 | 0 | 0 | 0 | 0 | 0 | 3 | 100 | 0 | 3 | 0 | 0 | 0 | 0 | 0 | 0 |
| Neurological disorders | 14 | 1 | 7 | 13 | 93 | 0 | 0 | 6 | 43 | 0 | 6 | 0 | 8 | 57 | 1 | 7 | 0 | 0 | 0 | 0 | 0 | 0 |
| Nutrition - low BMI or malnutrition | 31 | 16 | 52 | 14 | 45 | 1 | 3 | 5 | 16 | 5 | 0 | 0 | 26 | 84 | 11 | 14 | 1 | 0 | 0 | 0 | 0 | 0 |
| Pain | 13 | 1 | 8 | 12 | 92 | 0 | 0 | 5 | 38 | 0 | 5 | 0 | 8 | 62 | 1 | 7 | 0 | 0 | 0 | 0 | 0 | 0 |
| Parkinson’s disease | 12 | 3 | 25 | 8 | 67 | 1 | 8 | 6 | 50 | 2 | 4 | 0 | 6 | 50 | 1 | 4 | 1 | 0 | 0 | 0 | 0 | 0 |
| Physical functioning - poor* | 95 | 56 | 59 | 39 | 41 | 0 | 0 | 28 | 29 | 13 | 15 | 0 | 66 | 69 | 42 | 24 | 0 | 1 | 1 | 1 | 0 | 0 |
| Pressure ulcers | 15 | 9 | 60 | 6 | 40 | 0 | 0 | 5 | 33 | 4 | 1 | 0 | 10 | 67 | 5 | 5 | 0 | 0 | 0 | 0 | 0 | 0 |
| Previous care home use | 6 | 2 | 33 | 3 | 50 | 1 | 17 | 6 | 100 | 2 | 3 | 1 | 0 | 0 | 0 | 0 | 0 | 0 | 0 | 0 | 0 | 0 |
| Respiratory disorders/COPD | 31 | 15 | 48 | 16 | 52 | 0 | 0 | 9 | 29 | 4 | 5 | 0 | 22 | 71 | 11 | 11 | 0 | 0 | 0 | 0 | 0 | 0 |
| Restraint use | 6 | 1 | 17 | 5 | 83 | 0 | 0 | 2 | 33 | 1 | 1 | 0 | 4 | 67 | 0 | 4 | 0 | 0 | 0 | 0 | 0 | 0 |
| SES Facility - area deprivation | 10 | 7 | 70 | 3 | 30 | 0 | 0 | 1 | 10 | 0 | 1 | 0 | 9 | 90 | 7 | 2 | 0 | 0 | 0 | 0 | 0 | 0 |
| SES Resident - home ownership | 5 | 0 | 0 | 3 | 60 | 2 | 40 | 0 | 0 | 0 | 0 | 0 | 5 | 100 | 0 | 3 | 2 | 0 | 0 | 0 | 0 | 0 |
| SES Resident - payment support | 8 | 3 | 38 | 5 | 63 | 0 | 0 | 3 | 38 | 0 | 3 | 0 | 5 | 63 | 3 | 2 | 0 | 0 | 0 | 0 | 0 | 0 |
| Shortness of breath | 8 | 7 | 88 | 1 | 13 | 0 | 0 | 3 | 38 | 3 | 0 | 0 | 5 | 63 | 4 | 1 | 0 | 0 | 0 | 0 | 0 | 0 |
| Sleep - excess | 13 | 2 | 15 | 11 | 85 | 0 | 0 | 2 | 15 | 0 | 2 | 0 | 11 | 85 | 2 | 9 | 0 | 0 | 0 | 0 | 0 | 0 |
| Smoking | 6 | 1 | 17 | 5 | 83 | 0 | 0 | 1 | 17 | 0 | 1 | 0 | 5 | 83 | 1 | 4 | 0 | 0 | 0 | 0 | 0 | 0 |
| Stroke | 20 | 3 | 15 | 16 | 80 | 1 | 5 | 6 | 30 | 1 | 5 | 0 | 13 | 65 | 2 | 11 | 0 | 1 | 5 | 0 | 0 | 1 |
| Use of additional services | 16 | 0 | 0 | 16 | 100 | 0 | 0 | 13 | 81 | 0 | 13 | 0 | 3 | 19 | 0 | 3 | 0 | 0 | 0 | 0 | 0 | 0 |
| Vaccinations | 6 | 0 | 0 | 3 | 50 | 3 | 50 | 2 | 33 | 0 | 1 | 1 | 4 | 67 | 0 | 2 | 2 | 0 | 0 | 0 | 0 | 0 |
| Vision impairment | 15 | 3 | 20 | 12 | 80 | 0 | 0 | 3 | 20 | 1 | 2 | 0 | 12 | 80 | 2 | 10 | 0 | 0 | 0 | 0 | 0 | 0 |

Notes: (n) Number of studies which included the factors; (+) positive, statistically significant associations i.e. related to shorter stay; (-) negative statistically significant association i.e. related to longer stay; (ns) non-significant associations

*In cases where the number of results for a group of factors exceeds the number of cohorts (57), some studies collected data from multiple measures.

** Length of stay in care home before study baseline

BMI Body mass index
COPD Chronic obstructive pulmonary disease
SES Socioeconomic status
