# Supplementary material for: Factors associated with length of stay in care homes: a systematic review of international literature
Source: Syst Rev. 2019 Feb 20;8:56. doi: 10.1186/s13643-019-0973-0 (PMC6381725; doi:10.1186/s13643-019-0973-0)
Supplement: Supplementary file 3 — Factors associated with length of stay before death in care home residents in all studies and split between high-, moderate- and low-quality studies-limited to 1 year follow-up. (DOCX 47 kb) [file 13643_2019_973_MOESM3_ESM.docx]

Additional file 3: Factors associated with length of stay before death in care home residents in all studies and split between high, moderate and low quality studies-limited to one year follow up.

|  | All | | | | | | | High | | | | | | Medium | | | | | | Low | | | | |
| --- | --- | --- | --- | --- | --- | --- | --- | --- | --- | --- | --- | --- | --- | --- | --- | --- | --- | --- | --- | --- | --- | --- | --- | --- |
| **Predictor** | Total | + | | NS | | - | | Total | | + | NS | - | Total | | | + | NS | - | Total | | | + | NS | - |
|  | N | N | % | N | % | N | % | N | % | N | N | N | N | | % | N | N | N | N | | % | N | N | N |
| Admission source - care home /assisted living | 4 | 0 | 0 | 4 | 100 | 0 | 0 | 0 | 0 | 0 | 0 | 0 | 4 | | 100 | 0 | 4 | 0 | 0 | | 0 | 0 | 0 | 0 |
| Admission source - home | 3 | 0 | 0 | 2 | 67 | 1 | 33 | 2 | 67 | 0 | 2 | 0 | 1 | | 33 | 0 | 0 | 1 | 0 | | 0 | 0 | 0 | 0 |
| Admission source - hospital | 7 | 2 | 29 | 4 | 57 | 1 | 14 | 2 | 29 | 1 | 1 | 0 | 5 | | 71 | 1 | 3 | 1 | 0 | | 0 | 0 | 0 | 0 |
| Age | 25 | 14 | 56 | 11 | 44 | 0 | 0 | 11 | 44 | 6 | 5 | 0 | 14 | | 56 | 8 | 6 | 0 | 0 | | 0 | 0 | 0 | 0 |
| Alcohol | 3 | 0 | 0 | 2 | 67 | 1 | 33 | 3 | 100 | 0 | 2 | 1 | 0 | | 0 | 0 | 0 | 0 | 0 | | 0 | 0 | 0 | 0 |
| Anaemia | 6 | 1 | 17 | 5 | 83 | 0 | 0 | 4 | 67 | 1 | 3 | 0 | 2 | | 33 | 0 | 2 | 0 | 0 | | 0 | 0 | 0 | 0 |
| Arthritis | 6 | 0 | 0 | 6 | 100 | 0 | 0 | 4 | 67 | 0 | 4 | 0 | 2 | | 33 | 0 | 2 | 0 | 0 | | 0 | 0 | 0 | 0 |
| Behaviour problems | 21 | 0 | 0 | 21 | 100 | 0 | 0 | 5 | 24 | 0 | 5 | 0 | 16 | | 76 | 0 | 16 | 0 | 0 | | 0 | 0 | 0 | 0 |
| Biochemical indicators* | 42 | 6 | 14 | 36 | 86 | 0 | 0 | 4 | 10 | 1 | 3 | 0 | 38 | | 90 | 5 | 33 | 0 | 0 | | 0 | 0 | 0 | 0 |
| Blood pressure and hypertension | 10 | 0 | 0 | 10 | 100 | 0 | 0 | 6 | 60 | 0 | 6 | 0 | 4 | | 40 | 0 | 4 | 0 | 0 | | 0 | 0 | 0 | 0 |
| Cancer | 18 | 10 | 56 | 8 | 44 | 0 | 0 | 9 | 50 | 5 | 4 | 0 | 9 | | 50 | 5 | 4 | 0 | 0 | | 0 | 0 | 0 | 0 |
| Cardiovascular disorders* | 49 | 12 | 24 | 35 | 71 | 2 | 4 | 27 | 55 | 5 | 22 | 0 | 22 | | 45 | 7 | 13 | 2 | 0 | | 0 | 0 | 0 | 0 |
| Care home characteristics - nursing | 4 | 2 | 50 | 2 | 50 | 0 | 0 | 4 | 100 | 2 | 2 | 0 | 0 | | 0 | 0 | 0 | 0 | 0 | | 0 | 0 | 0 | 0 |
| Care home characteristics - ownership | 4 | 1 | 25 | 1 | 25 | 2 | 50 | 0 | 0 | 0 | 0 | 0 | 4 | | 100 | 1 | 1 | 2 | 0 | | 0 | 0 | 0 | 0 |
| Clinical intervention - aspiration | 3 | 0 | 0 | 3 | 100 | 0 | 0 | 1 | 33 | 0 | 1 | 0 | 2 | | 67 | 0 | 2 | 0 | 0 | | 0 | 0 | 0 | 0 |
| Clinical intervention - oxygen therapy | 4 | 4 | 100 | 0 | 0 | 0 | 0 | 3 | 75 | 3 | 0 | 0 | 1 | | 25 | 1 | 0 | 0 | 0 | | 0 | 0 | 0 | 0 |
| Cognitive function* | 37 | 10 | 27 | 26 | 70 | 1 | 3 | 6 | 16 | 1 | 5 | 0 | 31 | | 84 | 9 | 21 | 1 | 0 | | 0 | 0 | 0 | 0 |
| Communication problems | 10 | 1 | 10 | 9 | 90 | 0 | 0 | 3 | 30 | 0 | 3 | 0 | 7 | | 70 | 1 | 6 | 0 | 0 | | 0 | 0 | 0 | 0 |
| Dehydration | 8 | 1 | 13 | 7 | 88 | 0 | 0 | 4 | 50 | 1 | 3 | 0 | 4 | | 50 | 0 | 4 | 0 | 0 | | 0 | 0 | 0 | 0 |
| Dementia or Alzheimer’s Disease | 14 | 1 | 7 | 11 | 79 | 2 | 14 | 9 | 64 | 1 | 7 | 1 | 5 | | 36 | 0 | 4 | 1 | 0 | | 0 | 0 | 0 | 0 |
| Depression | 4 | 2 | 50 | 2 | 50 | 0 | 0 | 1 | 25 | 0 | 1 | 0 | 3 | | 75 | 2 | 1 | 0 | 0 | | 0 | 0 | 0 | 0 |
| Diabetes | 14 | 2 | 14 | 12 | 86 | 0 | 0 | 7 | 50 | 0 | 7 | 0 | 7 | | 50 | 2 | 5 | 0 | 0 | | 0 | 0 | 0 | 0 |
| Education - low | 4 | 1 | 25 | 2 | 50 | 1 | 25 | 2 | 50 | 1 | 0 | 1 | 2 | | 50 | 0 | 2 | 0 | 0 | | 0 | 0 | 0 | 0 |
| Ethnicity - white | 9 | 0 | 0 | 9 | 100 | 0 | 0 | 7 | 78 | 0 | 7 | 0 | 2 | | 22 | 0 | 2 | 0 | 0 | | 0 | 0 | 0 | 0 |
| Falls and fractures | 20 | 1 | 5 | 15 | 75 | 4 | 20 | 10 | 50 | 0 | 9 | 1 | 10 | | 50 | 1 | 6 | 3 | 0 | | 0 | 0 | 0 | 0 |
| Feeding - appetite | 9 | 5 | 56 | 4 | 44 | 0 | 0 | 2 | 22 | 2 | 0 | 0 | 7 | | 78 | 3 | 4 | 0 | 0 | | 0 | 0 | 0 | 0 |
| Feeding - feeding tube, help with feeding or diet | 18 | 6 | 33 | 11 | 61 | 1 | 6 | 6 | 33 | 2 | 4 | 0 | 12 | | 67 | 4 | 7 | 1 | 0 | | 0 | 0 | 0 | 0 |
| Feeding - swallowing problems | 8 | 2 | 25 | 6 | 75 | 0 | 0 | 1 | 13 | 0 | 1 | 0 | 7 | | 88 | 2 | 5 | 0 | 0 | | 0 | 0 | 0 | 0 |
| Fever | 4 | 0 | 0 | 4 | 100 | 0 | 0 | 1 | 25 | 0 | 1 | 0 | 3 | | 75 | 0 | 3 | 0 | 0 | | 0 | 0 | 0 | 0 |
| Gender - being female | 22 | 0 | 0 | 11 | 50 | 11 | 50 | 11 | 50 | 0 | 8 | 3 | 11 | | 50 | 0 | 3 | 8 | 0 | | 0 | 0 | 0 | 0 |
| General health | 16 | 9 | 56 | 7 | 44 | 0 | 0 | 5 | 31 | 3 | 2 | 0 | 11 | | 69 | 6 | 5 | 0 | 0 | | 0 | 0 | 0 | 0 |
| Genitourinary problems (including UTIs) | 8 | 1 | 13 | 7 | 88 | 0 | 0 | 6 | 75 | 1 | 5 | 0 | 2 | | 25 | 0 | 2 | 0 | 0 | | 0 | 0 | 0 | 0 |
| Hallucinations, delusions, wandering or delirium | 11 | 4 | 36 | 7 | 64 | 0 | 0 | 1 | 9 | 0 | 1 | 0 | 10 | | 91 | 4 | 6 | 0 | 0 | | 0 | 0 | 0 | 0 |
| Hearing impairment | 7 | 0 | 0 | 7 | 100 | 0 | 0 | 0 | 0 | 0 | 0 | 0 | 7 | | 100 | 0 | 7 | 0 | 0 | | 0 | 0 | 0 | 0 |
| Hospitalisation | 11 | 2 | 18 | 9 | 82 | 0 | 0 | 11 | 100 | 2 | 9 | 0 | 0 | | 0 | 0 | 0 | 0 | 0 | | 0 | 0 | 0 | 0 |
| Incontinence or catheter use | 18 | 8 | 44 | 10 | 56 | 0 | 0 | 5 | 28 | 2 | 3 | 0 | 13 | | 72 | 6 | 7 | 0 | 0 | | 0 | 0 | 0 | 0 |
| Infections | 15 | 2 | 13 | 13 | 87 | 0 | 0 | 12 | 80 | 2 | 10 | 0 | 3 | | 20 | 0 | 3 | 0 | 0 | | 0 | 0 | 0 | 0 |
| Involvement - activity | 7 | 0 | 0 | 6 | 86 | 1 | 14 | 0 | 0 | 0 | 0 | 0 | 7 | | 100 | 0 | 6 | 1 | 0 | | 0 | 0 | 0 | 0 |
| Involvement - children and visits | 3 | 0 | 0 | 3 | 100 | 0 | 0 | 2 | 67 | 0 | 2 | 0 | 1 | | 33 | 0 | 1 | 0 | 0 | | 0 | 0 | 0 | 0 |
| Kidney or liver disorder | 9 | 3 | 33 | 6 | 67 | 0 | 0 | 7 | 78 | 2 | 5 | 0 | 2 | | 22 | 1 | 1 | 0 | 0 | | 0 | 0 | 0 | 0 |
| Length of stay in care home** | 4 | 1 | 25 | 3 | 75 | 0 | 0 | 2 | 50 | 1 | 1 | 0 | 2 | | 50 | 0 | 2 | 0 | 0 | | 0 | 0 | 0 | 0 |
| Level of care | 5 | 2 | 40 | 3 | 60 | 0 | 0 | 3 | 60 | 1 | 2 | 0 | 2 | | 40 | 1 | 1 | 0 | 0 | | 0 | 0 | 0 | 0 |
| Marital status - being married | 8 | 2 | 25 | 6 | 75 | 0 | 0 | 6 | 75 | 2 | 4 | 0 | 2 | | 25 | 0 | 2 | 0 | 0 | | 0 | 0 | 0 | 0 |
| Medicine use * | 32 | 3 | 9 | 27 | 84 | 2 | 6 | 15 | 47 | 2 | 13 | 0 | 17 | | 53 | 1 | 14 | 2 | 0 | | 0 | 0 | 0 | 0 |
| Mobility | 5 | 2 | 40 | 3 | 60 | 0 | 0 | 2 | 40 | 1 | 1 | 0 | 3 | | 60 | 1 | 2 | 0 | 0 | | 0 | 0 | 0 | 0 |
| Multimorbidity or comorbidity | 3 | 0 | 0 | 3 | 100 | 0 | 0 | 3 | 100 | 0 | 3 | 0 | 0 | | 0 | 0 | 0 | 0 | 0 | | 0 | 0 | 0 | 0 |
| Neurological disorders | 11 | 0 | 0 | 11 | 100 | 0 | 0 | 6 | 55 | 0 | 6 | 0 | 5 | | 45 | 0 | 5 | 0 | 0 | | 0 | 0 | 0 | 0 |
| Nutrition - low BMI or malnutrition | 16 | 10 | 63 | 5 | 31 | 1 | 6 | 3 | 19 | 3 | 0 | 0 | 13 | | 81 | 7 | 5 | 1 | 0 | | 0 | 0 | 0 | 0 |
| Pain | 7 | 1 | 14 | 6 | 86 | 0 | 0 | 2 | 29 | 0 | 2 | 0 | 5 | | 71 | 1 | 4 | 0 | 0 | | 0 | 0 | 0 | 0 |
| Parkinson’s disease | 7 | 1 | 14 | 5 | 71 | 1 | 14 | 4 | 57 | 1 | 3 | 0 | 3 | | 43 | 0 | 2 | 1 | 0 | | 0 | 0 | 0 | 0 |
| Physical functioning - poor* | 54 | 35 | 65 | 19 | 35 | 0 | 0 | 23 | 43 | 9 | 14 | 0 | 31 | | 57 | 26 | 5 | 0 | 0 | | 0 | 0 | 0 | 0 |
| Pressure ulcers | 9 | 4 | 44 | 5 | 56 | 0 | 0 | 2 | 22 | 2 | 0 | 0 | 7 | | 78 | 2 | 5 | 0 | 0 | | 0 | 0 | 0 | 0 |
| Previous care home use | 6 | 2 | 33 | 3 | 50 | 1 | 17 | 6 | 100 | 2 | 3 | 1 | 0 | | 0 | 0 | 0 | 0 | 0 | | 0 | 0 | 0 | 0 |
| Respiratory disorders/COPD | 17 | 7 | 41 | 10 | 59 | 0 | 0 | 7 | 41 | 4 | 3 | 0 | 10 | | 59 | 3 | 7 | 0 | 0 | | 0 | 0 | 0 | 0 |
| Restraint use | 4 | 0 | 0 | 4 | 100 | 0 | 0 | 0 | 0 | 0 | 0 | 0 | 4 | | 100 | 0 | 4 | 0 | 0 | | 0 | 0 | 0 | 0 |
| SES Facility - area deprivation | 5 | 3 | 60 | 2 | 40 | 0 | 0 | 1 | 20 | 0 | 1 | 0 | 4 | | 80 | 3 | 1 | 0 | 0 | | 0 | 0 | 0 | 0 |
| SES Resident - payment support | 4 | 1 | 25 | 3 | 75 | 0 | 0 | 3 | 75 | 0 | 3 | 0 | 1 | | 25 | 1 | 0 | 0 | 0 | | 0 | 0 | 0 | 0 |
| Shortness of breath | 6 | 6 | 100 | 0 | 0 | 0 | 0 | 2 | 33 | 2 | 0 | 0 | 4 | | 67 | 4 | 0 | 0 | 0 | | 0 | 0 | 0 | 0 |
| Sleep - excess | 7 | 2 | 29 | 5 | 71 | 0 | 0 | 2 | 29 | 0 | 2 | 0 | 5 | | 71 | 2 | 3 | 0 | 0 | | 0 | 0 | 0 | 0 |
| Stroke | 6 | 0 | 0 | 6 | 100 | 0 | 0 | 3 | 50 | 0 | 3 | 0 | 3 | | 50 | 0 | 3 | 0 | 0 | | 0 | 0 | 0 | 0 |
| Use of additional services | 16 | 0 | 0 | 16 | 100 | 0 | 0 | 13 | 81 | 0 | 13 | 0 | 3 | | 19 | 0 | 3 | 0 | 0 | | 0 | 0 | 0 | 0 |
| Vaccinations | 4 | 0 | 0 | 2 | 50 | 2 | 50 | 2 | 50 | 0 | 1 | 1 | 2 | | 50 | 0 | 1 | 1 | 0 | | 0 | 0 | 0 | 0 |
| Vision impairment | 7 | 0 | 0 | 7 | 100 | 0 | 0 | 0 | 0 | 0 | 0 | 0 | 7 | | 100 | 0 | 7 | 0 | 0 | | 0 | 0 | 0 | 0 |

Notes: (n) Number of studies which included the factors; (+) positive, statistically significant associations i.e. related to shorter stay; (-) negative statistically significant association i.e. related to longer stay; (ns) non-significant associations

*In cases where the number of results for a group of factors exceeds the number of cohorts (26), some studies collected data from multiple measures.

** Length of stay in care home before study baseline

BMI Body mass index
COPD Chronic obstructive pulmonary disease
SES Socioeconomic status
