# Supplementary material for: Factors associated with length of stay in care homes: a systematic review of international literature
Source: Syst Rev. 2019 Feb 20;8:56. doi: 10.1186/s13643-019-0973-0 (PMC6381725; doi:10.1186/s13643-019-0973-0)
Supplement: Supplementary file 4 — Factors associated with length of stay before death in care home residents—data extracted from included papers. (DOCX 349 kb) [file 13643_2019_973_MOESM4_ESM.docx]

Additional file 4: Factors associated with length of stay before death in care home residents.

| **Author/year**  **Country**  **Study Design**  **QAS*** | **Sample- baseline/analysis** | **Inclusion criteria** | **Mean age (SD) Gender** | **Care home type / number** | **Methodological notes** | **Average length of stay** | **Predictors and severity direction** | **Risk** | **Risk measure** | **Sig** |
| --- | --- | --- | --- | --- | --- | --- | --- | --- | --- | --- |
| Bebbington et al, 2000(1-4)  England  Prospective follow up study  QAS: 10 | n=2,629/ n=2,191 | Local authority supported residents aged 65 years and older | 85 (SD NR)  78% female | Nursing and residential homes, n=NR | Baseline data collection over a three month period in 1995, follow up for six month, eighteen months, thirty months and forty two months – survey data provided by social services staff and care home managers, death registrations through the Office for National Statistics (ONS)  Analysis: Cox Proportional Hazard Models | 20.4.00% residents died within six months  38.8% residents died within eighteen months  60% residents died within thirty months  72.4% residents died within forty two months | **Death within six months:** |  |  |  |
|  |  |  |  |  |  |  | Age at admission 65-69 (ref) vs 70-74 | 0.8862 | OR | NS |
|  |  |  |  |  |  |  | Age at admission 65-69 (ref) vs 75-79 | 1.1668 | OR | NS |
|  |  |  |  |  |  |  | Age at admission 65-69 (ref) vs 80-84 | 1.1906 | OR | NS |
|  |  |  |  |  |  |  | Age at admission 65-69 (ref) vs 85+ | 1.4635 | OR | NS |
|  |  |  |  |  |  |  | Barthel Score: 13+ (ref) vs 0-4 | 4.4328 | OR | <.0.01 |
|  |  |  |  |  |  |  | Barthel Score: 13+ (ref) vs 5-8 | 2.0756 | OR | <.0.01 |
|  |  |  |  |  |  |  | Barthel Score: 13+ (ref) vs 9-12 | 1.7976 | OR | <.0.01 |
|  |  |  |  |  |  |  | Gender (male ref) | 0.7187 | OR | <.0.05 |
|  |  |  |  |  |  |  | Household composition: lived alone (ref) vs lived with others | 1.0839 | OR | NS |
|  |  |  |  |  |  |  | Household tenure: Owner occupied/mortgaged (ref) vs other | 0.8913 | OR | NS |
|  |  |  |  |  |  |  | Household tenure: Owner occupied/mortgaged (ref) vs privately rented | 1.3058 | OR | NS |
|  |  |  |  |  |  |  | Household tenure: Owner occupied/mortgaged (ref) vs rented from LA/NT/HA | 1.3114 | OR | <.0.10 |
|  |  |  |  |  |  |  | MDS Cognitive Scale: Intact (ref) vs mild impairment | 0.9042 | OR | NS |
|  |  |  |  |  |  |  | MDS Cognitive Scale: Intact (ref) vs severe impairment | 0.971 | OR | NS |
|  |  |  |  |  |  |  | Source of admission: domestic/sheltered household (ref) vs hospital | 1.261 | OR | <.0.10 |
|  |  |  |  |  |  |  | Source of admission: domestic/sheltered household (ref) vs nursing home | 0.665 | OR | NS |
|  |  |  |  |  |  |  | Source of admission: domestic/sheltered household (ref) vs other | 2.7942 | OR | <.0.05 |
|  |  |  |  |  |  |  | Source of admission: domestic/sheltered household (ref) vs residential care | 0.7083 | OR | NS |
|  |  |  |  |  |  |  | **Death within eighteen months:** |  |  |  |
|  |  |  |  |  |  |  | Age at admission 65-74 (ref) vs 75-84 | 1.49 | RR | <.0.01 |
|  |  |  |  |  |  |  | Age at admission 65-74 (ref) vs 85+ | 1.89 | RR | <.0.01 |
|  |  |  |  |  |  |  | Area of origin: Shire county (ref) vs London | 1.11 | RR | NS |
|  |  |  |  |  |  |  | Area of origin: Shire county (ref) vs Metropolitan District | 0.96 | RR | NS |
|  |  |  |  |  |  |  | Barthel Score: 13+ (ref) vs 0-4 | 2.23 | RR | <.0.01 |
|  |  |  |  |  |  |  | Barthel Score: 13+ (ref) vs 5-8 | 1.44 | RR | <.0.01 |
|  |  |  |  |  |  |  | Barthel Score: 13+ (ref) vs 9-12 | 1.47 | RR | <.0.01 |
|  |  |  |  |  |  |  | Cardiovascular | 1.03 | RR | NS |
|  |  |  |  |  |  |  | Dementia | 0.95 | RR | NS |
|  |  |  |  |  |  |  | Depression | 1.25 | RR | <.0.01 |
|  |  |  |  |  |  |  | Gender (male ref) | 0.73 | RR | <.0.01 |
|  |  |  |  |  |  |  | Incontinent (urine or faeces) | 0.95 | RR | NS |
|  |  |  |  |  |  |  | Initial placement: Local authority home (ref) vs nursing bed | 1.32 | RR | <.0.01 |
|  |  |  |  |  |  |  | Initial placement: Local authority home (ref) vs residential bed in private home | 0.92 | RR | <.0.01 |
|  |  |  |  |  |  |  | Malignancy | 2.34 | RR | <.0.01 |
|  |  |  |  |  |  |  | Respiratory | 1.32 | RR | <.0.01 |
|  |  |  |  |  |  |  | Source of admission: private household (ref) vs care home | 0.79 | RR | <.0.01 |
|  |  |  |  |  |  |  | Source of admission: private household (ref) vs hospital | 1.21 | RR | <.0.01 |
|  |  |  |  |  |  |  | Source of admission: private household (ref) vs other | 1.33 | RR | <.0.01 |
|  |  |  |  |  |  |  | Stroke | 0.95 | RR | NS |
|  |  |  |  |  |  |  | **Death within thirty months:** |  |  |  |
|  |  |  |  |  |  |  | Age at admission 65-74 (ref) vs 75-84 | 1.33 | RR | <.0.01 |
|  |  |  |  |  |  |  | Age at admission 65-74 (ref) vs 85+ | 1.79 | RR | <.0.01 |
|  |  |  |  |  |  |  | Area of origin: Shire county (ref) vs London | 0.94 | RR | NS |
|  |  |  |  |  |  |  | Area of origin: Shire county (ref) vs Metropolitan District | 0.93 | RR | NS |
|  |  |  |  |  |  |  | Barthel Score: 13+ (ref) vs 0-4 | 2.5 | RR | <.0.01 |
|  |  |  |  |  |  |  | Barthel Score: 13+ (ref) vs 5-8 | 1.51 | RR | <.0.01 |
|  |  |  |  |  |  |  | Barthel Score: 13+ (ref) vs 9-12 | 1.41 | RR | <.0.01 |
|  |  |  |  |  |  |  | Cardiovascular | 1.09 | RR | NS |
|  |  |  |  |  |  |  | Dementia | 0.97 | RR | NS |
|  |  |  |  |  |  |  | Depression | 1.04 | RR | NS |
|  |  |  |  |  |  |  | Gender (male ref) | 0.74 | RR | <.0.01 |
|  |  |  |  |  |  |  | Incontinent (urine or faeces) | 0.93 | RR | NS |
|  |  |  |  |  |  |  | Initial placement: Local authority home (ref) vs nursing bed | 1.54 | RR | <.0.01 |
|  |  |  |  |  |  |  | Initial placement: Local authority home (ref) vs residential bed in private home | 1.09 | RR | <.0.01 |
|  |  |  |  |  |  |  | Malignancy | 2.44 | RR | <.0.01 |
|  |  |  |  |  |  |  | MDS Cognitive Scale: Intact (ref) vs mild impairment | 1.14 | RR | NS |
|  |  |  |  |  |  |  | MDS Cognitive Scale: Intact (ref) vs severe impairment | 1.23 | RR | NS |
|  |  |  |  |  |  |  | Respiratory | 1.35 | RR | <.0.01 |
|  |  |  |  |  |  |  | Source of admission: private household (ref) vs care home | 0.88 | RR | <.0.01 |
|  |  |  |  |  |  |  | Source of admission: private household (ref) vs hospital | 1.21 | RR | <.0.01 |
|  |  |  |  |  |  |  | Source of admission: private household (ref) vs other | 1.45 | RR | <.0.01 |
|  |  |  |  |  |  |  | Stroke | 0.98 | RR | NS |
|  |  |  |  |  |  |  | **Death within forty two months:** |  |  |  |
|  |  |  |  |  |  |  | Age at admission 65-74 (ref) vs 75-84 | 1.42 | RR | 0.00 |
|  |  |  |  |  |  |  | Age at admission 65-74 (ref) vs 85+ | 1.99 | RR | 0.00 |
|  |  |  |  |  |  |  | Area of origin: Shire county (ref) vs London | 0.89 | RR | 0.08 |
|  |  |  |  |  |  |  | Area of origin: Shire county (ref) vs Metropolitan District | 0.89 | RR | 0.08 |
|  |  |  |  |  |  |  | Barthel Score: 13+ (ref) vs 0-4 | 1.89 | RR | 0.00 |
|  |  |  |  |  |  |  | Barthel Score: 13+ (ref) vs 5-8 | 1.3 | RR | 0.00 |
|  |  |  |  |  |  |  | Barthel Score: 13+ (ref) vs 9-12 | 1.27 | RR | 0.00 |
|  |  |  |  |  |  |  | Cardiovascular | 1.1 | RR | 0.15 |
|  |  |  |  |  |  |  | Dementia | 0.96 | RR | 0.49 |
|  |  |  |  |  |  |  | Depression | 1.04 | RR | 0.61 |
|  |  |  |  |  |  |  | Gender (male ref) | 0.75 | RR | 0.00 |
|  |  |  |  |  |  |  | Incontinent (urine or faeces) | 0.93 | RR | 0.28 |
|  |  |  |  |  |  |  | Initial placement: Local authority home (ref) vs nursing bed | 1.51 | RR | 0.00 |
|  |  |  |  |  |  |  | Initial placement: Local authority home (ref) vs residential bed in private home | 1.16 | RR | 0.00 |
|  |  |  |  |  |  |  | Malignancy | 2.34 | RR | 0.00 |
|  |  |  |  |  |  |  | MDS Cognitive Scale: Intact (ref) vs mild impairment | 1.15 | RR | 0.04 |
|  |  |  |  |  |  |  | MDS Cognitive Scale: Intact (ref) vs severe impairment | 1.25 | RR | 0.04 |
|  |  |  |  |  |  |  | Respiratory | 1.4 | RR | 0 |
|  |  |  |  |  |  |  | Source of admission: private household (ref) vs care home | 1.01 | RR | 0.12 |
|  |  |  |  |  |  |  | Source of admission: private household (ref) vs hospital | 1.13 | RR | 0.12 |
|  |  |  |  |  |  |  | Source of admission: private household (ref) vs other | 1.29 | RR | 0.12 |
|  |  |  |  |  |  |  | Stroke | 1.02 | RR | 0.77 |
| Breuer et al, 1998 (5)  USA  Retrospective cohort study  QAS: 8 | n=1,157/ n=1,145 | Residents who had resided in the nursing home for at least six months. | 86.8 (median) 59.7-102.6 (range)  80.3% female | Nursing home, n=1 | Baseline data collection 1986 to 1996, follow up for nine years and six months - medical charts reviewed by the research team.  Analysis: Cox Proportional Hazard Models | 92.3% residents died within nine years and six months | Age | 0.031 (0.004) | Coef (SE) | 0.0001 |
|  |  |  |  |  |  |  | Cardiac impairment | -0.16 (0.027) | Coef (SE) | 0.0001 |
|  |  |  |  |  |  |  | Endocrine/metabolic impairment | -0.09 (0.027) | Coef (SE) | 0.006 |
|  |  |  |  |  |  |  | Eye, ear, nose or throat impairment | NR | Coef (SE) | NS |
|  |  |  |  |  |  |  | Kidney impairment | NR | Coef (SE) | NS |
|  |  |  |  |  |  |  | Length of stay | NR | Coef (SE) | NS |
|  |  |  |  |  |  |  | Liver impairment | NR | Coef (SE) | NS |
|  |  |  |  |  |  |  | Lower gastrointestinal impairment | NR | Coef (SE) | NS |
|  |  |  |  |  |  |  | Marital status | NR | Coef (SE) | NS |
|  |  |  |  |  |  |  | Musculoskeletal-integumentary impairment | NR | Coef (SE) | NS |
|  |  |  |  |  |  |  | Neurological impairment | -0.06 (0.026) | Coef (SE) | 0.024 |
|  |  |  |  |  |  |  | Other genitourinary impairment | NR | Coef (SE) | NS |
|  |  |  |  |  |  |  | Psychiatric impairment - including dementia and depression | NR | Coef (SE) | NS |
|  |  |  |  |  |  |  | Respiratory impairment in females | -0.1 (0.037) | Coef (SE) | 0.0056 |
|  |  |  |  |  |  |  | Respiratory impairment in males | -0.3 (0.06) | Coef (SE) | 0.0001 |
|  |  |  |  |  |  |  | Sex (female ref) | -0.34 (0.067) | Coef (SE) | 0.0001 |
|  |  |  |  |  |  |  | Summary ADL Index | -0.12 (0.012) | Coef (SE) | 0.0001 |
|  |  |  |  |  |  |  | Upper gastrointestinal impairment | NR | Coef (SE) | NS |
|  |  |  |  |  |  |  | Vascular impairment | NR | Coef (SE) | NS |
| Carlson et al, 2001 (6)  USA  Prospective observational study  QAS: 9 | n=132 | Newly admitted residents aged 55 and older with dementia or AD | 79.4 (7.8)  72.1% female | LTCF, n=1 | Baseline data collection 1994 to 1996, follow up for five years, data collection through patient medical records and knowledgeable informants.  Analysis: Cox Proportional Hazard Models | 45.4% residents died within five years | Activity disturbance (BEHAVE-AD) | NR | HR (CI) | NS |
|  |  |  |  |  |  |  | ADLs | NR | HR (CI) | NS |
|  |  |  |  |  |  |  | Affective disturbance (BEHAVE-AD) | NR | HR (CI) | NS |
|  |  |  |  |  |  |  | Age | NR | HR (CI) | NS |
|  |  |  |  |  |  |  | Aggressiveness (BEHAVE-AD) | NR | HR (CI) | NS |
|  |  |  |  |  |  |  | Alzheimer's disease | NR | HR (CI) | NS |
|  |  |  |  |  |  |  | Anxiety and phobias (BEHAVE-AD) | NR | HR (CI) | NS |
|  |  |  |  |  |  |  | Behaviour (PGDRS) | NR | HR (CI) | NS |
|  |  |  |  |  |  |  | Delusions (BEHAVE-AD) (lower scores = better functioning) | 0.766 (0.613-0.958) | HR (CI) | 0.195 |
|  |  |  |  |  |  |  | Depression (CSD) | NR | HR (CI) | NS |
|  |  |  |  |  |  |  | Diurnal disturbance (BEHAVE-AD) | NR | HR (CI) | NS |
|  |  |  |  |  |  |  | Education | NR | HR (CI) | NS |
|  |  |  |  |  |  |  | Extrapyramidal Rating Scale | NR | HR (CI) | NS |
|  |  |  |  |  |  |  | General Medical Health Rating | 0.609 (0.416-0.891) | HR (CI) | 0.107 |
|  |  |  |  |  |  |  | Hallucinations (BEHAVE-AD) | NR | HR (CI) | NS |
|  |  |  |  |  |  |  | MMSE | NR | HR (CI) | NS |
|  |  |  |  |  |  |  | Orientation (PGDRS) | NR | HR (CI) | NS |
|  |  |  |  |  |  |  | Sex (female ref) | NR | HR (CI) | NS |
|  |  |  |  |  |  |  | Weight loss - more than 5% in past 6 weeks | NR | HR (CI) | NS |
|  |  |  |  |  |  |  | Years Ill | NR | HR (CI) | NS |
| Cereda et al, 2013 (7)  Italy  Prospective cohort study  QAS: 10 | n=395/ n=378 | Newly admitted residents aged over 65 with AD  NR | 80.8 (6.3) AD, 86.7 (7.6) other diagnosis, 87.6 (7.2) Dementia | LTCF, n=1 | Baseline data collection 2002 to 2009, follow up for nine years and seven months, data collection through patient medical records.  Analysis: Cox Proportional Hazard Models | 69.3% residents died within nine years and seven months | Admission diagnosis | NR | HR (CI) | NS |
|  |  |  |  |  |  |  | Age | 1.05 (1.03–1.07) | HR (CI) | NR |
|  |  |  |  |  |  |  | Albumin | 0.65 (0.47–0.89) | HR (CI) | NR |
|  |  |  |  |  |  |  | BMI | 0.97 (0.94–0.99) | HR (CI) | NR |
|  |  |  |  |  |  |  | Diabetes | 1.48 (1.05–2.07) | HR (CI) | NR |
|  |  |  |  |  |  |  | Functional status (Barthel Index) (lower score=greater dependence) | NR | HR (CI) | NS |
|  |  |  |  |  |  |  | Multiple comorbidities (additional comorbidity) | 1.13 (1.01–1.28) | HR (CI) | NR |
|  |  |  |  |  |  |  | Nutritional support during follow-up | 0.53 (0.31–0.90) | HR (CI) | NR |
|  |  |  |  |  |  |  | Sex (female ref) | 2.06 (1.54–2.76) | HR (CI) | NR |
| Chan et al, 2012 (8)  Hong Kong  Prospective cohort study    QAS: 9 | n=585 | Residents who had resided it the nursing home for at least 6 months | 85.6 (7.7)  65.3% female | Nursing home, n=12 | Baseline data collection April to June 2009, follow up for two years, data collection through patient medical records.    Analysis: Multivariate Cox Regression Model | 32.1% residents died within two years | Age =>91 years | 1.681 (1.101–2.565) | OR (CI) | <0.016 |
|  |  |  |  |  |  |  | Age 86–90 years | 1.589 (1.036–2.438) | OR (CI) | <0.034 |
|  |  |  |  |  |  |  | Barthel Index 0 - (total dependence) | 4.172 (2.612–6.664) | OR (CI) | <0.001 |
|  |  |  |  |  |  |  | Barthel Index 5 to 60 - (lower score=greater dependence) | 2.054 (1.375–3.069) | OR (CI) | <0.001 |
|  |  |  |  |  |  |  | Cerebrovascular disease | NR | OR (CI) | NS |
|  |  |  |  |  |  |  | Chronic liver disease | NR | OR (CI) | NS |
|  |  |  |  |  |  |  | Chronic pulmonary disease | NR | OR (CI) | NS |
|  |  |  |  |  |  |  | Chronic renal impairment | NR | OR (CI) | NS |
|  |  |  |  |  |  |  | Comorbidity (CCI) Score of >=4 | 2.374 (1.652–3.412) | OR (CI) | <0.001 |
|  |  |  |  |  |  |  | Congestive heart failure | NR | OR (CI) | NS |
|  |  |  |  |  |  |  | Dementia | NR | OR (CI) | NS |
|  |  |  |  |  |  |  | Diabetes | NR | OR (CI) | NS |
|  |  |  |  |  |  |  | Drinking | NR | OR (CI) | NS |
|  |  |  |  |  |  |  | Education | NR | OR (CI) | NS |
|  |  |  |  |  |  |  | Feeding status (non-oral feeding i.e. PEG) | NR | OR (CI) | NS |
|  |  |  |  |  |  |  | Ischemic heart disease | NR | OR (CI) | NS |
|  |  |  |  |  |  |  | Marital status | NR | OR (CI) | NS |
|  |  |  |  |  |  |  | Number of hospital admissions in preceding year: 1 | 1.816 (1.145–2.882) | OR (CI) | <0.003 |
|  |  |  |  |  |  |  | Number of hospital admissions in preceding year: 2 | 1.924 (1.090–3.396) | OR (CI) | <0.024 |
|  |  |  |  |  |  |  | Number of hospital admissions in preceding year:>= 3 | 1.981 (1.271–3.087) | OR (CI) | <0.011 |
|  |  |  |  |  |  |  | Number of medications | NR | OR (CI) | NS |
|  |  |  |  |  |  |  | Peripheral vascular disease | NR | OR (CI) | NS |
|  |  |  |  |  |  |  | Smoking | NR | OR (CI) | NS |
|  |  |  |  |  |  |  | Use of social security allowance | NR | OR (CI) | NS |
| Cohen-Mansfield et al, 1999 (9)  USA  Prospective cohort study    QAS: 9 | n=399 | Residents aged between 70 and 100 years | 85.7 (6.56)    77.7% female | Nursing home, n=1 | Baseline data collection 1985 to 1986, follow up for nine years, data collection through nursing home records and questionnaires to nursing staff.    Analysis: Multivariate Cox Regression Model | Median survival 33 months (2.75 years) | **Cohort 1 – cognitively intact (based on BCRS)** |  |  |  |
|  |  |  |  |  |  |  | ADL - bathing (RDRS-2) | NR | RR | NS |
|  |  |  |  |  |  |  | ADL - dressing (RDRS-2) | NR | RR | NS |
|  |  |  |  |  |  |  | ADL - eating (RDRS-2) | NR | RR | NS |
|  |  |  |  |  |  |  | ADL - grooming (RDRS-2) | NR | RR | NS |
|  |  |  |  |  |  |  | ADL - toileting (RDRS-2) | NR | RR | NS |
|  |  |  |  |  |  |  | ADL - walking (RDRS-2) | NR | RR | NS |
|  |  |  |  |  |  |  | ADLs (RDRS-2) | NR | RR | NS |
|  |  |  |  |  |  |  | Age | NR | RR | NS |
|  |  |  |  |  |  |  | Agitation (CMAI) | NR | RR | NS |
|  |  |  |  |  |  |  | Appetite | NR | RR | NS |
|  |  |  |  |  |  |  | Cognitive function (BCRS) | NR | RR | NS |
|  |  |  |  |  |  |  | Dementia | NR | RR | NS |
|  |  |  |  |  |  |  | Depression (DRS) | NR | RR | NS |
|  |  |  |  |  |  |  | Frequency of wake at night (SPQ) | NR | RR | NS |
|  |  |  |  |  |  |  | Hearing problem | NR | RR | NS |
|  |  |  |  |  |  |  | High levels of screaming (CMAI item) | NR | RR | NS |
|  |  |  |  |  |  |  | Hours of sleep (SPQ) | NR | RR | NS |
|  |  |  |  |  |  |  | Incontinence | NR | RR | NS |
|  |  |  |  |  |  |  | Number of diagnoses | 1.76 | RR | <.05 |
|  |  |  |  |  |  |  | Number of falls | NR | RR | NS |
|  |  |  |  |  |  |  | Number of medications | NR | RR | NS |
|  |  |  |  |  |  |  | Pain | NR | RR | NS |
|  |  |  |  |  |  |  | Physically nonaggressive behaviours | 3 | RR | <.05 |
|  |  |  |  |  |  |  | Quality and size of social network (HHSNRS) | NR | RR | NS |
|  |  |  |  |  |  |  | Sex (female ref) | 1.65 | RR | <.05 |
|  |  |  |  |  |  |  | Surgery in the last 2 years | NR | RR | NS |
|  |  |  |  |  |  |  | Years in nursing home | NR | RR | NS |
|  |  |  |  |  |  |  | **Cohort 2 – cognitively impaired (based on BCRS)** |  |  |  |
|  |  |  |  |  |  |  | ADL - bathing (RDRS-2) | NR | RR | NS |
|  |  |  |  |  |  |  | ADL - dressing (RDRS-2) | NR | RR | NS |
|  |  |  |  |  |  |  | ADL - eating (RDRS-2) | NR | RR | NS |
|  |  |  |  |  |  |  | ADL - grooming (RDRS-2) | NR | RR | NS |
|  |  |  |  |  |  |  | ADL - toileting (RDRS-2) | NR | RR | NS |
|  |  |  |  |  |  |  | ADL - walking (RDRS-2) | NR | RR | NS |
|  |  |  |  |  |  |  | ADLs (RDRS-2) | 1.6 | RR | <.01 |
|  |  |  |  |  |  |  | Age | 1.04 | RR | <.01 |
|  |  |  |  |  |  |  | Agitation (CMAI) | NR | RR | NS |
|  |  |  |  |  |  |  | Appetite | NR | RR | NS |
|  |  |  |  |  |  |  | Cognitive function (BCRS) | NR | RR | NS |
|  |  |  |  |  |  |  | Dementia | NR | RR | NS |
|  |  |  |  |  |  |  | Depression (Depression Rating Scale) | NR | RR | NS |
|  |  |  |  |  |  |  | Frequency of wake at night (SPQ) | NR | RR | NS |
|  |  |  |  |  |  |  | Hearing problem | NR | RR | NS |
|  |  |  |  |  |  |  | High levels of screaming (CMAI) | 1.39 | RR | <.05 |
|  |  |  |  |  |  |  | Hours of sleep (SPQ) | NR | RR | NS |
|  |  |  |  |  |  |  | Incontinence | NR | RR | NS |
|  |  |  |  |  |  |  | Number of diagnoses | NR | RR | NS |
|  |  |  |  |  |  |  | Number of falls | NR | RR | NS |
|  |  |  |  |  |  |  | Number of medications | 1.21 | RR | NS |
|  |  |  |  |  |  |  | Pain | NR | RR | NS |
|  |  |  |  |  |  |  | Physically nonaggressive behaviours | NR | RR | NS |
|  |  |  |  |  |  |  | Quality and size of social network (HHSNRS) | NR | RR | NS |
|  |  |  |  |  |  |  | Sex (female ref) | NR | RR | NS |
|  |  |  |  |  |  |  | Surgery in the last 2 years | NR | RR | NS |
|  |  |  |  |  |  |  | Years in nursing home | NR | RR | NS |
| Connolly, Broad and Boyd, 2014 (10)  New Zealand  Prospective follow up study    QAS: 11 | n=6,271/ n=380 | Residents admitted within 30 days prior to the baseline | 86 (median)    69.7% female | Residential aged care, n=152 | Baseline data collection 2008, follow up for 6 months, data collected from facility staff. Variables associated with time to death in residents with length of stay under 1 month.  Analysis: Cox Proportional Hazard Models | 20% residents died within 6 months | Acute hospital into long-term hospital care vs other pathway (ref) | 2.02 (1.2- 3.3) | HR (CI) | < 0.05 |
|  |  |  |  |  |  |  | Age | NR | HR (CI) | NS |
|  |  |  |  |  |  |  | Ethnicity | NR | HR (CI) | NS |
|  |  |  |  |  |  |  | Level of care | NR | HR (CI) | NS |
|  |  |  |  |  |  |  | Marital status | NR | HR (CI) | NS |
|  |  |  |  |  |  |  | Number of admissions in 2 years prior, none (ref) vs 3+ | 5.40 (1.6-17.6) | HR (CI) | < 0.05 |
|  |  |  |  |  |  |  | Number of admissions in 2 years prior, none (ref) vs 1 | 4.60 (1.4-15.7) | HR (CI) | < 0.05 |
|  |  |  |  |  |  |  | Number of admissions in 2 years prior, none (ref) vs 2 | 4.50 (1.3-15.5) | HR (CI) | < 0.05 |
|  |  |  |  |  |  |  | Previous residence | NR | HR (CI) | NS |
|  |  |  |  |  |  |  | Recent medical history | NR | HR (CI) | NS |
|  |  |  |  |  |  |  | Sex | NR | HR (CI) | NS |
|  |  |  |  |  |  |  | Special nursing care (inc. tube care or diabetes management) | NR | HR (CI) | NS |
|  |  |  |  |  |  |  | Unable to manage personal care at all vs some or no assistance required (ref) | 1.90 (1.7- 3.07) | HR (CI) | < 0.05 |
|  |  |  |  |  |  |  | Unscheduled GP visit during prior 2 weeks- none (ref) vs 1+ | 1.90 (1.2- 3.2) | HR (CI) | < 0.05 |
|  |  |  |  |  |  |  | Urgent visit to hospital in previous 2 weeks | NR | HR (CI) | NS |
| Dale et al, 2001 (11)  England  Retrospective case note audit - matched case control    QAS: 10 | n=507/ n=90 | Deceased residents and living residents matched on age, sex and time in care home | 75.1 (male) / 81.1 (females) (SD NR)    64.3% female | Nursing homes, n=59 | Baseline data collection 1994 to 1995, follow up for one year, data collected from the Manchester Social Services Department Community Assessment    Analysis: Cox Proportional Hazard Models | 32.6% residents died within one year | ADLs - (greater scores=greater impairment) | 1.02 (1.00-1.03) | HR (CI) | 0.25 |
|  |  |  |  |  |  |  | Age | 1.02 (1.00-1.05) | HR (CI) | 0.027 |
|  |  |  |  |  |  |  | Appetite - feeding tube/fluids only | 4.05 (1.40-1.73) | HR (CI) | 0.009 |
|  |  |  |  |  |  |  | Appetite - nil by mouth/anorexic | 2.06 (0.87-4.88) | HR (CI) | 0.098 |
|  |  |  |  |  |  |  | Appetite - poor | 2.16 (1.59-2.93) | HR (CI) | 0.001 |
|  |  |  |  |  |  |  | Being cooperative | NR | HR (CI) | NS |
|  |  |  |  |  |  |  | Build - above / below average | NR | HR (CI) | NS |
|  |  |  |  |  |  |  | Cardiovascular disease | NR | HR (CI) | NS |
|  |  |  |  |  |  |  | Dementia (CDR) (greater scores=greater impairment) | 10.5 (1.02-1.08) | HR (CI) | 0.003 |
|  |  |  |  |  |  |  | Excess sleeping | NR | HR (CI) | NS |
|  |  |  |  |  |  |  | Malignancy | 3.04 (1.00-4.67) | HR (CI) | 0.001 |
|  |  |  |  |  |  |  | Marital status | NR | HR (CI) | NS |
|  |  |  |  |  |  |  | Number of drugs | 1.07 (1.01-1.34) | HR (CI) | 0.144 |
|  |  |  |  |  |  |  | Obstructive airway disease | 1.67 (1.11-2.52) | HR (CI) | 0.013 |
|  |  |  |  |  |  |  | Placement prior to admission - geriatric long stay bed | 0.81 (0.43-1.52) | HR (CI) | 0.508 |
|  |  |  |  |  |  |  | Placement prior to admission - geriatric medical bed | 1.16 (0.76-1.77) | HR (CI) | 0.492 |
|  |  |  |  |  |  |  | Placement prior to admission - hospice | 9.80 (3.30-29.13) | HR (CI) | 0.001 |
|  |  |  |  |  |  |  | Placement prior to admission - medical bed | 0.95 (0.43-2.12) | HR (CI) | 0.909 |
|  |  |  |  |  |  |  | Placement prior to admission - nursing home | 0.87 (0.48-1.56) | HR (CI) | 0.636 |
|  |  |  |  |  |  |  | Placement prior to admission - orthopaedic bed | 1.53 (0.76-3.11) | HR (CI) | 0.243 |
|  |  |  |  |  |  |  | Placement prior to admission - psychiatric bed | 1.63 (0.71-3.71) | HR (CI) | 0.248 |
|  |  |  |  |  |  |  | Placement prior to admission - psychiatric long stay bed | 0.47 (0.10-2.15) | HR (CI) | 0.33 |
|  |  |  |  |  |  |  | Pressure ulcers - Waterlow score (greater scores=greater risk) | 1.03 (1.00-1.06) | HR (CI) | 0.028 |
|  |  |  |  |  |  |  | Reason for admission | NR | HR (CI) | NS |
|  |  |  |  |  |  |  | Sex (male ref) | 0.49 (0.36-0.66) | HR (CI) | 0.001 |
| Dontas et al, 1991 (12)  Greece  Prospective study  QAS: 8 | n=408 | Newly admitted residents aged 68 years and over | 79.1 (7.1) (male) / 79.4 (6.2) (females)    65.4% female | Residential home, n=1 | Baseline data collection 1978 to 1983, follow up for eleven years, data collected by study authors and care home staff.  Analysis: Cox Proportional Hazard Models | 78.6% male and 78.% female residents died within eleven years | Age | 1.10 (0.99-1.22) | MRR (CI) | 0.08 |
|  |  |  |  |  |  |  | Bacteriuria | 1.13 (0.85-1.47) | MRR (CI) | 0.34 |
|  |  |  |  |  |  |  | Blood pressure | 0.96 (0.92-1.01) | MRR (CI) | 0.15 |
|  |  |  |  |  |  |  | ECG abnormalities | 1.26 (0.97-1.01) | MRR (CI) | 0.08 |
|  |  |  |  |  |  |  | Haematocrit | 0.86 (1.51-2.54) | MRR (CI) | 0.02 |
|  |  |  |  |  |  |  | Mobility impairment | 1.96 (1.52-4.45) | MRR (CI) | 0.001 |
|  |  |  |  |  |  |  | Serum cholesterol | 0.93 (0.85-1.01) | MRR (CI) | 0.9 |
|  |  |  |  |  |  |  | Sex (female ref) | 1.25 (0.89-1.74) | MRR (CI) | 0.2 |
|  |  |  |  |  |  |  | Smoking - non-smoker (ref) vs smoker | 1.63 (1.14-2.32) | MRR (CI) | 0.01 |
|  |  |  |  |  |  |  | Socioeconomic status- not paying fees (ref) vs paying fees | 1.00 (0.77-1.31) | MRR (CI) | 0.98 |
|  |  |  |  |  |  |  | Weight - 5kn increments | 1.01 (0.95-1.07) | MRR (CI) | 0.83 |
| Engle and Graney, 1993  USA  Prospective longitudinal study    QAS: 11 | n=647 | Newly admitted residents aged 60 and over who were medically stable | 78.4 (10.6)    74.6% female | Nursing homes, n=8 | Baseline data collection over one year, year not stated, follow up for three months and six months, data collected from resident interview, caretaker interview and chart review.  Analysis: Multiple logistic regression | NR | **Cohort 1: Three months** |  |  |  |
|  |  |  |  |  |  |  | ADL - feeding (Eight Scaled Outcome Criteria) | NR | OR (CI) | NS |
|  |  |  |  |  |  |  | ADL - defecation (Eight Scaled Outcome Criteria) | NR | OR (CI) | NS |
|  |  |  |  |  |  |  | ADL - dressing (Eight Scaled Outcome Criteria) | NR | OR (CI) | NS |
|  |  |  |  |  |  |  | ADL - grooming (Eight Scaled Outcome Criteria) | NR | OR (CI) | NS |
|  |  |  |  |  |  |  | ADL - hygiene (Eight Scaled Outcome Criteria) (lower score= more dependence) | NR | OR (CI) | NS |
|  |  |  |  |  |  |  | ADL - transferring | NR | OR (CI) | NS |
|  |  |  |  |  |  |  | Admission from the community | NR | OR (CI) | NS |
|  |  |  |  |  |  |  | Age | NR | OR (CI) | NS |
|  |  |  |  |  |  |  | Cancer | 8.07 (3.91-16.65) | OR (CI) | NR |
|  |  |  |  |  |  |  | Dementia | NR | OR (CI) | NS |
|  |  |  |  |  |  |  | Education low (ref) vs high | 1.51 (1.18-1.93) | OR (CI) | NR |
|  |  |  |  |  |  |  | Ethnicity | NR | OR (CI) | NS |
|  |  |  |  |  |  |  | Hip fracture | NR | OR (CI) | NS |
|  |  |  |  |  |  |  | Level of care - Intermediate (ref) vs skilled | NR | OR (CI) | NS |
|  |  |  |  |  |  |  | Mental status (SPMSQ) (memory, orientation etc.) | NR | OR (CI) | NS |
|  |  |  |  |  |  |  | Number of children living within one mile of the care home | NR | OR (CI) | NS |
|  |  |  |  |  |  |  | Number of diagnoses | NR | OR (CI) | NS |
|  |  |  |  |  |  |  | Number of medications | NR | OR (CI) | NS |
|  |  |  |  |  |  |  | Poor ambulation | 2.14 (1.25-3.66) | OR (CI) | NR |
|  |  |  |  |  |  |  | Poor urination | 2.32 (1.3-4.14) | OR (CI) | NR |
|  |  |  |  |  |  |  | Readmission to the nursing home | NR | OR (CI) | NS |
|  |  |  |  |  |  |  | Sex | NR | OR (CI) | NS |
|  |  |  |  |  |  |  | Type of nursing home | NR | OR (CI) | NS |
|  |  |  |  |  |  |  | Type of payment for care | NR | OR (CI) | NS |
|  |  |  |  |  |  |  | **Cohort 2: Six months** |  |  |  |
|  |  |  |  |  |  |  | ADL - feeding (Eight Scaled Outcome Criteria) | NR | OR (CI) | NS |
|  |  |  |  |  |  |  | ADL - defecation (Eight Scaled Outcome Criteria) | NR | OR (CI) | NS |
|  |  |  |  |  |  |  | ADL - dressing (Eight Scaled Outcome Criteria) | NR | OR (CI) | NS |
|  |  |  |  |  |  |  | ADL - grooming (Eight Scaled Outcome Criteria) | NR | OR (CI) | NS |
|  |  |  |  |  |  |  | ADL - hygiene (Eight Scaled Outcome Criteria) (lower score= more dependence) | NR | OR (CI) | NS |
|  |  |  |  |  |  |  | ADL - transferring | 4.33 (2.31-8.11) | OR (CI) | NR |
|  |  |  |  |  |  |  | Admission from the community | NR | OR (CI) | NS |
|  |  |  |  |  |  |  | Age | 1.06 (1.01 -1.12) | OR (CI) | NR |
|  |  |  |  |  |  |  | Cancer - | 10.77 (5.17 -22.44) | OR (CI) | NR |
|  |  |  |  |  |  |  | Dementia | NR | OR (CI) | NS |
|  |  |  |  |  |  |  | Education (Low) | 1.31 (1.05 -1.63) | OR (CI) | NR |
|  |  |  |  |  |  |  | Ethnicity | NR | OR (CI) | NS |
|  |  |  |  |  |  |  | Hip fracture | NR | OR (CI) | NS |
|  |  |  |  |  |  |  | Level of care - Intermediate (ref) vs skilled | 2.83 (1.8 - 4.47) | OR (CI) | NR |
|  |  |  |  |  |  |  | Marital status - Other (ref) vs Married | 2.68 (1.66-4.34) | OR (CI) | NR |
|  |  |  |  |  |  |  | Mental status (SPMSQ) (memory, orientation etc.) | NR | OR (CI) | NS |
|  |  |  |  |  |  |  | Number of children living within one mile of the care home | NR | OR (CI) | NS |
|  |  |  |  |  |  |  | Number of diagnoses | NR | OR (CI) | NS |
|  |  |  |  |  |  |  | Number of medications | 1.44 (1.12-1.84) | OR (CI) | NR |
|  |  |  |  |  |  |  | Poor ambulation | NR | OR (CI) | NS |
|  |  |  |  |  |  |  | Poor urination | NR | OR (CI) | NS |
|  |  |  |  |  |  |  | Readmission to the nursing home | NR | OR (CI) | NS |
|  |  |  |  |  |  |  | Sex | NR | OR (CI) | NS |
|  |  |  |  |  |  |  | Type of nursing home | NR | OR (CI) | NS |
|  |  |  |  |  |  |  | Type of payment for care | NR | OR (CI) | NS |
| Fernandez and Lapane, 2002 (13)  USA  Follow up study    QAS: 10 | n=15,237 | Residents with PD | NR    59.6% female | Nursing homes, n=1,492 | Baseline data collection 1992-1996, follow up for three years, data collected using the MDS.    Analysis: Cox Proportional Hazard Models | 50% residents died within three years | ADL Score - mild (ref) vs moderate | 1.29 (1.12-1.49) | RR (CI) | NR |
|  |  |  |  |  |  |  | ADL Score - mild (ref) vs severe | 1.81 (1.53-2.13) | RR (CI) | NR |
|  |  |  |  |  |  |  | Age - 65-74 (ref) vs 75-84 | 1.37 (1.24-1.51) | RR (CI) | NR |
|  |  |  |  |  |  |  | Age - 65-74 (ref) vs 85+ | 2.22 (1.99-2.47) | RR (CI) | NR |
|  |  |  |  |  |  |  | Alzheimer’s disease | 0.89 (0.79-1.00) | RR (CI) | NR |
|  |  |  |  |  |  |  | Anxiety | 0.86 (0.73-1.01) | RR (CI) | NR |
|  |  |  |  |  |  |  | Arteriosclerotic heart disease | 1.06 (0.97-1.15) | RR (CI) | NR |
|  |  |  |  |  |  |  | Arthritis | 1.05 (0.96-1.14) | RR (CI) | NR |
|  |  |  |  |  |  |  | Aspiration | 1.58 (0.97-2.56) | RR (CI) | NR |
|  |  |  |  |  |  |  | Balance problems | 1.10 (1.01-1.21) | RR (CI) | NR |
|  |  |  |  |  |  |  | Bladder incontinence | 1.16 (1.04-1.29) | RR (CI) | NR |
|  |  |  |  |  |  |  | Bowel incontinence | 1.06 (0.59-1.18) | RR (CI) | NR |
|  |  |  |  |  |  |  | Chronic obstructive pulmonary disease | 1.35 (1.19-1.54) | RR (CI) | NR |
|  |  |  |  |  |  |  | Cognitive Impairment - mild (ref) vs moderate | 1.28 (1.17-1.40) | RR (CI) | NR |
|  |  |  |  |  |  |  | Cognitive Impairment - mild (ref) vs severe | 1.54 (1.38-1.72) | RR (CI) | NR |
|  |  |  |  |  |  |  | Congestive heart failure | 1.49 (1.35-1.65) | RR (CI) | NR |
|  |  |  |  |  |  |  | Constipation | 0.90 (0.81-1.0) | RR (CI) | NR |
|  |  |  |  |  |  |  | Depression | 0.91 (0.82-1.01) | RR (CI) | NR |
|  |  |  |  |  |  |  | Diabetes | 1.22 (1.11-1.35) | RR (CI) | NR |
|  |  |  |  |  |  |  | Ethnicity - White, non-Hispanic (ref) - African American | 0.74 (0.62-0.87) | RR (CI) | NR |
|  |  |  |  |  |  |  | Ethnicity - White, non-Hispanic (ref) - Other minority | 0.73 (0.61-0.87) | RR (CI) | NR |
|  |  |  |  |  |  |  | Fractures | 0.81 (0.64-1.07) | RR (CI) | NR |
|  |  |  |  |  |  |  | Hallucinations | 0.74 (0.77-1.15) | RR (CI) | NR |
|  |  |  |  |  |  |  | Hearing impairment | 1.15 (0.99-1.33) | RR (CI) | NR |
|  |  |  |  |  |  |  | Hypertension | 0.96 (0.89-1.04) | RR (CI) | NR |
|  |  |  |  |  |  |  | Peripheral vascular disease | 1.11 (0.96-1.28) | RR (CI) | NR |
|  |  |  |  |  |  |  | Physically abusive | 1.02 (0.87-1.20) | RR (CI) | NR |
|  |  |  |  |  |  |  | Pneumonia | 1.39 (1.09-1.77) | RR (CI) | NR |
|  |  |  |  |  |  |  | Pressure ulcers | 1.25 (1.14-1.37) | RR (CI) | NR |
|  |  |  |  |  |  |  | Sex (male ref) | 0.58 (0.54-0.62) | RR (CI) | NR |
|  |  |  |  |  |  |  | Speech impairment | 1.18 (1.06-1.31) | RR (CI) | NR |
|  |  |  |  |  |  |  | Urinary tract infection | 0.99 (0.88-1.10) | RR (CI) | NR |
|  |  |  |  |  |  |  | Verbally abusive | 0.90 (0.78-1.05) | RR (CI) | NR |
|  |  |  |  |  |  |  | Vision problems | 1.38 (1.20-1.57) | RR (CI) | NR |
|  |  |  |  |  |  |  | Wandering | 0.97 (0.88-1.12) | RR (CI) | NR |
| Flacker and Kiely, 1998 (14)  USA  Retrospective cohort study    QAS: 8 | n=780 | Residents residing in the LTCF for at least one year | 88.3 (6.4)  75.9% female | LTCFs, n=1 | Baseline data collection 1994 to 1997, follow up for one year, data collected using MDS.  Analysis: Proportional Hazards Regression Model | 20.4% residents died within one year | Activities - shopping trips | NR | RR (CI) | NS |
|  |  |  |  |  |  |  | Activities - spiritual involvement | NR | RR (CI) | NS |
|  |  |  |  |  |  |  | Activities - outdoor walking/wheeling | NR | RR (CI) | NS |
|  |  |  |  |  |  |  | Age >88 years | 1.48 (1.07-2.05) | RR (CI) | 0.019 |
|  |  |  |  |  |  |  | Anaemia | NR | RR (CI) | NS |
|  |  |  |  |  |  |  | Arthritis | NR | RR (CI) | NS |
|  |  |  |  |  |  |  | Behavioural problems increase past 90 days | NR | RR (CI) | NS |
|  |  |  |  |  |  |  | Body mass index <=22 | 1.75 (1.26-2.43) | RR (CI) | <0.001 |
|  |  |  |  |  |  |  | Bowel incontinence | NR | RR (CI) | NS |
|  |  |  |  |  |  |  | Care needs increase past 90 days | NR | RR (CI) | NS |
|  |  |  |  |  |  |  | Chewing problems | NR | RR (CI) | NS |
|  |  |  |  |  |  |  | Chronic obstructive pulmonary disease | NR | RR (CI) | NS |
|  |  |  |  |  |  |  | Cognitive decline past 90 days | NR | RR (CI) | NS |
|  |  |  |  |  |  |  | Cognitive functioning (CPS) | NR | RR (CI) | NS |
|  |  |  |  |  |  |  | Congestive heart failure | 1.57 (1.01-2.25) | RR (CI) | 0.014 |
|  |  |  |  |  |  |  | Dementia | NR | RR (CI) | NS |
|  |  |  |  |  |  |  | Diabetes | NR | RR (CI) | NS |
|  |  |  |  |  |  |  | Diuretic medication use | NR | RR (CI) | NS |
|  |  |  |  |  |  |  | Fall in past 180 days | NR | RR (CI) | NS |
|  |  |  |  |  |  |  | Functional ability (higher numbers = higher impairment) | 2.50 (1.73-3.60) | RR (CI) | <0.001 |
|  |  |  |  |  |  |  | Hearing problem | NR | RR (CI) | NS |
|  |  |  |  |  |  |  | Impaired decision making | NR | RR (CI) | NS |
|  |  |  |  |  |  |  | Mechanical diet | NR | RR (CI) | NS |
|  |  |  |  |  |  |  | Pain | NR | RR (CI) | NS |
|  |  |  |  |  |  |  | Pain medication use | NR | RR (CI) | NS |
|  |  |  |  |  |  |  | Persistent abnormal mood | NR | RR (CI) | NS |
|  |  |  |  |  |  |  | Pressure ulcer | NR | RR (CI) | NS |
|  |  |  |  |  |  |  | Problem making self-understood | NR | RR (CI) | NS |
|  |  |  |  |  |  |  | Problem understanding others | NR | RR (CI) | NS |
|  |  |  |  |  |  |  | Reduced activity time | NR | RR (CI) | NS |
|  |  |  |  |  |  |  | Resists care | NR | RR (CI) | NS |
|  |  |  |  |  |  |  | Restraint use - bed rail | NR | RR (CI) | NS |
|  |  |  |  |  |  |  | Restraint use - trunk | NR | RR (CI) | NS |
|  |  |  |  |  |  |  | Sex (female ref) | 1.76 (1.24-2.50) | RR (CI) | 0.001 |
|  |  |  |  |  |  |  | Short term memory impairment | NR | RR (CI) | NS |
|  |  |  |  |  |  |  | Shortness of breath | 2.08 (1.26-3.43 | RR (CI) | 0.004 |
|  |  |  |  |  |  |  | Socially inappropriate behaviour | NR | RR (CI) | NS |
|  |  |  |  |  |  |  | Stroke | NR | RR (CI) | NS |
|  |  |  |  |  |  |  | Swallowing problems | 1.81 (1.1 8-2.78) | RR (CI) | 0.006 |
|  |  |  |  |  |  |  | Unstable conditions (conditions which make cognition, ADLs or behaviour unstable) | NR | RR (CI) | NS |
|  |  |  |  |  |  |  | Urine infection | NR | RR (CI) | NS |
|  |  |  |  |  |  |  | Vision impairment | NR | RR (CI) | NS |
|  |  |  |  |  |  |  | Wandering | NR | RR (CI) | NS |
|  |  |  |  |  |  |  | Weight loss past 180 days | 2.26 (1 563.28) | RR (CI) | <0.001 |
| Flacker and Kiely, 2003 (15)  USA  Retrospective cohort study    QAS: 9 | n=60,341 | Residents aged 65 and over | NR    69.0% female | Nursing homes, n= NR | Baseline data collection 1994 to 1997, follow up for one year, data collected using MDS.    Proportional Hazards Regression Analysis | 32.1% residents died within one year | **Cohort 1 Long stay residents (in nursing home over 1 year)** |  |  |  |
|  |  |  |  |  |  |  | Activities - Whether a resident prefers spiritual/religious activity | NR | HR (CI) | NS |
|  |  |  |  |  |  |  | Activities - Whether they are usually sleeping | NR | HR (CI) | NS |
|  |  |  |  |  |  |  | Age >=84 | 1.24 (1.16–1.32) | HR (CI) | NR |
|  |  |  |  |  |  |  | Antianxiety medications | NR | HR (CI) | NS |
|  |  |  |  |  |  |  | Antidepressant medications | NR | HR (CI) | NS |
|  |  |  |  |  |  |  | Antipsychotic medications | NR | HR (CI) | NS |
|  |  |  |  |  |  |  | Balance problems | NR | HR (CI) | NS |
|  |  |  |  |  |  |  | Bed rail | NR | HR (CI) | NS |
|  |  |  |  |  |  |  | Bedfast | NR | HR (CI) | NS |
|  |  |  |  |  |  |  | Body mass index <23 kg/m2 | 1.47 (1.38–1.57) | HR (CI) | NR |
|  |  |  |  |  |  |  | Bowel incontinence | NR | HR (CI) | NS |
|  |  |  |  |  |  |  | Cancer | NR | HR (CI) | NS |
|  |  |  |  |  |  |  | Chewing problem | NR | HR (CI) | NS |
|  |  |  |  |  |  |  | Cognitive functioning (CPS) | NR | HR (CI) | NS |
|  |  |  |  |  |  |  | Congestive heart failure | 1.58 (1.48–1.69) | HR (CI) | NR |
|  |  |  |  |  |  |  | Dehydration | NR | HR (CI) | NS |
|  |  |  |  |  |  |  | Delirium | NR | HR (CI) | NS |
|  |  |  |  |  |  |  | Diabetes | 1.32 (1.22–1.42) | HR (CI) | NR |
|  |  |  |  |  |  |  | Exacerbation of chronic condition | NR | HR (CI) | NS |
|  |  |  |  |  |  |  | Fall in past 30 days | NR | HR (CI) | NS |
|  |  |  |  |  |  |  | Fall in past 31 to 180 days | NR | HR (CI) | NS |
|  |  |  |  |  |  |  | Feeding tube | 2.09 (1.73–2.51) | HR (CI) | NR |
|  |  |  |  |  |  |  | Fever | NR | HR (CI) | NS |
|  |  |  |  |  |  |  | Functional ability - low score | 1.99 (1.74–2.27) | HR (CI) | NR |
|  |  |  |  |  |  |  | Hearing problem | NR | HR (CI) | NS |
|  |  |  |  |  |  |  | Intravenous fluids | NR | HR (CI) | NS |
|  |  |  |  |  |  |  | Intravenous medications | NR | HR (CI) | NS |
|  |  |  |  |  |  |  | Less than 25% of food uneaten | 1.86 (1.64–2.11) | HR (CI) | NR |
|  |  |  |  |  |  |  | Long term memory problems | NR | HR (CI) | NS |
|  |  |  |  |  |  |  | Mechanically altered diet | NR | HR (CI) | NS |
|  |  |  |  |  |  |  | More than >5% of food uneaten | NR | HR (CI) | NS |
|  |  |  |  |  |  |  | New medication in past 90 days | NR | HR (CI) | NS |
|  |  |  |  |  |  |  | Orientation problem | NR | HR (CI) | NS |
|  |  |  |  |  |  |  | Pain - frequent | NR | HR (CI) | NS |
|  |  |  |  |  |  |  | Physically abusive behaviour | NR | HR (CI) | NS |
|  |  |  |  |  |  |  | Pressure ulcers | NR | HR (CI) | NS |
|  |  |  |  |  |  |  | Problem making oneself understood | NR | HR (CI) | NS |
|  |  |  |  |  |  |  | Problem with decision-making | NR | HR (CI) | NS |
|  |  |  |  |  |  |  | Recent changes behaviour | NR | HR (CI) | NS |
|  |  |  |  |  |  |  | Recent changes cognition | NR | HR (CI) | NS |
|  |  |  |  |  |  |  | Recent changes communication | NR | HR (CI) | NS |
|  |  |  |  |  |  |  | Recent changes urinary continence | NR | HR (CI) | NS |
|  |  |  |  |  |  |  | Recent decline in function | NR | HR (CI) | NS |
|  |  |  |  |  |  |  | Refuses fluids | NR | HR (CI) | NS |
|  |  |  |  |  |  |  | Sex (female ref) | 1.59 (1.49–1.70) | HR (CI) | NR |
|  |  |  |  |  |  |  | Short term memory problems | NR | HR (CI) | NS |
|  |  |  |  |  |  |  | Shortness of breath | 2.69 (2.20–3.29) | HR (CI) | NR |
|  |  |  |  |  |  |  | Social engagement (SCS) | NR | HR (CI) | NS |
|  |  |  |  |  |  |  | Socially inappropriate behaviour | NR | HR (CI) | NS |
|  |  |  |  |  |  |  | Swallowing problem | NR | HR (CI) | NS |
|  |  |  |  |  |  |  | Therapeutic diet | NR | HR (CI) | NS |
|  |  |  |  |  |  |  | Unstable conditions (conditions which make cognition, ADLs or behaviour unstable ) | 2.16 (1.86–2.50) | HR (CI) | NR |
|  |  |  |  |  |  |  | Urinary catheter | NR | HR (CI) | NS |
|  |  |  |  |  |  |  | Verbally abusive behaviour | NR | HR (CI) | NS |
|  |  |  |  |  |  |  | Vision problem | NR | HR (CI) | NS |
|  |  |  |  |  |  |  | Wandering | NR | HR (CI) | NS |
|  |  |  |  |  |  |  | Weight loss | 2.04 (1.72–2.34) | HR (CI) | NR |
|  |  |  |  |  |  |  | **Cohort 2 Newly admitted residents** |  |  |  |
|  |  |  |  |  |  |  | Activities - Whether a resident prefers spiritual/religious activity | NR | HR (CI) | NS |
|  |  |  |  |  |  |  | Activities - Whether they are usually sleeping | NR | HR (CI) | NS |
|  |  |  |  |  |  |  | Age >=84 | NR | HR (CI) | NS |
|  |  |  |  |  |  |  | Antianxiety medications | NR | HR (CI) | NS |
|  |  |  |  |  |  |  | Antidepressant medications | NR | HR (CI) | NS |
|  |  |  |  |  |  |  | Antipsychotic medications | NR | HR (CI) | NS |
|  |  |  |  |  |  |  | Balance problems | NR | HR (CI) | NS |
|  |  |  |  |  |  |  | Bed rail | NR | HR (CI) | NS |
|  |  |  |  |  |  |  | Bedfast | 1.92 (1.75–2.10) | HR (CI) | NR |
|  |  |  |  |  |  |  | Body mass index less than 23 kg/m sq | 1.29 (1.25–1.34) | HR (CI) | NR |
|  |  |  |  |  |  |  | Bowel incontinence | 1.39 (1.32–1.48) | HR (CI) | NR |
|  |  |  |  |  |  |  | Cancer | 2.48 (2.34–2.63) | HR (CI) | NR |
|  |  |  |  |  |  |  | Chewing problem | NR | HR (CI) | NS |
|  |  |  |  |  |  |  | Cognitive functioning (CPS) | NR | HR (CI) | NS |
|  |  |  |  |  |  |  | Congestive heart failure | 1.65 (1.60–1.71) | HR (CI) | NR |
|  |  |  |  |  |  |  | Dehydration | NR | HR (CI) | NS |
|  |  |  |  |  |  |  | Delirium | NR | HR (CI) | NS |
|  |  |  |  |  |  |  | Diabetes | NR | HR (CI) | NS |
|  |  |  |  |  |  |  | Exacerbation of chronic condition | NR | HR (CI) | NS |
|  |  |  |  |  |  |  | Fall in past 30 days | NR | HR (CI) | NS |
|  |  |  |  |  |  |  | Fall in past 31 to 180 days | NR | HR (CI) | NS |
|  |  |  |  |  |  |  | Feeding tube | NR | HR (CI) | NS |
|  |  |  |  |  |  |  | Fever | NR | HR (CI) | NS |
|  |  |  |  |  |  |  | Functional ability = Low) | 1.76 (1.66–1.87) | HR (CI) | NR |
|  |  |  |  |  |  |  | Hearing problem | NR | HR (CI) | NS |
|  |  |  |  |  |  |  | Intravenous fluids | NR | HR (CI) | NS |
|  |  |  |  |  |  |  | Intravenous medications | NR | HR (CI) | NS |
|  |  |  |  |  |  |  | Less than 25% of food uneaten | 1.80 (1.71–1.89) | HR (CI) | NR |
|  |  |  |  |  |  |  | Long term memory problems | NR | HR (CI) | NS |
|  |  |  |  |  |  |  | Mechanically altered diet | NR | HR (CI) | NS |
|  |  |  |  |  |  |  | More than >5% of food uneaten | NR | HR (CI) | NS |
|  |  |  |  |  |  |  | New medication in past 90 days | NR | HR (CI) | NS |
|  |  |  |  |  |  |  | Orientation problem | NR | HR (CI) | NS |
|  |  |  |  |  |  |  | Pain - frequent | NR | HR (CI) | NS |
|  |  |  |  |  |  |  | Physically abusive behaviour | NR | HR (CI) | NS |
|  |  |  |  |  |  |  | Pressure ulcers | NR | HR (CI) | NS |
|  |  |  |  |  |  |  | Problem making oneself understood | NR | HR (CI) | NS |
|  |  |  |  |  |  |  | Problem with decision-making | NR | HR (CI) | NS |
|  |  |  |  |  |  |  | Recent changes behaviour | NR | HR (CI) | NS |
|  |  |  |  |  |  |  | Recent changes cognition | NR | HR (CI) | NS |
|  |  |  |  |  |  |  | Recent changes communication | NR | HR (CI) | NS |
|  |  |  |  |  |  |  | Recent changes urinary continence | NR | HR (CI) | NS |
|  |  |  |  |  |  |  | Recent decline in function | NR | HR (CI) | NS |
|  |  |  |  |  |  |  | Refuses fluids | NR | HR (CI) | NS |
|  |  |  |  |  |  |  | Sex (female ref) | 1.52 (1.47–1.57) | HR (CI) | NR |
|  |  |  |  |  |  |  | Short term memory problems | NR | HR (CI) | NS |
|  |  |  |  |  |  |  | Shortness of breath | 2.24 (2.09–2.40) | HR (CI) | NR |
|  |  |  |  |  |  |  | Social engagement (SCS) | NR | HR (CI) | NS |
|  |  |  |  |  |  |  | Socially inappropriate behaviour | NR | HR (CI) | NS |
|  |  |  |  |  |  |  | Swallowing problem | 1.53 (1.43–1.64) | HR (CI) | NR |
|  |  |  |  |  |  |  | Therapeutic diet | NR | HR (CI) | NS |
|  |  |  |  |  |  |  | Unstable conditions (conditions which make cognition, ADLs or behaviour unstable ) | 1.87 (1.76–1.98) | HR (CI) | NR |
|  |  |  |  |  |  |  | Urinary catheter | NR | HR (CI) | NS |
|  |  |  |  |  |  |  | Verbally abusive behaviour | NR | HR (CI) | NS |
|  |  |  |  |  |  |  | Vision problem | NR | HR (CI) | NS |
|  |  |  |  |  |  |  | Wandering | NR | HR (CI) | NS |
|  |  |  |  |  |  |  | Weight loss | NR | HR (CI) | NS |
| Foebel et al, 2013 (16)  Canada  Prospective  cohort study    QAS: 11 | n=546 | Newly admitted residents aged 65 and over | 84.2 (6.6)    67% female | LTCFs, n=42 | Baseline data collection 2004 to 2006, follow up for one year, data collected from care home staff and medical records.    Analysis: Cox Proportional Hazards Regression model | 24% residents died within one year | Admission source | NR | HR (CI) | NS |
|  |  |  |  |  |  |  | Age | 1.02 (0.98-1.07) | HR (CI) | 0.31 |
|  |  |  |  |  |  |  | Agitation (CMAI) | NR | HR (CI) | NS |
|  |  |  |  |  |  |  | Arthritis | NR | HR (CI) | NS |
|  |  |  |  |  |  |  | Atrial fibrillation | NR | HR (CI) | NS |
|  |  |  |  |  |  |  | Baseline function and cognition | NR | HR (CI) | NS |
|  |  |  |  |  |  |  | Cancer | NR | HR (CI) | NS |
|  |  |  |  |  |  |  | Cerebrovascular disease | NR | HR (CI) | NS |
|  |  |  |  |  |  |  | Cognitive functioning (MDS Cog) | NR | HR (CI) | NS |
|  |  |  |  |  |  |  | Coronary artery disease | NR | HR (CI) | NS |
|  |  |  |  |  |  |  | Dementia | NR | HR (CI) | NS |
|  |  |  |  |  |  |  | Diabetes | NR | HR (CI) | NS |
|  |  |  |  |  |  |  | Functioning - Barthel Index (lower score=greater dependence) | 0.91 (0.86-0.96) | HR (CI) | 0.0007 |
|  |  |  |  |  |  |  | Heart failure | 3.13 (1.71-5.71) | HR (CI) | 0.0002 |
|  |  |  |  |  |  |  | Hospitalisations or ED visits in the year before admission | NR | HR (CI) | NS |
|  |  |  |  |  |  |  | Hyperlipidaemia | NR | HR (CI) | NS |
|  |  |  |  |  |  |  | Hypertension | NR | HR (CI) | NS |
|  |  |  |  |  |  |  | Left ventricular ejection fraction | NR | HR (CI) | NS |
|  |  |  |  |  |  |  | Mood disorders | NR | HR (CI) | NS |
|  |  |  |  |  |  |  | Neuropsychiatric Inventory | NR | HR (CI) | NS |
|  |  |  |  |  |  |  | Osteoporosis and/or fragility fractures, | NR | HR (CI) | NS |
|  |  |  |  |  |  |  | Peripheral vascular disease | NR | HR (CI) | NS |
|  |  |  |  |  |  |  | Prescribed medications - and minor tranquilizers (benzodiazepines and other sedative hypnotic medications) | NR | HR (CI) | NS |
|  |  |  |  |  |  |  | Prescribed medications - angiotensin receptor blockers | NR | HR (CI) | NS |
|  |  |  |  |  |  |  | Prescribed medications - angiotensin-converting enzyme inhibitors | 1.08 (0.60-1.94) | HR (CI) | 0.8 |
|  |  |  |  |  |  |  | Prescribed medications - antidepressants | NR | HR (CI) | NS |
|  |  |  |  |  |  |  | Prescribed medications - antiplatelet agents | NR | HR (CI) | NS |
|  |  |  |  |  |  |  | Prescribed medications - beta-adrenergic receptor blockers | 0.99 (0.52-1.87) | HR (CI) | 0.98 |
|  |  |  |  |  |  |  | Prescribed medications - calcium channel blockers | NR | HR (CI) | NS |
|  |  |  |  |  |  |  | Prescribed medications - digoxin | NR | HR (CI) | NS |
|  |  |  |  |  |  |  | Prescribed medications - loop diuretics | NR | HR (CI) | NS |
|  |  |  |  |  |  |  | Prescribed medications - major tranquilizers (inc antipsychotics) | 1.99 (1.07-3.71) | HR (CI) | 0.3 |
|  |  |  |  |  |  |  | Prescribed medications - spironolactone | NR | HR (CI) | NS |
|  |  |  |  |  |  |  | Pulmonary disease | 2.41 (1.34-4.34) | HR (CI) | 0.003 |
|  |  |  |  |  |  |  | Pulmonary disease | NR | HR (CI) | NS |
|  |  |  |  |  |  |  | Renal failure | NR | HR (CI) | NS |
|  |  |  |  |  |  |  | Sex (female ref) | 1.15 (0.63-2.08) | HR (CI) | 0.65 |
|  |  |  |  |  |  |  | Smoking - prior exposure | NR | HR (CI) | NS |
|  |  |  |  |  |  |  | Valvular heart disease | NR | HR (CI) | NS |

| Forder and Fernandez, 2011 (17)  UK  Retrospective observational study  QAS: 8 | n= 11,565 | All residents residing in care homes | 85.0 (SD NR)  68.0% female | Care homes, n=305 | Baseline data collection varied, residents who died in 2008-2010, care home records reviewed by the research team.  Analysis: Cox Proportional Hazard Models | 90% residents died within less than six years, median length of stay was 462 days | Age on admission (+1 over mean age) | 103.70% | RHR | < 0.001 |
| --- | --- | --- | --- | --- | --- | --- | --- | --- | --- | --- |
|  |  |  |  |  |  |  | Attendance Allowance uptake rate (+10%) | 100.50% | RHR | 0.049 |
|  |  |  |  |  |  |  | Dementia patient (frail older people reference) | 114.80% | RHR | < 0.001 |
|  |  |  |  |  |  |  | Gender (male reference) | 151.80% | RHR | < 0.001 |
|  |  |  |  |  |  |  | Locality employment ranking (+10%) | 100.70% | RHR | 0.126 |
|  |  |  |  |  |  |  | Locality income ranking (+10%) | 99.10% | RHR | 0.032 |
|  |  |  |  |  |  |  | Nursing bed (residential bed reference) | 109.20% | RHR | 0.001 |

| Formiga, Ferrer and Lopez Soto, 2009 (18)  Spain  Population based study  QAS: 9 | n=49 | Nona-genarian residents in one town | 92.9 (3.0)    89.7% female | Nursing homes, n=NR | Baseline data collection not reported, follow up for three years. Data collection not reported.    Analysis: Cox Multivariate Analysis | 57.1 % residents died within three years | Age | 1.22 (1.09-1.36) | OR (CI) | <0.0001 |
| --- | --- | --- | --- | --- | --- | --- | --- | --- | --- | --- |
|  |  |  |  |  |  |  | Chronic obstructive pulmonary disease | NR | OR (CI) | NS |
|  |  |  |  |  |  |  | Cognitive function (Mini Mental State Examination) | 0.95 (0.91-0.98) | OR (CI) | <0.007 |
|  |  |  |  |  |  |  | Comorbidity (CCI) | NR | OR (CI) | NS |
|  |  |  |  |  |  |  | Diabetes | NR | OR (CI) | NS |
|  |  |  |  |  |  |  | Dyslipidaemia | NR | OR (CI) | NS |
|  |  |  |  |  |  |  | Education | NR | OR (CI) | NS |
|  |  |  |  |  |  |  | Functional status (Barthel Index/Lawton- Brody Index) | NR | OR (CI) | NS |
|  |  |  |  |  |  |  | Heart failure | 4.17 (1.83-9.49) | OR (CI) | <0.001 |
|  |  |  |  |  |  |  | Hypertension | NR | OR (CI) | NS |
|  |  |  |  |  |  |  | Ischemic cardiomyopathy | NR | OR (CI) | NS |
|  |  |  |  |  |  |  | Marital status | NR | OR (CI) | NS |
|  |  |  |  |  |  |  | Number of drugs | NR | OR (CI) | NS |
|  |  |  |  |  |  |  | Previous stroke | NR | OR (CI) | NS |
|  |  |  |  |  |  |  | Sex | NR | OR (CI) | NS |
| Gambassi et al, 1999 (19)  USA  Longitudinal follow up study    QAS: 12 | n=9,264 | Newly admitted residents with AD aged 65 years and over | 82.1 (6.8)    69.2% female | Nursing homes, n=NR | Baseline data collection 1992-1995, follow up until 1997 (5 years), data collected using the SAGE database and the MDS.    Analysis: Cox Proportional Hazards Model | 50% residents died within five years | Age (65-74 ref) 75-84 | 1.34 (1.22-1.48) | RR (CI) | NR |
|  |  |  |  |  |  |  | Age (65-74 ref) 85+ | 1.83 (1.65-2.03) | RR (CI) | NR |
|  |  |  |  |  |  |  | Aphasia | 1.12 (0.89-1.40) | RR (CI) | NR |
|  |  |  |  |  |  |  | Behaviour problems | 0.93 (0.88-1.00) | RR (CI) | NR |
|  |  |  |  |  |  |  | Cardiovascular disease | 1.22 (1.14-1.30) | RR (CI) | NR |
|  |  |  |  |  |  |  | Chronic obstructive pulmonary disease | 1.26 (1.14-1.39) | RR (CI) | NR |
|  |  |  |  |  |  |  | Cognitive Function (CPS) normal (ref) vs moderate impairment | 1.04 (0.91-1.17) | RR (CI) | NR |
|  |  |  |  |  |  |  | Cognitive Function (CPS) normal (ref) vs severe impairment | 1.13 (0.99-1.29) | RR (CI) | NR |
|  |  |  |  |  |  |  | Delirium | 1.17 (1.09-1.27) | RR (CI) | NR |
|  |  |  |  |  |  |  | Depression | 1.11 (1.04-1.18) | RR (CI) | NR |
|  |  |  |  |  |  |  | Diabetes | 1.32 (1.21-1.43) | RR (CI) | NR |
|  |  |  |  |  |  |  | Ethnicity - White (ref) vs African American | 0.82 (0.72-0.94) | RR (CI) | NR |
|  |  |  |  |  |  |  | Ethnicity - White (ref) vs Other minorities | 0.69 (0.57-0.85) | RR (CI) | NR |
|  |  |  |  |  |  |  | Hearing problems | 1.10 (1.00-1.21) | RR (CI) | NR |
|  |  |  |  |  |  |  | History of falls | 1.01 (0.96-1.07) | RR (CI) | NR |
|  |  |  |  |  |  |  | Malnutrition | 1.31 (1.23-1.39) | RR (CI) | NR |
|  |  |  |  |  |  |  | Marital status - Widowed (ref) vs married | 1.04 (0.97-1.12) | RR (CI) | NR |
|  |  |  |  |  |  |  | Marital status - Widowed (ref) vs other | 0.86 (0.75-0.97) | RR (CI) | NR |
|  |  |  |  |  |  |  | Parkinson’s disease | 0.98 (0.86-1.10) | RR (CI) | NR |
|  |  |  |  |  |  |  | Physical function (ADL score) normal (ref) vs need supervision | 1.25 (1.11-1.41) | RR (CI) | NR |
|  |  |  |  |  |  |  | Physical function (ADL score) normal (ref) vs requires assistance | 1.45 (1.27-1.66) | RR (CI) | NR |
|  |  |  |  |  |  |  | Pressure ulcers | 1.24 (1.13-1.36) | RR (CI) | NR |
|  |  |  |  |  |  |  | Restraint use | 1.03 (0.95-1.11) | RR (CI) | NR |
|  |  |  |  |  |  |  | Sex (female ref) | 1.81 (1.70-1.94) | RR (CI) | NR |
|  |  |  |  |  |  |  | Stroke | 1.05 (0.96-1.16) | RR (CI) | NR |
|  |  |  |  |  |  |  | Urinary incontinence | 1.15 (1.06-1.24) | RR (CI) | NR |
|  |  |  |  |  |  |  | Vision problems | 1.13 (1.03-1.23) | RR (CI) | NR |
|  |  |  |  |  |  |  |  |  |  |  |
| Hedinger, Hamming and Bopp, 2015 (20)  Switzerland  Linked observational study    QAS: 10 | n=35739 (11,486 men, 35,739 women) | Residents aged 65 years and over at admission and who had died in 2007 and 2008 | 83.6 (male) / 85.2 (female) (SD NR)  69.2% female | Nursing homes, n=NR | Baseline data collection varied, residents who died in 2007-2008, data collected using three datasets - Swiss National Cohort, Statistics of socio-medical institutions (SOMED) and Medstat- Medical statistics of Swiss hospitals.    Analysis: Negative binomial  regression models | Mean LOS: 790 days (male) / 1250 days (female) | **Men only:** |  |  |  |
|  |  |  |  |  |  |  | Age | 0.96 (0.96–0.97) | IRR (CI) | <0.001 |
|  |  |  |  |  |  |  | Care level - low (ref) vs high | 0.55 (0.52–0.58) | IRR (CI) | <0.001 |
|  |  |  |  |  |  |  | Care level - low (ref) vs medium | 0.71 (0.67–0.75) | IRR (CI) | <0.001 |
|  |  |  |  |  |  |  | Care level - unknown/not specified | 0.30 (0.27–0.33) | IRR (CI) | <0.001 |
|  |  |  |  |  |  |  | Cause of death - cancer (ref) vs COPD | 1.63 (1.46–1.82) | IRR (CI) | <0.001 |
|  |  |  |  |  |  |  | Cause of death - cancer (ref) vs coronary heart disease | 1.75 (1.62–1.88) | IRR (CI) | <0.001 |
|  |  |  |  |  |  |  | Cause of death - cancer (ref) vs Dementia | 1.93 (1.79–2.09) | IRR (CI) | <0.001 |
|  |  |  |  |  |  |  | Cause of death - cancer (ref) vs other | 1.91 (1.80-2.04) | IRR (CI) | <0.001 |
|  |  |  |  |  |  |  | Cause of death - cancer (ref) vs stroke | 2.02 (1.85–2.21) | IRR (CI) | <0.001 |
|  |  |  |  |  |  |  | Children - yes (ref) vs no | 1.16 (1.08–1.24) | IRR (CI) | <0.001 |
|  |  |  |  |  |  |  | Children - yes (ref) vs unknown | 1.16 (1.06–1.27) | IRR (CI) | <0.001 |
|  |  |  |  |  |  |  | Education - medium vs high | 0.98 (0.91–1.05) | IRR (CI) | <0.001 |
|  |  |  |  |  |  |  | Education - medium vs low | 1.17 (1.10–1.23) | IRR (CI) | <0.001 |
|  |  |  |  |  |  |  | Education - medium vs unsure | 1.10 (1.03–1.17) | IRR (CI) | <0.001 |
|  |  |  |  |  |  |  | Home ownership - tenant (ref) vs owner-occupier | 0.67 (0.64–0.71) | IRR (CI) | <0.001 |
|  |  |  |  |  |  |  | Hospitalisation in the 365 days preceding death | 0.36 (0.35-0.38) | IRR (CI) | <0.001 |
|  |  |  |  |  |  |  | Marital status - married (ref) vs divorced | 1.14 (1.03–1.25) | IRR (CI) | <0.001 |
|  |  |  |  |  |  |  | Marital status - married (ref) vs never married | 1.36 (1.24–1.48) | IRR (CI) | <0.001 |
|  |  |  |  |  |  |  | Marital status - married (ref) vs widowed | 1.23 (1.17–1.29) | IRR (CI) | <0.001 |
|  |  |  |  |  |  |  | Multi-morbidity - no (ref) vs unsure | 0.96 (0.89–1.03) | IRR (CI) | <0.001 |
|  |  |  |  |  |  |  | Multi-morbidity - no (ref) vs yes | 1.07 (1.02–1.13) | IRR (CI) | <0.001 |
|  |  |  |  |  |  |  | Nationality - Swiss (ref) vs foreigner | 0.77 (0.69–0.85) | IRR (CI) | <0.001 |
|  |  |  |  |  |  |  | **Women only:** |  |  |  |
|  |  |  |  |  |  |  | Age | 0.96 (0.95–0.96) | IRR (CI) | <0.001 |
|  |  |  |  |  |  |  | Care level - low (ref) vs high | 0.81 (0.78–0.84) | IRR (CI) | <0.001 |
|  |  |  |  |  |  |  | Care level - low (ref) vs medium | 0.86 (0.83–0.89) | IRR (CI) | <0.001 |
|  |  |  |  |  |  |  | Care level - unknown/not specified | 0.44 (0.41–0.48) | IRR (CI) | <0.001 |
|  |  |  |  |  |  |  | Cause of death - cancer (ref) vs COPD | 1.58 (1.44–1.75) | IRR (CI) | <0.001 |
|  |  |  |  |  |  |  | Cause of death - cancer (ref) vs coronary heart disease | 1.89 (1.79–1.99) | IRR (CI) | <0.001 |
|  |  |  |  |  |  |  | Cause of death - cancer (ref) vs Dementia | 1.96 (1.86–2.06) | IRR (CI) | <0.001 |
|  |  |  |  |  |  |  | Cause of death - cancer (ref) vs other | 1.91 (1.83–2.00) | IRR (CI) | <0.001 |
|  |  |  |  |  |  |  | Cause of death - cancer (ref) vs stroke | 1.79 (1.68–1.90) | IRR (CI) | <0.001 |
|  |  |  |  |  |  |  | Children - yes (ref) vs no | 1.10 (1.05–1.14) | IRR (CI) | <0.001 |
|  |  |  |  |  |  |  | Children - yes (ref) vs unknown | 1.22 (1.16–1.28) | IRR (CI) | <0.001 |
|  |  |  |  |  |  |  | Education - medium vs high | 0.96 (0.88–1.05) | IRR (CI) | <0.001 |
|  |  |  |  |  |  |  | Education - medium vs low | 1.09 (1.06–1.13) | IRR (CI) | <0.001 |
|  |  |  |  |  |  |  | Education - medium vs unsure | 0.98 (0.94–1.02) | IRR (CI) | <0.001 |
|  |  |  |  |  |  |  | Home ownership - tenant (ref) vs owner-occupier | 0.65 (0.63–0.67) | IRR (CI) | <0.001 |
|  |  |  |  |  |  |  | Hospitalisation in the 365 days preceding death | 0.42 (0.41–0.43) | IRR (CI) | <0.001 |
|  |  |  |  |  |  |  | Marital status - married (ref) vs divorced | 0.95 (0.89–1.01) | IRR (CI) | <0.001 |
|  |  |  |  |  |  |  | Marital status - married (ref) vs never married | 1.17 (1.10–1.24) | IRR (CI) | <0.001 |
|  |  |  |  |  |  |  | Marital status - married (ref) vs widowed | 1.17 (1.13–1.22) | IRR (CI) | <0.001 |
|  |  |  |  |  |  |  | Multi-morbidity - no (ref) vs unsure | 1.03 (0.99–1.07) | IRR (CI) | <0.05 |
|  |  |  |  |  |  |  | Multi-morbidity - no (ref) vs yes | 0.98 (0.95–1.01) | IRR (CI) | <0.05 |
|  |  |  |  |  |  |  | Nationality - Swiss (ref) vs foreigner | 0.83 (0.76–0.89) | IRR (CI) | <0.001 |
|  |  |  |  |  |  |  | Age | 4% (0.8-7.4%) | % | 0.014 |
| Heppenstall et al, 2015 (21)  New Zealand  Cohort study    QAS: 7 | n=500 (sample randomly selected from 6,289) | Residents across all certified LTCFs | 83 (SD NR)  Gender NR | LTCFs, n=NR | Baseline data collection 2008, follow up for one year, data collected using the Older Persons Ability Level Study.    Analysis: Multivariate logistic regression analysis | 13% residents in rest home care, 26% residents in dementia care, 28% residents in psychogeriatric care and 34% residents in private hospital care died within one year | Needing attention twice or more per night | 2.51 (1.50–4.20) | RR | 0.001 |
|  |  |  |  |  |  |  | Needing help with feeding | 3.07, (1.69–5.59) | RR | 0.002 |
|  |  |  |  |  |  |  | Sex (female ref) | 2.10 (1.22-3.60) | RR | 0.007 |
|  |  |  |  |  |  |  |  |  |  |  |
| Hjaltadootir et al, 1991 (22)  Iceland  Cohort study    QAS: 12 | n=2,206 | Residents who were assessed once at baseline with a MDS within 90 days of admission | 82.5 (7.60)  70% female | Nursing homes, n=NR | Baseline data collection 1996 to 2006, follow up for three years, data collected using the MDS.  Analysis: Weibull Model | 53.1% residents died within three years | Physical functioning (ADL Long Scale) (greater scores = greater dependency) | NR | HR (CI) | <0.001 |
|  |  |  |  |  |  |  | ADL Long Scale 0-3 (ref) vs 10-17 | 1.33 (1.08-1.63) | HR (CI) | 0.007 |
|  |  |  |  |  |  |  | ADL Long Scale 0-3 (ref) vs 18-28 | 1.80 (1.45-2.23) | HR (CI) | <0.001 |
|  |  |  |  |  |  |  | ADL Long Scale 0-3 (ref) vs 4-9 | 1.17 (0.95-1.43) | HR (CI) | 0.134 |
|  |  |  |  |  |  |  | Admission source | NR | HR (CI) | 0.011 |
|  |  |  |  |  |  |  | Admitted from private home, with and without home help (ref) vs acute care hospital/ rehabilitation hospital | 1.27 (1.10-1.47) | HR (CI) | 0.001 |
|  |  |  |  |  |  |  | Admitted from private home, with and without home help (ref) vs board and care/assisted living/group home | 1.11 (0.86-1.45) | HR (CI) | 0.417 |
|  |  |  |  |  |  |  | Admitted from private home, with and without home help (ref) vs nursing home/nursing ward | 1.09 (0.88-1.37) | HR (CI) | 0.408 |
|  |  |  |  |  |  |  | CHESS Score (low score = stable condition) | NR | HR (CI) | 0.079 |
|  |  |  |  |  |  |  | CHESS Score: 0 (ref) vs 1 | 1.18 (0.98-1.42) | HR (CI) | 0.079 |
|  |  |  |  |  |  |  | CHESS Score: 0 (ref) vs 2 | 1.61 (1.35-1.93) | HR (CI) | <0.001 |
|  |  |  |  |  |  |  | CHESS Score: 0 (ref) vs 3 | 2.16 (1.70-2.75) | HR (CI) | <0.001 |
|  |  |  |  |  |  |  | CHESS Score: 0 (ref) vs 4 | 3.95 (3.08-5.07) | HR (CI) | <0.001 |
|  |  |  |  |  |  |  | CHESS Score: 0 (ref) vs 5 | 16.18 (11.41-22.95) | HR (CI) | <0.001 |
|  |  |  |  |  |  |  | Cognitive functioning (CPS) | NR | HR (CI) | NS |
|  |  |  |  |  |  |  | Depression (DRS) | NR | HR (CI) | NS |
|  |  |  |  |  |  |  | ISE Score: (higher score = more social engagement) | NR | HR (CI) | 0.007 |
|  |  |  |  |  |  |  | ISE Score: 6 (ref) vs 0 | 1.63 (1.22-2.19) | HR (CI) | 0.001 |
|  |  |  |  |  |  |  | ISE Score: 6 (ref) vs 3 | 1.32(0.96-1.81) | HR (CI) | 0.092 |
|  |  |  |  |  |  |  | ISE Score: 6 (ref) vs 4 | 1.19 (0.86-1.65) | HR (CI) | 0.303 |
|  |  |  |  |  |  |  | ISE Score: 6 (ref) vs 5 | 1.36 (0.94-1.97) | HR (CI) | 0.102 |
|  |  |  |  |  |  |  | ISE Score: 6 (ref)vs 1 | 1.62 (1.19-2.22) | HR (CI) | 0.002 |
|  |  |  |  |  |  |  | ISE Score: 6 (ref)vs 2 | 1.49 (1.09-2.04) | HR (CI) | 0.013 |
|  |  |  |  |  |  |  | Pain (Pain Scale) | NR | HR (CI) | NS |
| Hui,Wong and Woo, 2004 (23)  Hong Kong  Prospective cohort study    QAS: 10 | n=590/ n=536 | Residents aged 65 years and over | 80.0 (7.9)    59.8% female | Nursing homes, n=4 | Baseline data collection not reported, follow up for two years, data collected by research team.    Analysis: Multivariate Cox regression model | 13.8% residents died within eighteen months | Age | -0.0306 (0.0121) | Coef (SE) | 0.021 |
|  |  |  |  |  |  |  | Cancer | -0.9512 (0.3046) | Coef (SE) | 0.002 |
|  |  |  |  |  |  |  | Clinic visits in the last three months | NR | Coef (SE) | NS |
|  |  |  |  |  |  |  | Education | NR | Coef (SE) | NS |
|  |  |  |  |  |  |  | Functional performance (greater scores=greater dependency) | -0.131 (0.0239) | Coef (SE) | <0.0000 |
|  |  |  |  |  |  |  | Hearing impairment | NR | Coef (SE) | NS |
|  |  |  |  |  |  |  | History of falling in the last three months | NR | Coef (SE) | NS |
|  |  |  |  |  |  |  | Hospitalisations in the last three months | NR | Coef (SE) | NS |
|  |  |  |  |  |  |  | Marital status | NR | Coef (SE) | NS |
|  |  |  |  |  |  |  | Medication use in the last three months | NR | Coef (SE) | NS |
|  |  |  |  |  |  |  | Nutritional status - CAMA <= 1 SD | -0.8417 (0.1957) | Coef (SE) | 0.00002 |
|  |  |  |  |  |  |  | Sex (female ref) | -1.1802 (0.4548) | Coef (SE) | 0.009 |
|  |  |  |  |  |  |  | Vision impairment | NR | Coef (SE) | NS |
| Lapane et al, 2001 (24)  USA  Population cohort    QAS: 12 | n=9,223 | Newly admitted residents with AD aged 65 years and over | 81.5 (7.0) (male)/ 83.3 (7.1) (female)    69.2% female | Nursing homes, n=<1500 | Baseline data collection 1992 to 1995 for four years and ten months, data collected using the Systematic Assessment of Geriatric Drug Use via Epidemiology and MDS    Analysis: Cox Proportional Hazard Models | Death - links with Medicare. | **Men only** |  |  |  |
|  |  |  |  |  |  |  | ADLs - mild limitations (ref) vs dependant | 1.14 (0.91–1.43) | RR (CI) | NR |
|  |  |  |  |  |  |  | ADLs - mild limitations (ref) vs needs supervision | 1.05 (0.86–1.28) | RR (CI) | NR |
|  |  |  |  |  |  |  | Aphasia | 1.00 (0.68–1.49) | RR (CI) | NR |
|  |  |  |  |  |  |  | Behaviour problems | 1.02 (0.92–1.13) | RR (CI) | NR |
|  |  |  |  |  |  |  | Cardiovascular disease | 0.85 (0.77–0.94) | RR (CI) | NR |
|  |  |  |  |  |  |  | Chronic obstructive pulmonary disease | 1.12 (0.98–1.29) | RR (CI) | NR |
|  |  |  |  |  |  |  | Cognitive function (CPS) - minimal (ref) vs moderate impairment | 1.18 (0.95–1.48) | RR (CI) | NR |
|  |  |  |  |  |  |  | Cognitive function (CPS) - minimal (ref) vs severe impairment | 1.53 (1.21–1.94) | RR (CI) | NR |
|  |  |  |  |  |  |  | Delirium | 1.21 (1.07–1.36) | RR (CI) | NR |
|  |  |  |  |  |  |  | Depression | 1.00 (0.90–1.11) | RR (CI) | NR |
|  |  |  |  |  |  |  | Diabetes | 1.39 (1.22–1.58) | RR (CI) | NR |
|  |  |  |  |  |  |  | Ethnicity - White (ref) vs African American | 0.80 (0.65–0.99) | RR (CI) | NR |
|  |  |  |  |  |  |  | Ethnicity - White (ref) vs Other minorities | 1.00 (0.70–1.44) | RR (CI) | NR |
|  |  |  |  |  |  |  | Falls | 1.01 (0.91–1.11) | RR (CI) | NR |
|  |  |  |  |  |  |  | Gait problems | 0.83 (0.73–0.93) | RR (CI) | NR |
|  |  |  |  |  |  |  | Hearing problems | 0.69 (0.60–0.79) | RR (CI) | NR |
|  |  |  |  |  |  |  | Malnutrition (BMI< 21kg/m2) | 1.04 (0.94–1.15) | RR (CI) | NR |
|  |  |  |  |  |  |  | Marital status - married (ref) vs divorced/separated | 1.44 (1.13–1.84) | RR (CI) | NR |
|  |  |  |  |  |  |  | Marital status - married (ref) vs never married | 1.04 (0.83–1.31) | RR (CI) | NR |
|  |  |  |  |  |  |  | Parkinson's disease | 1.24 (1.05–1.46) | RR (CI) | NR |
|  |  |  |  |  |  |  | Pressure ulcers | 1.01 (0.88–1.17) | RR (CI) | NR |
|  |  |  |  |  |  |  | Restrain use | 1.16 (1.03–1.30) | RR (CI) | NR |
|  |  |  |  |  |  |  | Stroke | 1.08 (0.93–1.25) | RR (CI) | NR |
|  |  |  |  |  |  |  | Urinary incontinence | 1.01 (0.88–1.17) | RR (CI) | NR |
|  |  |  |  |  |  |  | Vision problems | 0.98 (0.86–1.13) | RR (CI) | NR |
|  |  |  |  |  |  |  | **Women only** |  |  |  |
|  |  |  |  |  |  |  | ADLs - mild limitations (ref) vs dependant | 1.33 (1.12–1.58) | RR (CI) | NR |
|  |  |  |  |  |  |  | ADLs - mild limitations (ref) vs needs supervision | 1.22 (1.05–1.41) | RR (CI) | NR |
|  |  |  |  |  |  |  | Aphasia | 1.34 (1.01–1.77) | RR (CI) | NR |
|  |  |  |  |  |  |  | Behaviour problems | 1.08 (0.99–1.17) | RR (CI) | NR |
|  |  |  |  |  |  |  | Cardiovascular disease | 0.92 (0.84–0.99) | RR (CI) | NR |
|  |  |  |  |  |  |  | Chronic obstructive pulmonary disease | 1.45 (1.25–1.67) | RR (CI) | NR |
|  |  |  |  |  |  |  | Cognitive function (CPS) - minimal (ref) vs moderate impairment | 0.98 (0.84–1.14) | RR (CI) | NR |
|  |  |  |  |  |  |  | Cognitive function (CPS) - minimal (ref) vs severe impairment | 1.21 (1.02–143) | RR (CI) | NR |
|  |  |  |  |  |  |  | Delirium | 1.09 (0.98–1.20) | RR (CI) | NR |
|  |  |  |  |  |  |  | Depression | 1.09 (1.00–1.18) | RR (CI) | NR |
|  |  |  |  |  |  |  | Diabetes | 1.39 (1.24–1.55) | RR (CI) | NR |
|  |  |  |  |  |  |  | Ethnicity - White (ref) vs African American | 0.89 (0.76–1.06) | RR (CI) | NR |
|  |  |  |  |  |  |  | Ethnicity - White (ref) vs Other minorities | 0.82 (0.65–1.05) | RR (CI) | NR |
|  |  |  |  |  |  |  | Falls | 0.91 (0.84–0.99) | RR (CI) | NR |
|  |  |  |  |  |  |  | Gait problems | 0.93 (0.84–1.02) | RR (CI) | NR |
|  |  |  |  |  |  |  | Hearing problems | 0.71 (0.63–0.80) | RR (CI) | NR |
|  |  |  |  |  |  |  | Malnutrition (BMI< 21kg/m2) | 1.14 (1.06–1.23) | RR (CI) | NR |
|  |  |  |  |  |  |  | Marital status - married (ref) vs divorced/separated | 1.31 (1.09–1.59) | RR (CI) | NR |
|  |  |  |  |  |  |  | Marital status - married (ref) vs never married | 0.90 (0.78–1.05) | RR (CI) | NR |
|  |  |  |  |  |  |  | Parkinson's disease | 1.50 (1.24–1.82) | RR (CI) | NR |
|  |  |  |  |  |  |  | Pressure ulcers | 1.15 (1.02–1.30) | RR (CI) | NR |
|  |  |  |  |  |  |  | Restrain use | 1.18 (1.07–1.31) | RR (CI) | NR |
|  |  |  |  |  |  |  | Stroke | 1.17 (1.03–1.33) | RR (CI) | NR |
|  |  |  |  |  |  |  | Urinary incontinence | 1.10 (0.99–1.21) | RR (CI) | NR |
|  |  |  |  |  |  |  | Vision problems | 0.94 (0.84–1.05) | RR (CI) | NR |
| Lichtenstein, Federspiel and Shaffner, 1985 (25)  USA  Case- control study    QAS: 8 | n=2,049 / matched pairs n=49 | Residents aged 65 years and older newly admitted to the nursing home | Dead:  81.5 (7.5) Survived: 79.6 (7.3)  Matched dead:  81.6 (6.5) Matched survivors:  81.7 (6.8)  Dead:  63% Survived: 79%  Matched dead:  83% Matched survivors:  83% | Nursing homes, n=13 | Baseline data collection 1976 to 1977, follow up for one year, data collected using Tennessee Medicaid Programme medical records.  Analysis: Odds ratios were calculate using techniques for matched data | 1124 dead and 925 survivors within one year | Age | NR | OR (CI) | NS |
|  |  |  |  |  |  |  | Arrhythmias | NR | OR (CI) | NS |
|  |  |  |  |  |  |  | Atherosclerosis | NR | OR (CI) | NS |
|  |  |  |  |  |  |  | Bathes independently or with assistance (ref) vs is bathed by attendants | 8.0 (2.2-47.8) | OR (CI) | NR |
|  |  |  |  |  |  |  | Bladder - continent (ref) vs incontinent | 2.0 (1.1-4.9) | OR (CI) | NR |
|  |  |  |  |  |  |  | Bowels - continent (ref) vs incontinent | 2.6 (1.0 -7.6) | OR (CI) | NR |
|  |  |  |  |  |  |  | Cardiac disease | NR | OR (CI) | NS |
|  |  |  |  |  |  |  | Cerebrovascular disorders | NR | OR (CI) | NS |
|  |  |  |  |  |  |  | Communication of needs - Verbal (ref) vs language barrier or non-communicative | 3.7 (1.5-14.1) | OR (CI) | NR |
|  |  |  |  |  |  |  | Decubitus ulcers | NR | OR (CI) | NS |
|  |  |  |  |  |  |  | Dental impairment | NR | OR (CI) | NS |
|  |  |  |  |  |  |  | Diabetes | NR | OR (CI) | NS |
|  |  |  |  |  |  |  | Dresses independently or with assistance (ref) vs is dressed by attendants or is not dressed | 6.3 (2.2-24.2) | OR (CI) | NR |
|  |  |  |  |  |  |  | Education | NR | OR (CI) | NS |
|  |  |  |  |  |  |  | Ethnicity | NR | OR (CI) | NS |
|  |  |  |  |  |  |  | Feeding - feeds self (ref) vs is fed | 2.4 (1.1-7.0) | OR (CI) | NR |
|  |  |  |  |  |  |  | Fractures - number occurring within the nursing home | NR | OR (CI) | NS |
|  |  |  |  |  |  |  | Genitourinary disorders | NR | OR (CI) | NS |
|  |  |  |  |  |  |  | Hearing impairment | NR | OR (CI) | NS |
|  |  |  |  |  |  |  | Hypertension | NR | OR (CI) | NS |
|  |  |  |  |  |  |  | Marital status | NR | OR (CI) | NS |
|  |  |  |  |  |  |  | Missing limbs | NR | OR (CI) | NS |
|  |  |  |  |  |  |  | Mobile with aid of wheelchair (ref) vs immobile despite wheelchair being available | 4.6 (2.3-12.7) | OR (CI) | NR |
|  |  |  |  |  |  |  | Muscoskeletal disorders | NR | OR (CI) | NS |
|  |  |  |  |  |  |  | Neoplasms | NR | OR (CI) | NS |
|  |  |  |  |  |  |  | Number of children | NR | OR (CI) | NS |
|  |  |  |  |  |  |  | Orientation - orientated (ref) vs disorientated | 2.0 (0.8-7.2) | OR (CI) | NR |
|  |  |  |  |  |  |  | Other diagnoses | NR | OR (CI) | NS |
|  |  |  |  |  |  |  | Previous living arrangements | NR | OR (CI) | NS |
|  |  |  |  |  |  |  | Respiratory disorders | NR | OR (CI) | NS |
|  |  |  |  |  |  |  | Sex | NR | OR (CI) | NS |
|  |  |  |  |  |  |  | Toileting - Uses toilet or commode (ref) vs does not use either | 2.6 (1.0 -7.6) | OR (CI) | NR |
|  |  |  |  |  |  |  | Transferring - transfers self (ref) vs lifted or bedfast | 3.0 (1.3-10.1) | OR (CI) | NR |
|  |  |  |  |  |  |  | Unspecified adjustment reaction | NR | OR (CI) | NS |
|  |  |  |  |  |  |  | Vision impairment | NR | OR (CI) | NS |
|  |  |  |  |  |  |  | Walks independently or with assistance (ref) vs is bedridden or chair fast | 5.0 (1.5-19.7) | OR (CI) | NR |
| Lucchetti et al, 2015 (26)  Brazil  Prospective study    QAS: 8 | n=150 | NR | 76.03 (10.08)  73.3% female | Nursing home, n=1 | Baseline data collection not reported, follow up for five years. Data collection not reported.  Analysis: Logistic Regression | 50% residents died within five years | Age | 1.089 (1.046–1.135) | OR (CI) | < 0.001 |
|  |  |  |  |  |  |  | Anaemia | NR | OR (CI) | NS |
|  |  |  |  |  |  |  | Cancer | NR | OR (CI) | NS |
|  |  |  |  |  |  |  | Cardiovascular diseases | NR | OR (CI) | NS |
|  |  |  |  |  |  |  | Dementia | NR | OR (CI) | NS |
|  |  |  |  |  |  |  | Depression | NR | OR (CI) | NS |
|  |  |  |  |  |  |  | Diabetes | 3.789 (1.266–1.336) | OR (CI) | 0.017 |
|  |  |  |  |  |  |  | Functional dependency | 1.290 (1.100–1.513) | OR (CI) | 0.002 |
|  |  |  |  |  |  |  | Glycated haemoglobin | NR | OR (CI) | NS |
|  |  |  |  |  |  |  | Hyperlipidaemia | 3.207 (1.023–0.060) | OR (CI) | 0.046 |
|  |  |  |  |  |  |  | Hypertension | NR | OR (CI) | NS |
|  |  |  |  |  |  |  | Number of medications | NR | OR (CI) | NS |
|  |  |  |  |  |  |  | Other neurological disorders | NR | OR (CI) | NS |
|  |  |  |  |  |  |  | Sex | NR | OR (CI) | NS |
|  |  |  |  |  |  |  | Stroke | NR | OR (CI) | NS |
|  |  |  |  |  |  |  | Total cholesterol | NR | OR (CI) | NS |
|  |  |  |  |  |  |  | Use of antidiabetic drugs | NR | OR (CI) | NS |
|  |  |  |  |  |  |  | Use of statins | NR | OR (CI) | NS |
| Luk et al, 1993 (27)  Hong Kong  Cohort longitudinal study    QAS: 11 | n=312 | Residents aged 65 years and older with advanced cognitive impairment | 88 (8)  77% female | Residential care homes, n=66 | Baseline data collection October to December 2010, follow up for one year, data collected using community care nurses.    Analysis: Logistic Regression | 37% residents died within one year | Active influenza vaccination | NR | OR (CI) | NS |
|  |  |  |  |  |  |  | Acute and emergency department attendance | NR | OR (CI) | NS |
|  |  |  |  |  |  |  | Acute hospital - admission | NR | OR (CI) | NS |
|  |  |  |  |  |  |  | Acute hospital - length of stay | NR | OR (CI) | NS |
|  |  |  |  |  |  |  | Advance Directive in place | NR | OR (CI) | NS |
|  |  |  |  |  |  |  | Age | NR | OR (CI) | NS |
|  |  |  |  |  |  |  | Bowel incontinence | NR | OR (CI) | NS |
|  |  |  |  |  |  |  | Chronic obstructive pulmonary disease | 3.4 (1.3-8.8) | OR (CI) | 0.011 |
|  |  |  |  |  |  |  | Chronic renal failure | NR | OR (CI) | NS |
|  |  |  |  |  |  |  | Community Care Nursing Services - Enteral feeding tube care | NR | OR (CI) | NS |
|  |  |  |  |  |  |  | Community Care Nursing Services - Injection of medicine service | NR | OR (CI) | NS |
|  |  |  |  |  |  |  | Community Care Nursing Services - Urinary catheter care | NR | OR (CI) | NS |
|  |  |  |  |  |  |  | Community Care Nursing Services - Wound care | NR | OR (CI) | NS |
|  |  |  |  |  |  |  | Contact with Community Geriatric Assessment Team fast-track clinic | NR | OR (CI) | NS |
|  |  |  |  |  |  |  | Contact with Community Visiting Medical Officer consultation | NR | OR (CI) | NS |
|  |  |  |  |  |  |  | Contact with on-site Community Geriatric Assessment Team consultation | NR | OR (CI) | NS |
|  |  |  |  |  |  |  | Convalescence hospital - admission | NR | OR (CI) | NS |
|  |  |  |  |  |  |  | Convalescence hospital - length of stay | NR | OR (CI) | NS |
|  |  |  |  |  |  |  | Depression | NR | OR (CI) | NS |
|  |  |  |  |  |  |  | Diabetes mellitus | NR | OR (CI) | NS |
|  |  |  |  |  |  |  | Dietitian intervention | NR | OR (CI) | NS |
|  |  |  |  |  |  |  | Enteral feeding | 2.0 (1.2-3.4) | OR (CI) | 0.008 |
|  |  |  |  |  |  |  | Functioning - Barthel Index (lower score=greater dependence) | NR | OR (CI) | NS |
|  |  |  |  |  |  |  | Gender | NR | OR (CI) | NS |
|  |  |  |  |  |  |  | Heart failure | NR | OR (CI) | NS |
|  |  |  |  |  |  |  | Hip fracture | NR | OR (CI) | NS |
|  |  |  |  |  |  |  | History of pneumococcal vaccine | 0.47 (0.28-0.78) | OR (CI) | 0.004 |
|  |  |  |  |  |  |  | Hypertension | NR | OR (CI) | NS |
|  |  |  |  |  |  |  | Indwelling urinary catheter use | 3.2 (1.46-7.2) | OR (CI) | 0.004 |
|  |  |  |  |  |  |  | Ischaemic heart disease | NR | OR (CI) | NS |
|  |  |  |  |  |  |  | Marital status | NR | OR (CI) | NS |
|  |  |  |  |  |  |  | Number of diagnosis | NR | OR (CI) | NS |
|  |  |  |  |  |  |  | Number of medications | NR | OR (CI) | NS |
|  |  |  |  |  |  |  | Occupational therapist intervention | NR | OR (CI) | NS |
|  |  |  |  |  |  |  | On Guardianship Order | NR | OR (CI) | NS |
|  |  |  |  |  |  |  | Parkinson’s Disease | NR | OR (CI) | NS |
|  |  |  |  |  |  |  | Physiotherapist intervention | NR | OR (CI) | NS |
|  |  |  |  |  |  |  | Pressure sores (Norton score) | 2.7 (1.37-5.1) | OR (CI) | 0.004 |
|  |  |  |  |  |  |  | Psychogeriatric team consultation | NR | OR (CI) | NS |
|  |  |  |  |  |  |  | Speech therapist assessment | NR | OR (CI) | NS |
|  |  |  |  |  |  |  | Stroke | NR | OR (CI) | NS |
|  |  |  |  |  |  |  | Tracheostomy | NR | OR (CI) | NS |
|  |  |  |  |  |  |  | Urinary incontinence | NR | OR (CI) | NS |
|  |  |  |  |  |  |  | Use of Comprehensive Social Security Assistance | NR | OR (CI) | NS |
|  |  |  |  |  |  |  | Visiting Medical Officer consultation | NR | OR (CI) | NS |
| McCann et al 2009 (28)  Northern Ireland  Prospective longitudinal study  QAS: 12 | n=2,112 | Residents aged 65 and over admitted in the past year at the time of the 2001 census | 83 (7.3)    74% female | Residential, nursing and dual registered care homes, n=257 | Baseline data collection 2001, follow up for five years, data collected using Northern Ireland Mortality Study.  Analysis: Cox Proportional Hazards | 65% residents died within five years | Age > 65–74 (ref) vs 75–84 | 1.50 (1.23 - 1.83) | HR (CI) | < 0.001 |
|  |  |  |  |  |  |  | Age > 65–74 (ref) vs 85–94 | 2.09 (1.72 - 2.54) | HR (CI) | < 0.001 |
|  |  |  |  |  |  |  | Age > 65–74 (ref) vs 95+ | 3.25 (2.39 - 4.41) | HR (CI) | < 0.001 |
|  |  |  |  |  |  |  | Care home type - not in care home (ref) vs dual registered home* | 2.09 (1.81 - 2.40) | HR (CI) | NR |
|  |  |  |  |  |  |  | Care home type - not in care home (ref) vs nursing home* | 2.17 (1.96 - 2.41) | HR (CI) | NR |
|  |  |  |  |  |  |  | Care home type - not in care home (ref) vs residential home* | 1.63 (1.44 - 1.85) | HR (CI) | NR |
|  |  |  |  |  |  |  | General health - good (ref) vs fairly good | 0.96 (0.78 - 1.19) | HR (CI) | < 0.001 |
|  |  |  |  |  |  |  | General health - good (ref) vs not good | 1.29 (1.05 - 1.58) | HR (CI) | < 0.001 |
|  |  |  |  |  |  |  | Marital Status - married (ref) vs single/ widowed / divorced | 0.99 (0.84 - 1.18) | HR (CI) | < 0.001 |
|  |  |  |  |  |  |  | Presence of limiting long term illness | NR | HR (CI) | NR |
|  |  |  |  |  |  |  | Sex (female ref) | 1.34 (1.18 - .53) | HR (CI) | < 0.001 |
| Mehr, Williams and Fries, 1997 (29)  USA  NR    QAS: 11 | n=6,467 / n=5,895 | Newly admitted residents aged 65 and over | NR  66.8% female | Department of Veteran Affairs nursing homes, n=NR | Baseline data collection 1986 to 1987, follow up for six months and one year, data collected using patient records from Veteran Affairs.  Analysis: Cox Proportional Hazards Model | NR | **Cohort 1 - All admissions:** |  |  |  |
|  |  |  |  |  |  |  | ADL Dependency - Least dependant (ref) vs most dependant (RUG II ADL Index) | 6.93 (4.98-9.65) | OR (CI) | NR |
|  |  |  |  |  |  |  | Age Group and Malignancy - Assumes 75-84 age group | NR | OR (CI) | NR |
|  |  |  |  |  |  |  | Alcoholism | NR | OR (CI) | NR |
|  |  |  |  |  |  |  | Anaemia | 1.62 (1.17-2.25) | OR (CI) | NR |
|  |  |  |  |  |  |  | Arthritis | NR | OR (CI) | NR |
|  |  |  |  |  |  |  | Atherosclerotic heart disease | NR | OR (CI) | NR |
|  |  |  |  |  |  |  | Behaviour problems | NR | OR (CI) | NR |
|  |  |  |  |  |  |  | Cerebrovascular disease | NR | OR (CI) | NR |
|  |  |  |  |  |  |  | Congestive heart failure | 2.73 (1.97-3.78) | OR (CI) | NR |
|  |  |  |  |  |  |  | COPD | NR | OR (CI) | NR |
|  |  |  |  |  |  |  | Dementia | NR | OR (CI) | NR |
|  |  |  |  |  |  |  | Diabetes | NR | OR (CI) | NR |
|  |  |  |  |  |  |  | Ethnicity | NR | OR (CI) | NR |
|  |  |  |  |  |  |  | Fluid or electrolyte disorder | NR | OR (CI) | NR |
|  |  |  |  |  |  |  | Gender | NR | OR (CI) | NR |
|  |  |  |  |  |  |  | Hip Fracture | 0.40 (0.21-0.74) | OR (CI) | NR |
|  |  |  |  |  |  |  | Hospitalisation since nursing home | NR | OR (CI) | NR |
|  |  |  |  |  |  |  | Hypertension | NR | OR (CI) | NR |
|  |  |  |  |  |  |  | Infection (except Pneumonia) | NR | OR (CI) | NR |
|  |  |  |  |  |  |  | Kidney disease | NR | OR (CI) | NR |
|  |  |  |  |  |  |  | Major Psychiatric Disorders | NR | OR (CI) | NR |
|  |  |  |  |  |  |  | Malignancy | 2.56 (1.90-3.45) | OR (CI) | NR |
|  |  |  |  |  |  |  | Married | NR | OR (CI) | NR |
|  |  |  |  |  |  |  | Nasogastric feeding | NR | OR (CI) | NR |
|  |  |  |  |  |  |  | Older age - 64-74 group (ref) vs 85+ group | 1.54 (1.15-2.07) | OR (CI) | NR |
|  |  |  |  |  |  |  | Oxygen Use | 2.35 (1.47-3.76) | OR (CI) | NR |
|  |  |  |  |  |  |  | Pneumonia | NR | OR (CI) | NR |
|  |  |  |  |  |  |  | Prior nursing home stay | 0.49 (0.32-0.76 | OR (CI) | NR |
|  |  |  |  |  |  |  | Recent dehydration | NR | OR (CI) | NR |
|  |  |  |  |  |  |  | Recent UTI | NR | OR (CI) | NR |
|  |  |  |  |  |  |  | Rehabilition program | NR | OR (CI) | NR |
|  |  |  |  |  |  |  | Terminally ill | 6.04 (4.19-8.71) | OR (CI) | NR |
|  |  |  |  |  |  |  | **Cohort 2 - Six months survival cohort:** |  | OR (CI) |  |
|  |  |  |  |  |  |  | ADL Dependency - Least dependant (ref) vs most dependant (RUG II ADL Index) | 2.43 (1.73-1.73 | OR (CI) | NR |
|  |  |  |  |  |  |  | Age Group and Malignancy - Assumes 75-84 age group | NR | OR (CI) | NR |
|  |  |  |  |  |  |  | Alcoholism | 0.22 (0.10-0.50) | OR (CI) | NR |
|  |  |  |  |  |  |  | Anaemia | NR | OR (CI) | NR |
|  |  |  |  |  |  |  | Arthritis | 0.71 (0.49-1.03) | OR (CI) | NR |
|  |  |  |  |  |  |  | Atherosclerotic heart disease | NR | OR (CI) | NR |
|  |  |  |  |  |  |  | Behaviour problems | NR | OR (CI) | NR |
|  |  |  |  |  |  |  | Cerebrovascular disease | NR | OR (CI) | NR |
|  |  |  |  |  |  |  | Congestive heart failure | 1.34 (0.97-1.86) | OR (CI) | NR |
|  |  |  |  |  |  |  | COPD | 1.36 (1.01-1.84) | OR (CI) | NR |
|  |  |  |  |  |  |  | Dementia | NR | OR (CI) | NR |
|  |  |  |  |  |  |  | Diabetes | NR | OR (CI) | NR |
|  |  |  |  |  |  |  | Ethnicity | NR | OR (CI) | NR |
|  |  |  |  |  |  |  | Fluid or electrolyte disorder | 1.55 (1.12-2.13) | OR (CI) | NR |
|  |  |  |  |  |  |  | Gender | NR | OR (CI) | NR |
|  |  |  |  |  |  |  | Hip Fracture | NR | OR (CI) | NR |
|  |  |  |  |  |  |  | Hospitalisation since nursing home | 1.60 (1.24-2.07) | OR (CI) | NR |
|  |  |  |  |  |  |  | Hypertension | NR | OR (CI) | NR |
|  |  |  |  |  |  |  | Infection (except Pneumonia) | NR | OR (CI) | NR |
|  |  |  |  |  |  |  | Kidney disease | 2.59 (1.62-4.15) | OR (CI) | NR |
|  |  |  |  |  |  |  | Major Psychiatric Disorders | NR | OR (CI) | NR |
|  |  |  |  |  |  |  | Malignancy | 2.20 (1.50-3.21 | OR (CI) | NR |
|  |  |  |  |  |  |  | Married | NR | OR (CI) | NR |
|  |  |  |  |  |  |  | Nasogastric feeding | NR | OR (CI) | NR |
|  |  |  |  |  |  |  | Older age - 64-74 group (ref) vs 85+ group | 2.11 (1.52-2.92) | OR (CI) | NR |
|  |  |  |  |  |  |  | Oxygen Use | 1.83 (1.07-3.15) | OR (CI) | NR |
|  |  |  |  |  |  |  | Pneumonia | 1.50 (1.12-2.03) | OR (CI) | NR |
|  |  |  |  |  |  |  | Prior nursing home stay | NR | OR (CI) | NR |
|  |  |  |  |  |  |  | Recent dehydration | NR | OR (CI) | NR |
|  |  |  |  |  |  |  | Recent UTI | NR | OR (CI) | NR |
|  |  |  |  |  |  |  | Rehabilition program | NR | OR (CI) | NR |
|  |  |  |  |  |  |  | Terminally ill | 4.82 (2.92-7.96) | OR (CI) | NR |
|  |  |  |  |  |  |  | **Cohort 3 – Twelve months survival cohort:** |  |  |  |
|  |  |  |  |  |  |  | ADL Dependency - Least dependant (ref) vs most dependant (RUG II ADL Index) | 2.64 (1.70 - 4.10) | OR (CI) | NR |
|  |  |  |  |  |  |  | Age Group and Malignancy - Assumes 75-84 age group | NR | OR (CI) | NR |
|  |  |  |  |  |  |  | Alcoholism | NR | OR (CI) | NR |
|  |  |  |  |  |  |  | Anaemia | NR | OR (CI) | NR |
|  |  |  |  |  |  |  | Arthritis | NR | OR (CI) | NR |
|  |  |  |  |  |  |  | Atherosclerotic heart disease | NR | OR (CI) | NR |
|  |  |  |  |  |  |  | Behaviour problems | NR | OR (CI) | NR |
|  |  |  |  |  |  |  | Cerebrovascular disease | NR | OR (CI) | NR |
|  |  |  |  |  |  |  | Congestive heart failure | 2.21 (1.53-3.20) | OR (CI) | NR |
|  |  |  |  |  |  |  | COPD | 1.36 (1.01-1.84) | OR (CI) | NR |
|  |  |  |  |  |  |  | Dementia | NR | OR (CI) | NR |
|  |  |  |  |  |  |  | Diabetes | NR | OR (CI) | NR |
|  |  |  |  |  |  |  | Ethnicity | NR | OR (CI) | NR |
|  |  |  |  |  |  |  | Fluid or electrolyte disorder | NR | OR (CI) | NR |
|  |  |  |  |  |  |  | Gender | NR | OR (CI) | NR |
|  |  |  |  |  |  |  | Hip Fracture | NR | OR (CI) | NR |
|  |  |  |  |  |  |  | Hospitalisation since nursing home | 1.61 (1.14-2.29) | OR (CI) | NR |
|  |  |  |  |  |  |  | Hypertension | NR | OR (CI) | NR |
|  |  |  |  |  |  |  | Infection (except Pneumonia) | NR | OR (CI) | NR |
|  |  |  |  |  |  |  | Kidney disease | NR | OR (CI) | NR |
|  |  |  |  |  |  |  | Major Psychiatric Disorders | NR | OR (CI) | NR |
|  |  |  |  |  |  |  | Malignancy | 2.49 (1.66-3.74) | OR (CI) | NR |
|  |  |  |  |  |  |  | Married | 1.51 (1.09 -2.08) | OR (CI) | NR |
|  |  |  |  |  |  |  | Nasogastric feeding | NR | OR (CI) | NR |
|  |  |  |  |  |  |  | Older age - 64-74 group (ref) vs 85+ group | 2.06 (1.41 - 3.01) | OR (CI) | NR |
|  |  |  |  |  |  |  | Oxygen Use | 2.61 (1.30 -5.21) | OR (CI) | NR |
|  |  |  |  |  |  |  | Pneumonia | 1.74 (1.23-2.46) | OR (CI) | NR |
|  |  |  |  |  |  |  | Prior nursing home stay | 1.92 (1.18-3.13) | OR (CI) | NR |
|  |  |  |  |  |  |  | Recent dehydration | NR | OR (CI) | NR |
|  |  |  |  |  |  |  | Recent UTI | NR | OR (CI) | NR |
|  |  |  |  |  |  |  | Rehabilitation program | NR | OR (CI) | NR |
|  |  |  |  |  |  |  | Terminally ill | 4.31 (2.10 -8.84) | OR (CI) | NR |
| Mitchell et al, 2004 (30)  USA  Retrospective cohort study    QAS: 10 | N=7,014/ n=6,799 | Newly admitted residents aged 68 years and over with advanced dementia | 83 (median)    77% female | Nursing homes, n=643 | Baseline data collection 1994 to 1998, follow up for six months, data collected using MDS.    Analysis: Cox Proportional Hazards model | 28% residents died within six months | ADLs (higher scores = greater dependency) | 1.9 (1.7-2.1) | HR (CI) | NR |
|  |  |  |  |  |  |  | Age | 1.4 (1.3 -1.6) | HR (CI) | NR |
|  |  |  |  |  |  |  | Any fracture in the previous 180 days | NR | HR (CI) | NS |
|  |  |  |  |  |  |  | Aspiration | NR | HR (CI) | NS |
|  |  |  |  |  |  |  | Asthma or emphysema/COPD | NR | HR (CI) | NS |
|  |  |  |  |  |  |  | Bedfast | 1.5 (1.3 -1.7) | HR (CI) | NR |
|  |  |  |  |  |  |  | Body mass index | NR | HR (CI) | NS |
|  |  |  |  |  |  |  | Bowel incontinence | 1.5 (1.3 -1.7) | HR (CI) | NR |
|  |  |  |  |  |  |  | Cancer | 1.7 (1.5-1.9) | HR (CI) | NR |
|  |  |  |  |  |  |  | Cardiac dysrhythmia | NR | HR (CI) | NS |
|  |  |  |  |  |  |  | Chewing or swallowing problem | NR | HR (CI) | NS |
|  |  |  |  |  |  |  | Congestive heart failure | 1.6 (1.4-1.7) | HR (CI) | NR |
|  |  |  |  |  |  |  | Dehydration | NR | HR (CI) | NS |
|  |  |  |  |  |  |  | Diabetes | NR | HR (CI) | NS |
|  |  |  |  |  |  |  | Edema | NR | HR (CI) | NS |
|  |  |  |  |  |  |  | Ethnicity | NR | HR (CI) | NS |
|  |  |  |  |  |  |  | Fever | NR | HR (CI) | NS |
|  |  |  |  |  |  |  | Hallucinations or delusions | NR | HR (CI) | NS |
|  |  |  |  |  |  |  | Insufficient fluid intake | NR | HR (CI) | NS |
|  |  |  |  |  |  |  | Less than 25% of food eaten at most meals | 1.5 (1.3-1.7) | HR (CI) | NR |
|  |  |  |  |  |  |  | Not awake most of day | 1.4 (1.2-1.6) | HR (CI) | NR |
|  |  |  |  |  |  |  | Oxygen therapy in prior 14 days | 1.6 (1.4-1.8) | HR (CI) | NR |
|  |  |  |  |  |  |  | Pneumonia or respiratory tract infection | NR | HR (CI) | NS |
|  |  |  |  |  |  |  | Pressure ulcers | NR | HR (CI) | NS |
|  |  |  |  |  |  |  | Recent weight loss | NR | HR (CI) | NS |
|  |  |  |  |  |  |  | Septicaemia | NR | HR (CI) | NS |
|  |  |  |  |  |  |  | Sex (female ref) | 1.9 (1.7-2.1) | HR (CI) | NR |
|  |  |  |  |  |  |  | Shortness of breath | 1.5 (1.3-1.9) | HR (CI) | NR |
|  |  |  |  |  |  |  | Unstable conditions (conditions which make cognition, ADLs or behaviour unstable ) | 1.5 (1.3 -1.6) | HR (CI) | NR |
|  |  |  |  |  |  |  | Urinary tract infection | NR | HR (CI) | NS |
| Mitchell et al, 2010 (31)  USA  Retrospective cohort study    QAS: 11 | n=  245,132/ n=  218,088 | Residents aged 65 years and over with advanced dementia | 85.4 (7.5)    Gender NR | Nursing homes, n=NR | Baseline data collection 2002, follow up for one year, data collected using MDS.    Analysis: Multivariate logistic regression | 40.6% of residents died within one year | ADL score | 1.42 (1.40-1.44) | OR (CI) | NR |
|  |  |  |  |  |  |  | Age | 1.18 (1.17-1.18) | OR (CI) | NR |
|  |  |  |  |  |  |  | Alzheimer’s disease | NR | OR (CI) | NS |
|  |  |  |  |  |  |  | Anaemia | NR | OR (CI) | NS |
|  |  |  |  |  |  |  | Arteriosclerotic heart disease | NR | OR (CI) | NS |
|  |  |  |  |  |  |  | At least one pressure ulcers >=Stage 2 | 1.44 (1.41-1.46) | OR (CI) | NR |
|  |  |  |  |  |  |  | Bedfast most of day | 1.41 (1.38-1.44) | OR (CI) | NR |
|  |  |  |  |  |  |  | BMI <18.5 kg/m | 1.35 (1.32-1.37) | OR (CI) | NR |
|  |  |  |  |  |  |  | Bowel incontinence | 1.37 (1.34-1.40) | OR (CI) | NR |
|  |  |  |  |  |  |  | Cancer | NR | OR (CI) | NS |
|  |  |  |  |  |  |  | Cardiac dysrhythmias | NR | OR (CI) | NS |
|  |  |  |  |  |  |  | Chewing or swallowing problem | NR | OR (CI) | NS |
|  |  |  |  |  |  |  | Chronic obstructive pulmonary disease | NR | OR (CI) | NS |
|  |  |  |  |  |  |  | Cognitive deterioration in past 90 days | NR | OR (CI) | NS |
|  |  |  |  |  |  |  | Cognitive functioning (CPS) | NR | OR (CI) | NS |
|  |  |  |  |  |  |  | Congestive heart failure | 1.28 (1.26 -1.30) | OR (CI) | NR |
|  |  |  |  |  |  |  | Diabetes | NR | OR (CI) | NS |
|  |  |  |  |  |  |  | Fever in prior seven days | NR | OR (CI) | NS |
|  |  |  |  |  |  |  | Functional deterioration in past 90 days | NR | OR (CI) | NS |
|  |  |  |  |  |  |  | Hallucinations or delusions | NR | OR (CI) | NS |
|  |  |  |  |  |  |  | Hip fracture prior 180 days | NR | OR (CI) | NS |
|  |  |  |  |  |  |  | Hypertension | NR | OR (CI) | NS |
|  |  |  |  |  |  |  | Insufficient oral intake | 1.39 (1.37-1.41) | OR (CI) | NR |
|  |  |  |  |  |  |  | Lethargic or not awake most of the day | NR | OR (CI) | NS |
|  |  |  |  |  |  |  | Other (non-hip) fracture prior 180 days | NR | OR (CI) | NS |
|  |  |  |  |  |  |  | Other infections | NR | OR (CI) | NS |
|  |  |  |  |  |  |  | Parkinson’s disease | NR | OR (CI) | NS |
|  |  |  |  |  |  |  | Peripheral edema | NR | OR (CI) | NS |
|  |  |  |  |  |  |  | Peripheral vascular disease | NR | OR (CI) | NS |
|  |  |  |  |  |  |  | Pneumonia or respiratory tract infection | NR | OR (CI) | NS |
|  |  |  |  |  |  |  | Race | NR | OR (CI) | NS |
|  |  |  |  |  |  |  | Rarely makes oneself understood | NR | OR (CI) | NS |
|  |  |  |  |  |  |  | Recent nursing home admission | 1.72 (1.69-1.75) | OR (CI) | NR |
|  |  |  |  |  |  |  | Recurrent lung aspirations in prior 90 days | NR | OR (CI) | NS |
|  |  |  |  |  |  |  | Renal failure | NR | OR (CI) | NS |
|  |  |  |  |  |  |  | Seizure disorder | NR | OR (CI) | NS |
|  |  |  |  |  |  |  | Sex (female ref) | 1.71 (1.68-1.74) | OR (CI) | NR |
|  |  |  |  |  |  |  | Shortness of breath | 1.57 (1.53-1.61) | OR (CI) | NR |
|  |  |  |  |  |  |  | Stroke | NR | OR (CI) | NS |
|  |  |  |  |  |  |  | Urinary tract infection in prior 30 days | NR | OR (CI) | NS |
|  |  |  |  |  |  |  | Weight loss | 1.30 (1.27-1.33) | OR (CI) | NR |
| Navarro-Gil et al 2014 (32)  Spain  Retrospective longitudinal study    QAS: 9 | n=525    Gender NR | Residents aged 60 and over with Dementia | 85.6 (6.8)  Gender NR | Nursing homes, n=NR | Baseline data collection not reported, follow up for 19 months, data collected using questionnaire completed by resident caregiver within the facility.    Analysis: Multivariate logistic regression | NR | Activities of daily living (EQ-5D) | NR | OR (CI) | NS |
|  |  |  |  |  |  |  | Age - 60–85 years (ref) vs over 85 years | 1.986 (1.229-3.209) | OR (CI) | 0.005 |
|  |  |  |  |  |  |  | Allergy/generalized pruritus | NR | OR (CI) | NS |
|  |  |  |  |  |  |  | Alzheimer’s | NR | OR (CI) | NS |
|  |  |  |  |  |  |  | Anxiety or Depression (EQ-5D) | NR | OR (CI) | NS |
|  |  |  |  |  |  |  | Bone problems | NR | OR (CI) | NS |
|  |  |  |  |  |  |  | Breathing problems | NR | OR (CI) | NS |
|  |  |  |  |  |  |  | Cancer | NR | OR (CI) | NS |
|  |  |  |  |  |  |  | Change in health at 12 months-better/the same (ref) vs worse | 1.653 1.018-2.685 | OR (CI) | 0.042 |
|  |  |  |  |  |  |  | Degree of dementia (CDRS) | NR | OR (CI) | NS |
|  |  |  |  |  |  |  | Depression, sadness, distress | NR | OR (CI) | NS |
|  |  |  |  |  |  |  | Diabetes | 2.322 (1.350-3.996) | OR (CI) | 0.002 |
|  |  |  |  |  |  |  | Digestive problems | NR | OR (CI) | NS |
|  |  |  |  |  |  |  | Frequency of visits | NR | OR (CI) | NS |
|  |  |  |  |  |  |  | Genitourinary problems (including recurrent UTIs) | 2.455 (1.419-4.248) | OR (CI) | 0.001 |
|  |  |  |  |  |  |  | Haematological problems | NR | OR (CI) | NS |
|  |  |  |  |  |  |  | Hearing problems | NR | OR (CI) | NS |
|  |  |  |  |  |  |  | Heart problems | NR | OR (CI) | NS |
|  |  |  |  |  |  |  | Hypercholesterolemia | NR | OR (CI) | NS |
|  |  |  |  |  |  |  | Hypertension | 1.695 (1.044-2.753) | OR (CI) | 0.033 |
|  |  |  |  |  |  |  | Insomnia | NR | OR (CI) | NS |
|  |  |  |  |  |  |  | Living children | NR | OR (CI) | NS |
|  |  |  |  |  |  |  | Marital status | NR | OR (CI) | NS |
|  |  |  |  |  |  |  | Memory problems | NR | OR (CI) | NS |
|  |  |  |  |  |  |  | Mobility (EQ-5D) | NR | OR (CI) | NS |
|  |  |  |  |  |  |  | Mouth and dental problems | NR | OR (CI) | NS |
|  |  |  |  |  |  |  | Nervous system diseases | NR | OR (CI) | NS |
|  |  |  |  |  |  |  | Other endocrine and metabolic problems | NR | OR (CI) | NS |
|  |  |  |  |  |  |  | Pain, discomfort (EQ-5D) | NR | OR (CI) | NS |
|  |  |  |  |  |  |  | Parkinson’s | NR | OR (CI) | NS |
|  |  |  |  |  |  |  | Participates in active leisure | NR | OR (CI) | NS |
|  |  |  |  |  |  |  | Participates in cultural leisure | NR | OR (CI) | NS |
|  |  |  |  |  |  |  | Participates in passive leisure | 1.616 (0.968-2.700) | OR (CI) | 0.067 |
|  |  |  |  |  |  |  | Participates in social leisure | 2.242 (1.170-4.299) | OR (CI) | 0.015 |
|  |  |  |  |  |  |  | Personal care (EQ-5D) | NR | OR (CI) | NS |
|  |  |  |  |  |  |  | Receives visits | NR | OR (CI) | NS |
|  |  |  |  |  |  |  | Sex | NR | OR (CI) | NS |
|  |  |  |  |  |  |  | Skin problems | NR | OR (CI) | NS |
|  |  |  |  |  |  |  | Vision problems | NR | OR (CI) | NS |
| Netten et al, 1995 (33)  UK  Longitudinal study    QAS: 3 | n=73 | NR | 87 (SD NR)  75% female | Nursing home, n=1 | Baseline data collection 1990 to 1994, follow up for four years, data collected using assessment within the nursing home.  Analysis: Cox Proportional Hazard Model | 58% residents died within four years | Age - years above 65 | 1.05 | HR | NR |
|  |  |  |  |  |  |  | Barthel Score - 1 to 18 (lower score=greater dependence) | 0.88 | HR | NR |
|  |  |  |  |  |  |  | Sex (male ref) | 0.5 | HR | NR |
| Nygaard and Laake, 1990 (34)  Norway  Retrospective cohort study    QAS: 5 | N=318 | NR | 81 (SD NR)  70.1% female | Nursing home, n=1 | Baseline data collection 1980 to 1984, follow up for one to two years. Data collection not reported.  Analysis: Cox Proportional Hazard Model | 43.7% residents died within two years | Age | 1.48 (1.02-2.14) | OR (CI) | NR |
|  |  |  |  |  |  |  | Dementia | 0.48 (0.52-1.05) | OR (CI) | NR |
|  |  |  |  |  |  |  | Sex (male ref) | 0.74 (0.47-1.30) | OR (CI) | NR |
|  |  |  |  |  |  |  | Stroke | 0.79 (0.33-0.70) | OR (CI) | NR |
| Porock et al, 2005 (35)  USA  Retrospective cohort study    QAS: 11 | n=43,510 | Residents aged 65 and over | NR  73.56% female | LTCF, n=NR | Baseline data collection 1999 to 2000, follow up for six months. Data collection using MDS.  Analysis: Logistic Regression | 23% residents died within six months | Activities of daily livings (lower scores = less dependence) | 1.280 (1.254-1.306) | OR (CI) | NR |
|  |  |  |  |  |  |  | Affect change | NR | OR (CI) | NS |
|  |  |  |  |  |  |  | Age | NR | OR (CI) | NS |
|  |  |  |  |  |  |  | Alzheimer’s disease or Dementia | 0.787 (0.737-0.840) | OR (CI) | NR |
|  |  |  |  |  |  |  | Cancer | NR | OR (CI) | NS |
|  |  |  |  |  |  |  | Chronic heart failure | 1.458 (1.367-1.555) | OR (CI) | NR |
|  |  |  |  |  |  |  | Cognitive function (CPS) (lower scores = less impairment) | 1.095 (1.073-1.117) | OR (CI) | NR |
|  |  |  |  |  |  |  | Communication problems | NR | OR (CI) | NS |
|  |  |  |  |  |  |  | COPD | NR | OR (CI) | NS |
|  |  |  |  |  |  |  | Dehydration | 1.585 (1.416-1.774) | OR (CI) | NR |
|  |  |  |  |  |  |  | Deteriorating condition | NR | OR (CI) | NS |
|  |  |  |  |  |  |  | Edema | NR | OR (CI) | NS |
|  |  |  |  |  |  |  | Falls | NR | OR (CI) | NS |
|  |  |  |  |  |  |  | Infection, antibiotic-resistant infection | NR | OR (CI) | NS |
|  |  |  |  |  |  |  | Infection, Clostridium difficile | NR | OR (CI) | NS |
|  |  |  |  |  |  |  | Infection, pneumonia | NR | OR (CI) | NS |
|  |  |  |  |  |  |  | Infection, tuberculosis | NR | OR (CI) | NS |
|  |  |  |  |  |  |  | Loss of appetite | 1.589 (1.496-1.668) | OR (CI) | NR |
|  |  |  |  |  |  |  | Loss of spouse | NR | OR (CI) | NS |
|  |  |  |  |  |  |  | No. of times hospitalized in the past 90 days | NR | OR (CI) | NS |
|  |  |  |  |  |  |  | Pain | NR | OR (CI) | NS |
|  |  |  |  |  |  |  | Pain, moderate to severe nearly every day | NR | OR (CI) | NS |
|  |  |  |  |  |  |  | Parkinson’s disease | NR | OR (CI) | NS |
|  |  |  |  |  |  |  | Recent admission to nursing home | NR | OR (CI) | NS |
|  |  |  |  |  |  |  | Renal disease/failure | 1.856 (1.632-2.110) | OR (CI) | NR |
|  |  |  |  |  |  |  | Sex (female ref) | 1.801 (1.689-1.921) | OR (CI) | NR |
|  |  |  |  |  |  |  | Shortness of birth | 2.192 (2.019-2.381) | OR (CI) | NR |
|  |  |  |  |  |  |  | Sleep - no further information | NR | OR (CI) | NS |
|  |  |  |  |  |  |  | Weight loss | 1.547 (1.428-1.676) | OR (CI) | NR |
| Rothera et al, 2002 (36)  England  Retrospective cohort study    QAS: 10 | n=641/n=514 | Newly admitted residents aged 65 years and over and funded by Social Services | NR    69.9% female | Residential, nursing and dual registered care home, n=NR | Baseline data collection 1997 to 1999, follow up for twenty months. Data collection using Social Services Assessments.    Analysis: Cox Regression Analysis | 31% residents in nursing homes and 17% in residential homes died within twenty months | Age - 85+ (ref) vs 65-74 | 0.70 (0.53 – 0.93) | OR (CI) | <0.01 |
|  |  |  |  |  |  |  | Age - 85+ (ref) vs 75-84 | 0.77 (0.64 – 0.91) | OR (CI) | <0.01 |
|  |  |  |  |  |  |  | Care home type - Residential (ref) vs Dual | 1.80 (1.46 – 2.21) | OR (CI) | <0.001 |
|  |  |  |  |  |  |  | Care home type - Residential (ref) vs Nursing | 1.85 (1.50 – 2.23) | OR (CI) | <0.001 |
|  |  |  |  |  |  |  | Continence - low (ref ) vs high | 1.1 (0.68 – 1.77) | OR (CI) | 0.91 |
|  |  |  |  |  |  |  | Continence - low (ref) vs medium | 1.0 (0.58 – 1.71) | OR (CI) | 0.91 |
|  |  |  |  |  |  |  | Mobility – low (ref) vs high | 2.40 (1.27-4.55) | OR (CI) | 0.02 |
|  |  |  |  |  |  |  | Mobility - low (ref) vs medium | 1.70 (0.88 – 3.27) | OR (CI) | 0.02 |
|  |  |  |  |  |  |  | Number of cognitive problems - Score of 1-3 (ref) vs 0 | 1.55 (0.96 – 2.49) | OR (CI) | 0.07 |
|  |  |  |  |  |  |  | Sex (female ref) | 1.71 (1.44 – 2.03) | OR (CI) | <0.001 |
|  |  |  |  |  |  |  | Source - community (ref) vs hospital | 1.32 (1.11 – 1.56) | OR (CI) | <0.01 |
| Shah et al 2013 (37)  England and Wales  Prospective cohort study    QAS: 14 | n=9,772/9,172 | Residents aged between 65–104 years | NR    Gender NR | Residential and nursing homes, n=NR | Baseline data collection 2009, follow up for one year. Data collection using the Health Improvement Network dataset.    Analysis: Cox Proportional Hazard Model | 26.2% residents died within one year | Age - 65–74 (ref) vs 75–84 | 1.49 | HR (CI) | NR |
|  |  |  |  |  |  |  | Age - 65–74 (ref) vs 85–94 | 2.19 | HR (CI) | NR |
|  |  |  |  |  |  |  | Age - 65–74 (ref) vs 95–104 | 3.05 | HR (CI) | NR |
|  |  |  |  |  |  |  | Asthma/COPD | 1.17 (1.04–1.33) | HR (CI) | NR |
|  |  |  |  |  |  |  | Cancer | 1.36 (1.21–1.53) | HR (CI) | NR |
|  |  |  |  |  |  |  | Care home type: Residential (ref) vs Nursing | 1.48 (1.36–1.61) | HR (CI) | NR |
|  |  |  |  |  |  |  | Care home type: Residential (ref) vs Unclassified | 1.14 (1.01–1.30) | HR (CI) | NR |
|  |  |  |  |  |  |  | Clinical contacts - 0 (ref) vs 6+ | 1.65 (1.43–1.92) | HR (CI) | NR |
|  |  |  |  |  |  |  | Clinical contacts - 0 (ref) vs 1–2 | 1.04 (0.92–1.18) | HR (CI) | NR |
|  |  |  |  |  |  |  | Clinical contacts - 0 (ref) vs 3–5 | 1.27 (1.12–1.45) | HR (CI) | NR |
|  |  |  |  |  |  |  | Coronary heart disease | 0.99 (0.89–1.09) | HR (CI) | NR |
|  |  |  |  |  |  |  | Dementia | 1.26 (1.16–1.37) | HR (CI) | NR |
|  |  |  |  |  |  |  | Deprivation (IMD quintiles): 1 (ref) vs 5 (lower scores = less deprivation) | 1.02 (0.87–1.19) | HR (CI) | NR |
|  |  |  |  |  |  |  | Deprivation IMD quintiles: One (ref) vs Four | 1.04 ( 0.92–1.18) | HR (CI) | NR |
|  |  |  |  |  |  |  | Deprivation IMD quintiles: One (ref) vs Three | 0.99 ( 0.88–1.12) | HR (CI) | NR |
|  |  |  |  |  |  |  | Deprivation IMD quintiles: One (ref) vs Two | 1.07 (0.96–1.21) | HR (CI) | NR |
|  |  |  |  |  |  |  | Diabetes | 0.99 (0.88–1.12) | HR (CI) | NR |
|  |  |  |  |  |  |  | Heart failure | 1.13 (0.99–1.29) | HR (CI) | NR |
|  |  |  |  |  |  |  | Number of drugs - 0-2 (ref) vs 11+ | 1.59 (1.26–2.00) | HR (CI) | NR |
|  |  |  |  |  |  |  | Number of drugs - 0-2 (ref) vs 3–5 | 1.16 (0.92–1.47) | HR (CI) | NR |
|  |  |  |  |  |  |  | Number of drugs - 0-2 (ref) vs 6–10 | 1.34 (1.07–1.67) | HR (CI) | NR |
|  |  |  |  |  |  |  | Parkinson’s disease | 1.23 (1.04–1.45) | HR (CI) | NR |
|  |  |  |  |  |  |  | Practice region - South (ref) vs North | 1.04 (0.96–1.12) | HR (CI) | NR |
|  |  |  |  |  |  |  | Registration with GP - 5 years (ref) vs <90 days: | 1.45 (1.24–1.71) | HR (CI) | NR |
|  |  |  |  |  |  |  | Registration with GP - 5 years (ref) vs 1–5 years | 1.14 (1.05–1.25) | HR (CI) | NR |
|  |  |  |  |  |  |  | Registration with GP - 5 years (ref) vs 90–365 days: | 1.09 (0.97–1.23) | HR (CI) | NR |
|  |  |  |  |  |  |  | Sex (female ref) | 1.49 (1.36–1.63) | HR (CI) | NR |
|  |  |  |  |  |  |  | Stroke | 1.11 ( 1.00–1.23) | HR (CI) | NR |
| Sharifi et al, 2012 (38)  Iran  Prospective cohort study    QAS: 9 | n=247/  n=145 | Residents aged 65 years and older from the KES prospective study | 76.7 (18.4)    59.7% female | Nursing home, n=1 | Baseline data collection 2006 to 2009, follow up for thirty-nine months. Data collection using the KCF computerised bank, medical records and care home staff.  Analysis: Cox Proportional Hazards Model | 30% residents died within thirty nine months | Age | 1.04 (1.01-1.07) | HR (CI) | NR |
|  |  |  |  |  |  |  | Albumin | 0.19 (0.10–0.36) | HR (CI) | <0.05 |
|  |  |  |  |  |  |  | Apolipoprotein A | NR | HR (CI) | NS |
|  |  |  |  |  |  |  | Apolipoprotein B | NR | HR (CI) | NS |
|  |  |  |  |  |  |  | Barthel Index (lower score=greater dependence) | 0.62 (0.49–0.78) | HR (CI) | <0.05 |
|  |  |  |  |  |  |  | Barthel Index - Components: Bladder incontinence | 1.56 (1.02–2.48) | HR (CI) | <0.05 |
|  |  |  |  |  |  |  | Barthel Index - Components: Bowel incontinence | 2.14 (1.32–3.47) | HR (CI) | <0.05 |
|  |  |  |  |  |  |  | Barthel Index - Components: Dressing inability | 2.12 (1.33–3.39) | HR (CI) | <0.05 |
|  |  |  |  |  |  |  | Barthel Index - Components: Immobility | 1.79 (1.32–3.47) | HR (CI) | <0.05 |
|  |  |  |  |  |  |  | Barthel Index - Components: Stairs climbing inability | 3.05 (1.38–6.76) | HR (CI) | <0.05 |
|  |  |  |  |  |  |  | Barthel Index - Components: Transferring inability | 2.51 (1.34–4.64) | HR (CI) | <0.05 |
|  |  |  |  |  |  |  | Barthel Index - First quartile (ref) vs Fourth quartile | 0.23 (0.10–0.50) | HR (CI) | <0.05 |
|  |  |  |  |  |  |  | Barthel Index - First quartile (ref) vs Second quartile | 0.47 (0.26–0.83) | HR (CI) | <0.05 |
|  |  |  |  |  |  |  | Barthel Index - First quartile (ref) vs Third quartile | 0.44 (0.24–0.84) | HR (CI) | <0.05 |
|  |  |  |  |  |  |  | BMI | 0.81 (0.57-1.16) | HR (CI) | NS |
|  |  |  |  |  |  |  | Bodyweight - Ideal bodyweight (ref) vs Overweight | 0.82 (0.50-1.34) | HR (CI) | NS |
|  |  |  |  |  |  |  | Bodyweight - Ideal bodyweight (ref) vs Underweight | 1.07 (0.45-2.56) | HR (CI) | NS |
|  |  |  |  |  |  |  | BUN-to-creatinine ratio > 20 | 2.60 (1.59–4.25) | HR (CI) | <0.05 |
|  |  |  |  |  |  |  | Calf circumference | 0.92 (0.87-0.98) | HR (CI) | NS |
|  |  |  |  |  |  |  | Cholesterol | NR | HR (CI) | NS |
|  |  |  |  |  |  |  | Cognition (MMSE) | 1.14 (0.70-1.89) | HR (CI) | NS |
|  |  |  |  |  |  |  | Cognition (MMSE) - Normal Cognition (ref) vs Mild cognition impairment | 1.01 (0.56-1.84) | HR (CI) | NS |
|  |  |  |  |  |  |  | Cognition (MMSE) - Normal Cognition (ref) vs Moderate cognition impairment | 1.08 (0.61-1.92) | HR (CI) | NS |
|  |  |  |  |  |  |  | Cognition (MMSE) Components - Place orientation | 0.84 (0.71–0.99) | HR (CI) | <0.05 |
|  |  |  |  |  |  |  | Cognition (MMSE) Components - Time orientation | 0.87 (0.75–1.02) | HR (CI) | NS |
|  |  |  |  |  |  |  | Depression (GDS) (lower score = lower depression) | 1.26 (1.00–1.58) | HR (CI) | <0.05 |
|  |  |  |  |  |  |  | Depression (GDS) Normal (ref) vs Mild depression | 1.00 (0.54-1.87) | HR (CI) | NS |
|  |  |  |  |  |  |  | Depression (GDS) Normal (ref) vs Moderate depression | 1.51 (0.80-2.85) | HR (CI) | NS |
|  |  |  |  |  |  |  | Depression (GDS) Normal (ref) vs Severe depression | 1.07 (0.44-2.62) | HR (CI) | NS |
|  |  |  |  |  |  |  | Diabetes mellitus | 1.13 (0.65–1.95) | HR (CI) | NS |
|  |  |  |  |  |  |  | Diuretics use | 1.38 (0.60–2.37) | HR (CI) | NS |
|  |  |  |  |  |  |  | Erythrocyte sedimentation rate | NR | HR (CI) | NS |
|  |  |  |  |  |  |  | Hearing loss | 1.90 (1.18–3.06) | HR (CI) | <0.05 |
|  |  |  |  |  |  |  | Hemoglobin | 0.79 (0.71–0.87) | HR (CI) | <0.05 |
|  |  |  |  |  |  |  | High sensitive C-reactive protein | NR | HR (CI) | NS |
|  |  |  |  |  |  |  | High-density lipoprotein | NR | HR (CI) | NS |
|  |  |  |  |  |  |  | History of Coronary Artery Disease | 1.79 (1.13–2.84) | HR (CI) | <0.05 |
|  |  |  |  |  |  |  | History of stroke | 1.12 (0.56–2.76) | HR (CI) | NS |
|  |  |  |  |  |  |  | Hypertension | 1.01 (0.28–3.71) | HR (CI) | NS |
|  |  |  |  |  |  |  | Lipoprotein | NR | HR (CI) | NS |
|  |  |  |  |  |  |  | Low-density lipoprotein | NR | HR (CI) | NS |
|  |  |  |  |  |  |  | Mid-arm circumference | 0.93 (0.87-0.99) | HR (CI) | NS |
|  |  |  |  |  |  |  | Nutrition - (MNA) | 1.72 (1.15–2.57) | HR (CI) | <0.05 |
|  |  |  |  |  |  |  | Nutrition - (MNA) Well-nourished (ref) vs At risk | 1.92 (1.15–3.18) | HR (CI) | <0.05 |
|  |  |  |  |  |  |  | Nutrition - (MNA) Well-nourished (ref) vs Malnourished | 2.44 (0.84–7.08) | HR (CI) | NS |
|  |  |  |  |  |  |  | Polypharmacy | 1.38 (0.60–2.30) | HR (CI) | NS |
|  |  |  |  |  |  |  | Pressure Ulcers - Norton Index (higher scores = better prognosis) | 1.86 (1.09–3.18) | HR (CI) | <0.05 |
|  |  |  |  |  |  |  | Red blood cell | NR | HR (CI) | NS |
|  |  |  |  |  |  |  | Serum albumin | NR | HR (CI) | NS |
|  |  |  |  |  |  |  | Serum insulin | NR | HR (CI) | NS |
|  |  |  |  |  |  |  | Sleep duration per day | NR | HR (CI) | NS |
|  |  |  |  |  |  |  | Smoking | 2.22 (0.80–6.14) | HR (CI) | NS |
|  |  |  |  |  |  |  | Smoking - non smoker (ref) vs ex smoker | 2.28 (0.82–6.32) | HR (CI) | NS |
|  |  |  |  |  |  |  | Smoking - non smoker (ref) vs smoker | 1.17 (0.28–4.76) | HR (CI) | NS |
|  |  |  |  |  |  |  | Total protein | NR | HR (CI) | NS |
|  |  |  |  |  |  |  | Total protein | 0.46 (0.27–0.77) | HR (CI) | <0.05 |
|  |  |  |  |  |  |  | Triglyceride | NR | HR (CI) | NS |
|  |  |  |  |  |  |  | Visual loss | 1.23 (0.71–1.79) | HR (CI) | NS |
|  |  |  |  |  |  |  | Waist-to-hip ratio | NR | HR (CI) | NS |
|  |  |  |  |  |  |  | White blood cell | NR | HR (CI) | NS |
| Sokejima et al, 1996 (39)  Japan  NR    QAS: 11 | n=451/  n=407 | Newly admitted residents aged 65 years and over | 78.3 (6.7)  66.6% female | Nursing homes, n=3 | Baseline data collection 1976 to 1990, follow up for five years. Data collection not reported.  Analysis: Cox Proportional Hazards Model | 44.2% residents died within five years | Age - 65-75 (ref) vs 76+ | 1.27 (0.92-1.74) | RH (CI) | NS |
|  |  |  |  |  |  |  | Mobile without stroke (ref) vs 1 year after admission of 'immobile with stroke' | 1.38 (1.17-1.62) | RH (CI) | <0.001 |
|  |  |  |  |  |  |  | Mobile without stroke (ref) vs Immobile without stroke | 3.62 (2.58-5.09) | RH (CI) | <0.0001 |
|  |  |  |  |  |  |  | Mobile without stroke (ref) vs Mobile with stroke | 2.20 (1.07-4.51) | RH (CI) | <0.05 |
|  |  |  |  |  |  |  | Sex (female ref) | 1.16 (0.85-1.59) | RH (CI) | NS |

| Spector and Takada, 1991 (40)  USA  Prospective cohort study  QAS: 9 | n=2,603/ n= | All residents residing in nursing homes | NR  NR | Nursing homes, n=80 | Baseline data collection from Rhode Island Nursing Home Study between 1984 to 1986, follow up for five to seven months - medical charts reviewed by the nurses. Facility data from other sources.  Analysis: Logistic Regression | 9.9% residents died within seven months | Affective disorders | NR | OR | NS |
| --- | --- | --- | --- | --- | --- | --- | --- | --- | --- | --- |
|  |  |  |  |  |  |  | Age | NR | OR | NS |
|  |  |  |  |  |  |  | Cancer | NR | OR | NS |
|  |  |  |  |  |  |  | Cognitive impairment | NR | OR | NS |
|  |  |  |  |  |  |  | COPD | NR | OR | NS |
|  |  |  |  |  |  |  | Disruptive behaviour | NR | OR | NS |
|  |  |  |  |  |  |  | Facility mean ADL score | NR | OR | NS |
|  |  |  |  |  |  |  | Facility received federal citations | 0.67 | OR | NS |
|  |  |  |  |  |  |  | For profit status (not for profit status reference) | 0.42 | OR | 0.01 |
|  |  |  |  |  |  |  | Functioning (Katz Index of Activities of Daily Living) | NR | OR | NS |
|  |  |  |  |  |  |  | Length of stay > 3months | NR | OR | NS |
|  |  |  |  |  |  |  | Mean organised activity days/resident/month (<3 reference vs >6) | 0.45 | OR | 0.05 |
|  |  |  |  |  |  |  | Mean organised activity days/resident/month (<3 reference vs 3-6) | 0.63 | OR | 0.05 |
|  |  |  |  |  |  |  | Number of beds | NR | OR | NS |
|  |  |  |  |  |  |  | Operating cost | NR | OR | NS |
|  |  |  |  |  |  |  | Percentage of residents with more than 7 medications | NR | OR | NS |
|  |  |  |  |  |  |  | Percentage of residents with skilled care | NR | OR | NS |
|  |  |  |  |  |  |  | Receipt of therapies | NR | OR | NS |
|  |  |  |  |  |  |  | Receipt of therapies | NR | OR | NS |
|  |  |  |  |  |  |  | Receipt of skilled services | NR | OR | NS |
|  |  |  |  |  |  |  | Resident days private pay (<10% reference vs > 40%) | 0.86 | OR | NS |
|  |  |  |  |  |  |  | Resident days private pay (<10% reference vs 10 - 40%) | 1.03 | OR | NS |
|  |  |  |  |  |  |  | Residents with 1+ psychoactive drug (<10% reference vs > 20%) | 1.1 | OR | NS |
|  |  |  |  |  |  |  | Residents with 1+ psychoactive drug (<10% reference vs >10 - 20%) | 0.99 | OR | NS |
|  |  |  |  |  |  |  | Residents with catheters (0% reference vs >10%) | 1.66 | OR | NS |
|  |  |  |  |  |  |  | Residents with catheters (0% reference vs 1%-10%) | 1.91 | OR | NS |
|  |  |  |  |  |  |  | Residents with skin care (<20% reference vs > 40%) | 0.90 | OR | NS |
|  |  |  |  |  |  |  | Residents with skin care (<20% reference vs >20 - 40%) | 0.92 | OR | NS |
|  |  |  |  |  |  |  | Schizophrenia or mental retardation | NR | OR | NS |
|  |  |  |  |  |  |  | Staff ratio (high staff/ high ADLs reference vs low staff) | 1.23 | OR | NS |
|  |  |  |  |  |  |  | Staff ratio (high staff/ high ADLs reference vs moderate staff) | 1.5 | OR | NS |
|  |  |  |  |  |  |  | Staff turnover | NR | OR | NS |

| Suh et al, 2005 (41)  Korea  Prospective follow-up    QAS: 13 | n=145 | Residents aged over 50 with AD | 80.8 (7.9)  84.4% female | Nursing home, n=1 | Baseline data collection 2002 to 2003, follow up for six months and one year. Data collection using face to face interviews.  Analysis: Cox proportional hazards model | 17.2% residents died within one year | Age | 1.03 (1.00–1.07) | RR (CI) | <0.05 |
| --- | --- | --- | --- | --- | --- | --- | --- | --- | --- | --- |
|  |  |  |  |  |  |  | Auditory hallucinations (BEHAVE-AD ) | 1.25 (1.01–1.54) | RR (CI) | <0.05 |
|  |  |  |  |  |  |  | Basic ADL (DAD-K) | 0.97(0.96–0.99) | RR (CI) | <0.05 |
|  |  |  |  |  |  |  | Behavioural and psychological symptoms of dementia (BEHAVE-AD ) (higher score - more severe) | 1.03 (1.01–1.05) | RR (CI) | <0.05 |
|  |  |  |  |  |  |  | Cognitive status - (MMSE score) (lower score - poorer status) | 0.88 (0.81–0.96) | RR (CI) | <0.05 |
|  |  |  |  |  |  |  | Cognitive status - Alzheimer’s Disease Assessment Scale (ADAS-K-cog ) (higher score - poorer status) | 1.04 (1.01–1.07) | RR (CI) | <0.05 |
|  |  |  |  |  |  |  | Delusions (BEHAVE-AD ) | 1.11 (1.01–1.22) | RR (CI) | <0.05 |
|  |  |  |  |  |  |  | Dementia Severity - Global Deterioration Scale (GDS) | 2.76 (1.59–4.80) | RR (CI) | <0.05 |
|  |  |  |  |  |  |  | Depression (BEHAVE-AD ) | 1.08(1.03–1.14) | RR (CI) | <0.05 |
|  |  |  |  |  |  |  | Duration of AD at study entry | 1.09 (1.04–1.12) | RR (CI) | <0.05 |
|  |  |  |  |  |  |  | Education (years) | 0.98 (0.90–1.06) | RR (CI) | NS |
|  |  |  |  |  |  |  | Functional ability - (DAD-K) (lower score - poorer status) | 0.96(0.93–0.98) | RR (CI) | <0.05 |
|  |  |  |  |  |  |  | Gait disturbance | NR | RR (CI) | NS |
|  |  |  |  |  |  |  | Gender | NR | RR (CI) | NS |
|  |  |  |  |  |  |  | Initiation (DAD-K) | 0.97(0.95–0.98) | RR (CI) | <0.05 |
|  |  |  |  |  |  |  | Instrumental ADL (DAD-K) | 0.97(0.94–0.99) | RR (CI) | <0.05 |
|  |  |  |  |  |  |  | Performance (DAD-K) | 0.96(0.93–0.98) | RR (CI) | <0.05 |
|  |  |  |  |  |  |  | Planning and organization (DAD-K) | 0.96(0.94–0.99) | RR (CI) | <0.05 |
|  |  |  |  |  |  |  | Sensory impairment (hearing and vision) | NR | RR (CI) | NS |
|  |  |  |  |  |  |  | Tactile hallucinations (BEHAVE-AD ) | 2.97(1.50–5.88) | RR (CI) | <0.05 |
|  |  |  |  |  |  |  | Vascular risk factor (hypertension, heart disease, diabetes mellitus, hyperlipidemia) | 4.07(1.77–9.37) | RR (CI) | <0.05 |
|  |  |  |  |  |  |  | Wandering (BEHAVE-AD) | 2.18(1.20–3.96) | RR (CI) | <0.05 |
| Sund Levander et al, 2016 (42)  Sweden  Retrospective study    QAS: 9 | Cohort 1: n=262/  n=149 Cohort 2: n=210/  n=200 | Residents aged 65 years and older | Cohort 1: 84.4 (6.9) Cohort 2: 85.6 (6.9)    Cohort 1: 73% female  Cohort 2: 70% female | Nursing home, n=NR | Baseline data collection 2000 to 2004 (cohort 1), follow up for five years and 2007 (cohort 2), follow up for one year. Data collection using medical records, interviews with nursing staff or resident.  Analysis: Cox Proportional Hazard Model | Cohort 1: 28% residents died within five years  Cohort 2: 23% residents died within one year | **Cohort 1 – five year follow up:** |  |  |  |
|  |  |  |  |  |  |  | Activities of daily living | 0.844 (0.766–0.930) | HR (CI) | < 0.01 |
|  |  |  |  |  |  |  | Age | NR | HR (CI) | NS |
|  |  |  |  |  |  |  | Anti-depressants | NR | HR (CI) | NS |
|  |  |  |  |  |  |  | Autoimmune disease | 0.079 (0.009–0.685) | HR (CI) | < 0.05 |
|  |  |  |  |  |  |  | Body mass index | NR | HR (CI) | NS |
|  |  |  |  |  |  |  | Cardiovascular disease | NR | HR (CI) | NS |
|  |  |  |  |  |  |  | Chronic obstructive pulmonary disease | NR | HR (CI) | NS |
|  |  |  |  |  |  |  | Cortisone | NR | HR (CI) | NS |
|  |  |  |  |  |  |  | Dementia | NR | HR (CI) | NS |
|  |  |  |  |  |  |  | Diabetes | 3.587 (1.633–7.878) | HR (CI) | < 0.01 |
|  |  |  |  |  |  |  | Influenza vaccination | 0.455 (0.237–0.872) | HR (CI) | < 0.05 |
|  |  |  |  |  |  |  | Malnutrition | NR | HR (CI) | NS |
|  |  |  |  |  |  |  | Paracetamol (>= 3 g daily) | NR | HR (CI) | NS |
|  |  |  |  |  |  |  | Pneumocockiae vaccination | NR | HR (CI) | NS |
|  |  |  |  |  |  |  | Sedatives/tranquillisers | NR | HR (CI) | NS |
|  |  |  |  |  |  |  | Sex | NR | HR (CI) | NS |
|  |  |  |  |  |  |  | Stroke Factor | 2.308 (1.162–4.584) | HR (CI) | < 0.05 |
|  |  |  |  |  |  |  | Thyroid disease | NR | HR (CI) | NS |
|  |  |  |  |  |  |  | **Cohort 2 – one year follow up:** |  |  |  |
|  |  |  |  |  |  |  | Activities of daily living | 0.718 (0.644-0.801) | HR (CI) | < 0.001 |
|  |  |  |  |  |  |  | Age | NR | HR (CI) | NS |
|  |  |  |  |  |  |  | Anti-depressants | NR | HR (CI) | NS |
|  |  |  |  |  |  |  | Autoimmune disease | NR | HR (CI) | NS |
|  |  |  |  |  |  |  | Body mass index | 1.058 (1.000–1.119) | HR (CI) | < 0.05 |
|  |  |  |  |  |  |  | Cardiovascular disease | NR | HR (CI) | NS |
|  |  |  |  |  |  |  | Chronic obstructive pulmonary disease | NR | HR (CI) | NS |
|  |  |  |  |  |  |  | Cortisone | NR | HR (CI) | NS |
|  |  |  |  |  |  |  | Dementia | NR | HR (CI) | NS |
|  |  |  |  |  |  |  | Diabetes | NR | HR (CI) | NS |
|  |  |  |  |  |  |  | Influenza vaccination | 0.439 (0.208-0.924) | HR (CI) | < 0.05 |
|  |  |  |  |  |  |  | Malnutrition | 0.844 (0.766-0.930) | HR (CI) | < 0.001 |
|  |  |  |  |  |  |  | Paracetamol (>= 3 g daily) | 0.409 (0.207–0.808) | HR (CI) | < 0.05 |
|  |  |  |  |  |  |  | Pneumocockiae vaccination | NR | HR (CI) | NS |
|  |  |  |  |  |  |  | Sedatives/tranquillisers | 0.473 (0.256-0.873) | HR (CI) | < 0.05 |
|  |  |  |  |  |  |  | Sex | NR | HR (CI) | NS |
|  |  |  |  |  |  |  | Stroke Factor | NR | HR (CI) | NS |
|  |  |  |  |  |  |  | Thyroid disease | NR | HR (CI) | NS |
| Sung, 2014 (43)  South Korea  Retrospective study  QAS: 13 | n=195 | Newly admitted residents aged over 65 years. | 81.59 (7.66)  76.4% female | Nursing home, n=6 | Baseline data collection 2008 to 2012, follow up until death. Data collection using evaluation table data.  Analysis: Logistic Regression | 47.7% residents died within follow up period | ADL 0-8 score (ref) vs. 9-16 score (higher score = higher dependence) | 3.61 | OR | 0.014 |
|  |  |  |  |  |  |  | ADL 9-16 score (ref) vs. 17-24 score | 3.22 | OR | 0.004 |
|  |  |  |  |  |  |  | Arthritis | NR | OR | NS |
|  |  |  |  |  |  |  | Aspiration care | 1.69 | OR | 0.465 |
|  |  |  |  |  |  |  | Cancer | NR | OR | NS |
|  |  |  |  |  |  |  | Care for cancer pain | 2.14 | OR | 0.165 |
|  |  |  |  |  |  |  | Care for pressure sores | NR | OR | NS |
|  |  |  |  |  |  |  | Cognitive function | NR | OR | NS |
|  |  |  |  |  |  |  | Dementia | NR | OR | NS |
|  |  |  |  |  |  |  | Diabetes mellitus | NR | OR | NS |
|  |  |  |  |  |  |  | Dialysis care | NR | OR | NS |
|  |  |  |  |  |  |  | Dyspnea | 4.88 | OR | 0.001 |
|  |  |  |  |  |  |  | Hearing loss | NR | OR | NS |
|  |  |  |  |  |  |  | Hypertension | NR | OR | NS |
|  |  |  |  |  |  |  | Insertion of urinary catheters | NR | OR | NS |
|  |  |  |  |  |  |  | Lower back pain and sciatic pain | NR | OR | NS |
|  |  |  |  |  |  |  | Ostomy care | NR | OR | NS |
|  |  |  |  |  |  |  | Oxidization therapy | 1.86 | OR | 0.336 |
|  |  |  |  |  |  |  | Problematic behaviours 5-9 score (ref) vs. 10-14 | 1.29 | OR | 0.648 |
|  |  |  |  |  |  |  | Problematic behaviours 0-4 score (ref) vs. 5-9 (higher score = higher dependence) | 3.95 | OR | 0.015 |
|  |  |  |  |  |  |  | Range of motion (0-4 vs.5-8) (higher score = higher dependence) | 1.99 | OR | 0.086 |
|  |  |  |  |  |  |  | Sequelae of accidents such as fractures and dislocations | NR | OR | NS |
|  |  |  |  |  |  |  | Stroke | NR | OR | NS |
|  |  |  |  |  |  |  | Tracheostomy care | NR | OR | NS |
|  |  |  |  |  |  |  | Tube feeding | NR | OR | NS |
|  |  |  |  |  |  |  | Visual disturbance such as cataracts and glaucoma | NR | OR | NS |
| Troyer, 2004 (44)  USA  NR  QAS: 9 | n=  394,196 | NR | 76.62 (13.7)  Gender NR | Nursing facilities, n=677 | Baseline data collection 1986 to 1997, follow up for one to two years. Data collection using Florida Agency for Health Care Administration.  Analysis: Probit Model | NR | **Cohort 1: Death within one year** |  |  |  |
|  |  |  |  |  |  |  | Age | -0.003 | Probit Est | <0.01 |
|  |  |  |  |  |  |  | Arteriosclerotic heart disease | -0.012 | Probit Est | <0.05 |
|  |  |  |  |  |  |  | Brain/neurological disorder, including organic brain syndrome, Alzheimer’s and Parkinson’s Disease | -0.013 | Probit Est | <0.01 |
|  |  |  |  |  |  |  | Cancer | 0.0328 | Probit Est | <0.01 |
|  |  |  |  |  |  |  | Cerebrovascular accident | -0.011 | Probit Est | <0.05 |
|  |  |  |  |  |  |  | Chronic heart failure or hypertension | 0.025 | Probit Est | <0.01 |
|  |  |  |  |  |  |  | Facility characteristics: Chain owned | -0.005 | Probit Est | NS |
|  |  |  |  |  |  |  | Facility characteristics: Facility size (no of beds) | 0 | Probit Est | NS |
|  |  |  |  |  |  |  | Facility characteristics: Government owned | 0.11 | Probit Est | <0.05 |
|  |  |  |  |  |  |  | Facility characteristics: Market share | 0.013 | Probit Est | <0.05 |
|  |  |  |  |  |  |  | Facility characteristics: Non-profit | 0.003 | Probit Est | NS |
|  |  |  |  |  |  |  | Facility characteristics: Occupancy rate | 0.023 | Probit Est | <0.01 |
|  |  |  |  |  |  |  | Facility characteristics: Percent Medicaid | 0.151 | Probit Est | <0.01 |
|  |  |  |  |  |  |  | Facility characteristics: Percent private pay | 0.154 | Probit Est | <0.01 |
|  |  |  |  |  |  |  | Fractures/ musculoskeletal | -0.012 | Probit Est | <0.01 |
|  |  |  |  |  |  |  | Location characteristics: Beds per elderly capita | 0.117 | Probit Est | <0.05 |
|  |  |  |  |  |  |  | Location characteristics: Per Income Capital | 0.036 | Probit Est | <0.05 |
|  |  |  |  |  |  |  | Location characteristics: Population of individuals aged 65+ | -0.033 | Probit Est | <0.01 |
|  |  |  |  |  |  |  | Mental disorder | -0.073 | Probit Est | NS |
|  |  |  |  |  |  |  | Origin of Resident - assisted living facility | 0.014 | Probit Est | NS |
|  |  |  |  |  |  |  | Origin of Resident - home | -0.021 | Probit Est | <0.05 |
|  |  |  |  |  |  |  | Origin of Resident - hospital | -0.02 | Probit Est | <0.05 |
|  |  |  |  |  |  |  | Receiving Medicaid | 0.023 | Probit Est | <0.01 |
|  |  |  |  |  |  |  | Respiratory disorder/ disease | 0.048 | Probit Est | <0.01 |
|  |  |  |  |  |  |  | **Cohort 2: Death within two years:** |  |  |  |
|  |  |  |  |  |  |  | Age | 0.004 | Probit Est | <0.01 |
|  |  |  |  |  |  |  | Arteriosclerotic heart disease | -0.003 | Probit Est | NS |
|  |  |  |  |  |  |  | Brain/ neurological disorder, including organic brain syndrome, Alzheimer’s and Parkinson’s | 0.004 | Probit Est | NS |
|  |  |  |  |  |  |  | Cancer | 0.0328 | Probit Est | <0.01 |
|  |  |  |  |  |  |  | Cerebrovascular accident | -0.008 | Probit Est | NS |
|  |  |  |  |  |  |  | Chronic heart failure or hypertension | 0.027 | Probit Est | <0.01 |
|  |  |  |  |  |  |  | Facility characteristics: Chain owned | -0.009 | Probit Est | <0.05 |
|  |  |  |  |  |  |  | Facility characteristics: Facility size (no of beds) | 0 | Probit Est | NS |
|  |  |  |  |  |  |  | Facility characteristics: Government owned | 0.129 | Probit Est | <0.05 |
|  |  |  |  |  |  |  | Facility characteristics: Market share | -0.019 | Probit Est | <0.05 |
|  |  |  |  |  |  |  | Facility characteristics: Non-profit | 0.009 | Probit Est | NS |
|  |  |  |  |  |  |  | Facility characteristics: Occupancy rate | 0.038 | Probit Est | <0.01 |
|  |  |  |  |  |  |  | Facility characteristics: Percent Medicaid | 0.178 | Probit Est | <0.01 |
|  |  |  |  |  |  |  | Facility characteristics: Percent private pay | 0.188 | Probit Est | <0.01 |
|  |  |  |  |  |  |  | Fractures/ musculoskeletal | 0.131 | Probit Est | <0.01 |
|  |  |  |  |  |  |  | Location characteristics: Beds per elderly capita | -0.148 | Probit Est | <0.05 |
|  |  |  |  |  |  |  | Location characteristics: Per Income Capital | 0.076 | Probit Est | <0.01 |
|  |  |  |  |  |  |  | Location characteristics: Population of individuals aged 65+ | -0.043 | Probit Est | <0.01 |
|  |  |  |  |  |  |  | Mental disorder | -0.068 | Probit Est | <0.01 |
|  |  |  |  |  |  |  | Origin of Resident - assisted living facility | 0.035 | Probit Est | <0.01 |
|  |  |  |  |  |  |  | Origin of Resident - home | -0.023 | Probit Est | <0.05 |
|  |  |  |  |  |  |  | Origin of Resident - hospital | -0.038 | Probit Est | <0.01 |
|  |  |  |  |  |  |  | Receiving Medicaid | 0.054 | Probit Est | <0.01 |
|  |  |  |  |  |  |  | Respiratory disorder/ disease | 0.048 | Probit Est | <0.01 |
| van Dijk et al, 2005 (45)  USA  Retrospective cohort study    QAS: 7 | n= 44,062/  n=43,510 | Residents aged 65 and over with a full MDS assessment | 84.4 (7.8)  74% female | Nursing homes, n=522 | Baseline data collection 1999, follow up for one year. Data collection using MDS.  Analysis: Logistic Regression | 35% residents died within one year | Age | 1.034 (1.030–1.038) | OR (CI) | NR |
|  |  |  |  |  |  |  | Allergies | NR | OR (CI) | NS |
|  |  |  |  |  |  |  | Anaemia | NS | OR (CI) | NR |
|  |  |  |  |  |  |  | Arteriosclerotic heart disease | NR | OR (CI) | NS |
|  |  |  |  |  |  |  | Arthritis | NR | OR (CI) | NS |
|  |  |  |  |  |  |  | Asthma | NR | OR (CI) | NS |
|  |  |  |  |  |  |  | Bipolar disease | NR | OR (CI) | NS |
|  |  |  |  |  |  |  | Cancer | 374 (174–804) | OR (CI) | NR |
|  |  |  |  |  |  |  | Cancer by age | 0.059 (0.93–0.95) | OR (CI) | NR |
|  |  |  |  |  |  |  | Cerebral palsy | NR | OR (CI) | NS |
|  |  |  |  |  |  |  | Dementia (CPS) | NS | OR (CI) | NR |
|  |  |  |  |  |  |  | Depression | NR | OR (CI) | NS |
|  |  |  |  |  |  |  | Diabetes mellitus | 1.20 (1.14–1.27) | OR (CI) | NR |
|  |  |  |  |  |  |  | Dysrhythmias | NR | OR (CI) | NS |
|  |  |  |  |  |  |  | Emphysema/ COPD | 1.59 (1.51–1.68) | OR (CI) | NR |
|  |  |  |  |  |  |  | Eye disease | NR | OR (CI) | NS |
|  |  |  |  |  |  |  | Functioning (ADLs) (higher score = higher dependence) | 1.12 (1.12–1.13) | OR (CI) | NR |
|  |  |  |  |  |  |  | Heart failure | 1.59 (1.52–1.67) | OR (CI) | NR |
|  |  |  |  |  |  |  | Hypertension | NR | OR (CI) | NS |
|  |  |  |  |  |  |  | Hyperthyroidism | NR | OR (CI) | NS |
|  |  |  |  |  |  |  | Hypotension | NR | OR (CI) | NS |
|  |  |  |  |  |  |  | Hypothyroidism | NR | OR (CI) | NS |
|  |  |  |  |  |  |  | Multiple sclerosis | NR | OR (CI) | NS |
|  |  |  |  |  |  |  | Osteoporosis | NR | OR (CI) | NS |
|  |  |  |  |  |  |  | Other cardiovascular disease | NR | OR (CI) | NS |
|  |  |  |  |  |  |  | Parkinson’s disease | NR | OR (CI) | NS |
|  |  |  |  |  |  |  | Peripheral vascular disease | NR | OR (CI) | NS |
|  |  |  |  |  |  |  | Renal failure | 2.14 (1.90–2.41) | OR (CI) | NR |
|  |  |  |  |  |  |  | Schizophrenia | NR | OR (CI) | NS |
|  |  |  |  |  |  |  | Seizures | NR | OR (CI) | NS |
|  |  |  |  |  |  |  | Sex (female ref) | 1.71 (1.63–1.80) | OR (CI) | NR |
|  |  |  |  |  |  |  | Stroke | NR | OR (CI) | NS |
|  |  |  |  |  |  |  | Transient ischemic attack | NR | OR (CI) | NS |
|  |  |  |  |  |  |  | Traumatic brain injury | NR | OR (CI) | NS |
| van Dijk et al, 1996 (46)  Netherlands  Follow up study  QAS: 10 | n=606 | Newly admitted residents with dementia | 80.8 (6.8)    72.1% female | Nursing homes, n=1 | Baseline data collection 1982 to 1988, follow up for eight years. Data collection using retrospective chart review.  Analysis: Multivariate proportional hazards regression analysis | 65% residents died within eight years | Age | 1.04 (1.03-1.06) | RR (CI) | NR |
|  |  |  |  |  |  |  | Anaemia | NR | RR (CI) | NR |
|  |  |  |  |  |  |  | Atrial fibrillation | 2.0 (1.4-2.7) | RR (CI) | NR |
|  |  |  |  |  |  |  | Chronic lung disease | NR | RR (CI) | NR |
|  |  |  |  |  |  |  | Coming from home | 0.95 (0.8-1.2) | RR (CI) | NS |
|  |  |  |  |  |  |  | Coming from hospital | 1.2 (0.9-1.5) | RR (CI) | NS |
|  |  |  |  |  |  |  | Dementia | NR | RR (CI) | NR |
|  |  |  |  |  |  |  | Diabetes mellitus | 1.6 (1.2-2.1) | RR (CI) | NR |
|  |  |  |  |  |  |  | Faecal incontinence | NR | RR (CI) | NR |
|  |  |  |  |  |  |  | Hearing impairment | NR | RR (CI) | NR |
|  |  |  |  |  |  |  | Heart failure | 1.7 (1.2-2.4) | RR (CI) | NR |
|  |  |  |  |  |  |  | Hip fracture | NR | RR (CI) | NR |
|  |  |  |  |  |  |  | Hypertension | NR | RR (CI) | NR |
|  |  |  |  |  |  |  | Malignancy | 2.2 (1.4-3.3) | RR (CI) | NR |
|  |  |  |  |  |  |  | Parkinsonism | 1.9 (1.4-2.5) | RR (CI) | NR |
|  |  |  |  |  |  |  | Pressure sores | 1.8 (1.2-2.6) | RR (CI) | NR |
|  |  |  |  |  |  |  | Previous hip operation | NR | RR (CI) | NR |
|  |  |  |  |  |  |  | Previous myocardial infarction | NR | RR (CI) | NR |
|  |  |  |  |  |  |  | Previous stroke | NR | RR (CI) | NR |
|  |  |  |  |  |  |  | Previous TIA | NR | RR (CI) | NR |
|  |  |  |  |  |  |  | Pulmonary infection and stroke | 16.4 (7.4-43.8) | RR (CI) | NR |
|  |  |  |  |  |  |  | Pulmonary infection, no stroke | 1.8(1.3-2.4) | RR (CI) | NR |
|  |  |  |  |  |  |  | Severity of dementia (BOP) | NR | RR (CI) | NR |
|  |  |  |  |  |  |  | Sex (female ref) | 1.7 (1.4-2.1) | RR (CI) | NR |
|  |  |  |  |  |  |  | Urinary incontinence | 1.3 (1.1-1.6) | RR (CI) | NR |
|  |  |  |  |  |  |  | Urinary tract infection | NR | RR (CI) | NR |
|  |  |  |  |  |  |  | Visual problems | 1.3 (1.0-1.6 | RR (CI) | NR |
| Wallace and Prevost, 2006 (47)  USA  Secondary analysis  QAS: 8 | n=21,852 | NR | NR  70% female | Nursing facilities, n=111 | Baseline data collection January to June 2003, follow up for six months. Data collection using the MDS.  Analysis: Logistic Regression | 17.5% residents died within six months | Bowel incontinence | 1.1 | OR | ≤ 0.0001 |
|  |  |  |  |  |  |  | Cognitive measures: disorganized speech, recent onset | NR | OR | NS |
|  |  |  |  |  |  |  | Current condition unstable | 1.1 | OR | ≤ 0.0001 |
|  |  |  |  |  |  |  | Days receives diuretics | 1 | OR | ≤ 0.0001 |
|  |  |  |  |  |  |  | Dehydration | NR | OR | NS |
|  |  |  |  |  |  |  | Easily distracted | 0.8 | OR | ≤ 0.0001 |
|  |  |  |  |  |  |  | Fed through tube | 0.7 | OR | ≤ 0.0001 |
|  |  |  |  |  |  |  | Fell in past 31 to 180 days | 1.2 | OR | ≤ 0.0001 |
|  |  |  |  |  |  |  | Hip fracture in last 180 days | 0.7 | OR | ≤ 0.0001 |
|  |  |  |  |  |  |  | Inability to feed self | 1.1 | OR | ≤ 0.0001 |
|  |  |  |  |  |  |  | Inability to get out of bed | 1.3 | OR | ≤ 0.0001 |
|  |  |  |  |  |  |  | Inability to make own decisions | 1.1 | OR | ≤ 0.0001 |
|  |  |  |  |  |  |  | Inability to perform personal hygiene | 1.1 | OR | ≤ 0.0001 |
|  |  |  |  |  |  |  | Inability to walk in corridor | 1.1 | OR | ≤ 0.0001 |
|  |  |  |  |  |  |  | Inability to walk on unit | 1.1 | OR | ≤ 0.0001 |
|  |  |  |  |  |  |  | Insufficient fluid intake | NR | OR | NS |
|  |  |  |  |  |  |  | Limitation in range of motion of leg | 0.8 | OR | ≤ 0.0001 |
|  |  |  |  |  |  |  | Limited time involved in activities | 1.4 | OR | ≤ 0.0001 |
|  |  |  |  |  |  |  | Loss of voluntary movement of hand | 0.9 | OR | ≤ 0.0001 |
|  |  |  |  |  |  |  | Oral debris | NR | OR | NS |
|  |  |  |  |  |  |  | Other fracture in last 180 days −0.514 0.6 | 0.6 | OR | ≤ 0.0001 |
|  |  |  |  |  |  |  | Overall decline in condition | 1.1 | OR | ≤ 0.0001 |
|  |  |  |  |  |  |  | Periods of lethargy | 1.3 | OR | ≤ 0.0001 |
|  |  |  |  |  |  |  | Presence of indwelling catheter | 1.3 | OR | ≤ 0.0001 |
|  |  |  |  |  |  |  | Receives suctioning | NR | OR | NS |
|  |  |  |  |  |  |  | Recent weight loss | 1.6 | OR | ≤ 0.0001 |
|  |  |  |  |  |  |  | Recurrent lung aspirations | NR | OR | NS |
|  |  |  |  |  |  |  | Resists care, not easily altered | 1.3 | OR | ≤ 0.0001 |
|  |  |  |  |  |  |  | Sad or pained facial expressions | 1.1 | OR | ≤ 0.0001 |
|  |  |  |  |  |  |  | Short-term memory loss | 1.2 | OR | ≤ 0.0001 |
|  |  |  |  |  |  |  | Skin ulcers: Higher stage pressure ulcers | 1.1 | OR | ≤ 0.0001 |
|  |  |  |  |  |  |  | Skin ulcers: Higher stage stasis ulcers | 1.2 | OR | ≤ 0.0001 |
|  |  |  |  |  |  |  | Skin ulcers: Number of stage 1 ulcers | 1.1 | OR | ≤ 0.0001 |
|  |  |  |  |  |  |  | Terminal diagnosis | 3.1 | OR | ≤ 0.0001 |
| Woo et al, 1989 (48)  Hong Kong  NR  QAS: 7 | n=208 | Either all residents in the LTCF or residents referred by relatives, social workers, or doctors | 75.6 (9.6) (male) / 79.5 (8.4) (females)    72.1% female | Chronic care institutions, n=4 | Baseline data collection 1987 to 1998, follow up for three months. Data collection by study author and nurse.    Analysis: Stepwise Regression Analysis | 13.5 % residents died within three months | % neutrophils | NR | PCC | NS |
|  |  |  |  |  |  |  | Age | NR | PCC | NS |
|  |  |  |  |  |  |  | Albumin | NR | PCC | NS |
|  |  |  |  |  |  |  | Albumin adjusted calcium | NR | PCC | NS |
|  |  |  |  |  |  |  | Ascorbic acid | NR | PCC | NS |
|  |  |  |  |  |  |  | Aspartate aminotransferase activities | NR | PCC | NS |
|  |  |  |  |  |  |  | Beta carotene | NR | PCC | NS |
|  |  |  |  |  |  |  | Blindness | NR | PCC | NS |
|  |  |  |  |  |  |  | Calcium | NR | PCC | NS |
|  |  |  |  |  |  |  | Cerebrovascular accident | NR | PCC | NS |
|  |  |  |  |  |  |  | Cholesterol (umol/l) | NR | PCC | NS |
|  |  |  |  |  |  |  | Chronic heart disease | NR | PCC | NS |
|  |  |  |  |  |  |  | Chronic lung diseases | NR | PCC | NS |
|  |  |  |  |  |  |  | Complete blood count | NR | PCC | NS |
|  |  |  |  |  |  |  | Copper | NR | PCC | NS |
|  |  |  |  |  |  |  | Corrected arm muscle area (cm2) | NR | PCC | NS |
|  |  |  |  |  |  |  | Creatinine | NR | PCC | NS |
|  |  |  |  |  |  |  | Cyanocobalamin | NR | PCC | NS |
|  |  |  |  |  |  |  | Dementia | NR | PCC | NS |
|  |  |  |  |  |  |  | Duration of stay in months | NR | PCC | NS |
|  |  |  |  |  |  |  | Ferritin | NR | PCC | NS |
|  |  |  |  |  |  |  | Folic acid | NR | PCC | NS |
|  |  |  |  |  |  |  | Fructosamine | 0.25 | PCC | 0.001 |
|  |  |  |  |  |  |  | Functional ability | NR | PCC | NS |
|  |  |  |  |  |  |  | Glucose | NR | PCC | NS |
|  |  |  |  |  |  |  | Glutathionine reductase | NR | PCC | NS |
|  |  |  |  |  |  |  | Glycosylated haemoglobin | 0.04 | PCC | 0.04 |
|  |  |  |  |  |  |  | Haemoglobin | 0.04 | PCC | 0.04 |
|  |  |  |  |  |  |  | Hydroxyroline | NR | PCC | NS |
|  |  |  |  |  |  |  | Hypertension | NR | PCC | NS |
|  |  |  |  |  |  |  | Infection | NR | PCC | NS |
|  |  |  |  |  |  |  | Mid arm circumference (cm) | NR | PCC | NS |
|  |  |  |  |  |  |  | Musculoskeletal problem | NR | PCC | NS |
|  |  |  |  |  |  |  | Musculoskeletal problems | NR | PCC | NS |
|  |  |  |  |  |  |  | Neoplasm | NR | PCC | NS |
|  |  |  |  |  |  |  | Number of drugs taken | NR | PCC | NS |
|  |  |  |  |  |  |  | Parkinson's disease | NR | PCC | NS |
|  |  |  |  |  |  |  | Phosphate | NR | PCC | NS |
|  |  |  |  |  |  |  | Prealbumin | 0.03 | PCC | 0.05 |
|  |  |  |  |  |  |  | Red blood cell thiamine transketolase | NR | PCC | NS |
|  |  |  |  |  |  |  | Renal and liver function tests | NR | PCC | NS |
|  |  |  |  |  |  |  | Retinol | NR | PCC | NS |
|  |  |  |  |  |  |  | Retinol binding protein | NR | PCC | NS |
|  |  |  |  |  |  |  | Self-feeding | NR | PCC | NS |
|  |  |  |  |  |  |  | Systolic blood pressure (mm Hg) | NR | PCC | NS |
|  |  |  |  |  |  |  | Total protein | NR | PCC | NS |
|  |  |  |  |  |  |  | Total thyoxine (pmolfl) | NR | PCC | NS |
|  |  |  |  |  |  |  | Total white cell count | NR | PCC | NS |
|  |  |  |  |  |  |  | Transferrin | 0.07 | PCC | 0.01 |
|  |  |  |  |  |  |  | Triceps skin fold (mm) | NR | PCC | NS |
|  |  |  |  |  |  |  | Urate | NR | PCC | NS |
|  |  |  |  |  |  |  | Urinary electrolytes | NR | PCC | NS |
|  |  |  |  |  |  |  | Urinary hydroxyproline | NR | PCC | NS |
|  |  |  |  |  |  |  | Vitamin B12 | NR | PCC | NS |
|  |  |  |  |  |  |  | Vitamin D | NR | PCC | NS |
|  |  |  |  |  |  |  | Vitamin E | NR | PCC | NS |
|  |  |  |  |  |  |  | Vitamins or mineral supplements | NR | PCC | NS |
|  |  |  |  |  |  |  | Zinc | NR | PCC | NS |
| Zuiliani et al, 2001(49)  Italy  Longitudinal study  QAS: 8 | n=344 | Residents aged 66 years and over who have been residents for at least two months | 81.1 (7.2) survived 83.8 (7.0) deceased  79% female | Nursing home, n=1 | Baseline data collection 1990, follow up for two years. Data collection by geriatricians within the nursing home.  Analysis: Stepwise logistic regression | NR | % fat free | NR | OR (CI) | NS |
|  |  |  |  |  |  |  | % fat free mass | NR | OR (CI) | NS |
|  |  |  |  |  |  |  | ADLS: 0-1 lost ADLs (ref) vs 2-5 lost ADLs | 1.85 (0.90-3.95) | OR (CI) | 0.9 |
|  |  |  |  |  |  |  | ADLS: 0-1 lost ADLs (ref) vs 6 lost ADLs | 3.37 (1.56-7.30) | OR (CI) | 0.02 |
|  |  |  |  |  |  |  | Age | NR | OR (CI) | NS |
|  |  |  |  |  |  |  | Albumin | NR | OR (CI) | NS |
|  |  |  |  |  |  |  | Apolipoprotein B (apo B) | NR | OR (CI) | NS |
|  |  |  |  |  |  |  | Apolipoprotein A-I (apo A-I) | NR | OR (CI) | NS |
|  |  |  |  |  |  |  | Blood glucose | NR | OR (CI) | NS |
|  |  |  |  |  |  |  | Blood nitrogen | NR | OR (CI) | NS |
|  |  |  |  |  |  |  | Blood Pressure ≥4.35 g/dL (ref) vs <3.95 | 3.0 (1.65-5.43) | OR (CI) | 0.34 |
|  |  |  |  |  |  |  | Blood Pressure ≥4.35 g/dL (ref) vs 3.95-4.34 | 1.05 (0.53-2.07) | OR (CI) | 0.08 |
|  |  |  |  |  |  |  | Blood urea | NR | OR (CI) | NS |
|  |  |  |  |  |  |  | Body mass index | NR | OR (CI) | NS |
|  |  |  |  |  |  |  | Body reactance | NR | OR (CI) | NS |
|  |  |  |  |  |  |  | Body resistance | NR | OR (CI) | NS |
|  |  |  |  |  |  |  | Body water content | NR | OR (CI) | NS |
|  |  |  |  |  |  |  | Chloride (Cl) | NR | OR (CI) | NS |
|  |  |  |  |  |  |  | Comorbidity | NR | OR (CI) | NS |
|  |  |  |  |  |  |  | Current drug use | NR | OR (CI) | NS |
|  |  |  |  |  |  |  | Folic acid | NR | OR (CI) | NS |
|  |  |  |  |  |  |  | Gender | NR | OR (CI) | NS |
|  |  |  |  |  |  |  | Glycated haemoglobin (HbA1c) | NR | OR (CI) | NS |
|  |  |  |  |  |  |  | HDL-cholesterol | NR | OR (CI) | NS |
|  |  |  |  |  |  |  | Hematocrit | NR | OR (CI) | NS |
|  |  |  |  |  |  |  | Hemoglobin | NR | OR (CI) | NS |
|  |  |  |  |  |  |  | Low-density lipoprotein cholesterol (LDL) | NR | OR (CI) | NS |
|  |  |  |  |  |  |  | Potassium (K) | NR | OR (CI) | NS |
|  |  |  |  |  |  |  | Red blood cells | NR | OR (CI) | NS |
|  |  |  |  |  |  |  | Serum iron | NR | OR (CI) | NS |
|  |  |  |  |  |  |  | Sodium | NR | OR (CI) | NS |
|  |  |  |  |  |  |  | Subscapular skinfold thickness | NR | OR (CI) | NS |
|  |  |  |  |  |  |  | T3 | NR | OR (CI) | NS |
|  |  |  |  |  |  |  | T4 | NR | OR (CI) | NS |
|  |  |  |  |  |  |  | Thyroid-stimulating hormone (TSH) | NR | OR (CI) | NS |
|  |  |  |  |  |  |  | Total cholesterol | NR | OR (CI) | NS |
|  |  |  |  |  |  |  | Total protein | NR | OR (CI) | NS |
|  |  |  |  |  |  |  | Transferrin | NR | OR (CI) | NS |
|  |  |  |  |  |  |  | Tricipital skinfold thickness | NR | OR (CI) | NS |
|  |  |  |  |  |  |  | Triglycerides | NR | OR (CI) | NS |
|  |  |  |  |  |  |  | Vitamin B-12 | NR | OR (CI) | NS |
|  |  |  |  |  |  |  | Waist/hip ratio | NR | OR (CI) | NS |
|  |  |  |  |  |  |  | White blood cell count | NR | OR (CI) | NS |

Notes: Unless otherwise stated higher scores = poorer prognosis.

*QAS = Quality Assessment Score

** presented for reference only, not included in analysis.

Abbreviations:

AD - Alzheimer's disease, ADL -Activities of daily living, BCRS - Brief Cognitive Rating Scale , BEHAVE-AD - Behavioural Pathology in Alzheimer’s Disease Rating Scale, BMI - Body Mass Index, BOP - Behaviour Rating Scale for Elderly Patients, CAMA - corrected arm muscle area , CCI - Charlson Comorbidity Index, CDR/CDRS - Clinical Dementia Rating Scale, CI - Confidence Interval, CMAI - Cohen-Mansfield Agitation Inventory, COPD - chronic obstructive pulmonary disease, CSD - Cornell Scale for Depression , DAD-K - Disability Assessment for Dementia Scale - Korean , DRS - Depression Rating Scale, ECG - electrocardiogram, EQ-5D - EuroQol- 5 Dimension , GDS - Global Deterioration Scale , GMHR - General Medical Health Rating, HHSNRS - Hebrew Home Social Network Rating Scale, HR - Hazard Ratio, IMD - Index of multiple deprivation, IRR – Incidence Rate Ratio, ISE - Index for Social Engagement , MDS- Minimum Data Set, MDS Cog - Minimum Data Set Cognition Scale, MMSE - Mini Mental State Exam, MNA - Mini Nutritional Assessment, MRR – Mortality rate ratio, OR - Odds ratio, PAI - Patient assessment instrument, PCC – Partial Correlation Coefficient, PD - Parkinson's Disease, PGDRS - Psychogeriatric Dependency Rating Scale, RDRS-2 - Rapid Disability Rating Scale-2, RHR – Relative hazard rate, RR- Risk ratio, SCS- Self-Compassion Scale, SE - Standard Error, SPMSQ - Short portable mental status questionnaire, SPQ - Sleep Patterns Questionnaire, TIA - transient ischaemic attack, UTI - urinary tract infection.

References

1. Bebbington A, Brown P, Darton R, Miles K, Netten A. Survey of Admissions to Residential and Nursing Home Care. 18 Month Follow-Up. University of Kent at Canterbury: Personal Social Services Research Unit. 1998. Discussion Paper 1428/2.

2. Bebbington A, Darton R, Bartholomew R, Netten A. Survey of Admissions to Residential and Nursing Home Care Final report of the 42 month follow-up University of Kent at Canterbury: Personal Social Services Research Unit. 2000. Discussion Paper 1675.

3. Bebbington A, Darton R, Bartholomew R, Netten A, Brown P. Survey of Admissions to Residential and Nursing Home Care Final Report of the 30 Month Follow-Up. University of Kent at Canterbury: Personal Social Services Research Unit. 2000. Discussion Paper 1623.

4. Darton R, Brown P. Survey of Admissions to Residential Care Analyses of Six Month Follow-Up. University of Kent at Canterbury: Personal Social Services Research Unit. 1997. Discussion Paper 1340.

5. Breuer B, Wallenstein S, Feinberg C, Camargo MF, Libow LS. Assessing life expectancies of older nursing home residents. Journal of the American Geriatrics Society. 1998;46(8):954-61.

6. Carlson MC, Brandt J, Steele C, Baker A, Stern Y, Lyketsos CG. Predictor index of mortality in dementia patients upon entry into long-term care. The Journals of Gerontology. 2001;56(9):M567-70.

7. Cereda E, Pedrolli C, Zagami A, Vanotti A, Piffer S, Faliva M, et al. Alzheimer's disease and mortality in traditional long-term care facilities. Archives of Gerontology & Geriatrics. 2013;56(3):437-41.

8. Chan TC, Shea YF, Luk KH, Chan HW, Chu LW. Development and validation of a prognostic index for 2‐year mortality in Chinese older residents living in nursing homes. Geriatrics & Gerontology International. 2012;12(3):555-62.

9. Cohen Mansfield JC, Marx MS, Lipson S, Werner P. Predictors of mortality in nursing home residents. Journal of Clinical Epidemiology. 1999;52(4):273-80.

10. Connolly MJ, Broad JB, Boyd M, Kerse N, Gott M. Residential aged care: the de facto hospice for New Zealand's older people. Australasian journal on ageing. 2014;33(2):114-20.

11. Dale MC, Burns A, Panter L, Morris J. Factors affecting survival of elderly nursing home residents. International Journal of Geriatric Psychiatry. 2001;16(1):70-6.

12. Dontas AS, Tzonou A, Kasviki-Charvati P, Georgiades GL, Christakis G, Trichopoulos D. Survival in a residential home: an eleven-year longitudinal study. Journal of the American Geriatrics Society. 1991;39(7):641-9.

13. Fernandez HH, Lapane KL. Predictors of mortality among nursing home residents with a diagnosis of Parkinson's disease. Medical Science Monitor. 2002;8(4):CR241-CR6.

14. Flacker JM, Kiely DK. A practical approach to identifying mortality-related factors in established long-term care residents. Journal of the American Geriatrics Society. 1998;46(8):1012-5.

15. Flacker JM, Kiely DK. Mortality-related factors and 1-year survival in nursing home residents. Journal of the American Geriatrics Society. 2003;51(2):213-21.

16. Foebel AD, Heckman GA, Ji K, Dubin JA, Turpie ID, Hussack P, et al. Heart failure--related mortality and hospitalization in the year following admission to a long-term care facility: the geriatric outcomes and longitudinal decline in heart failure (GOLD-HF) study. Journal of Cardiac Failure. 2013;19(7):468-77.

17. Forder J, Fernandez J-L. Length of stay in care homes. Canterbury: Personal Social Services Research Unit; 2011. Discussion Paper 2769.

18. Formiga F, Riera-Mestre A, Chivite D, Pujol R, Ferrer A, Lopéz-Soto A. Predictors of 3-year mortality in institutionalized nonagenarians the NonaSantfeliu Study. Journal of the American Medical Directors Association. 2009;10(6):444-5.

19. Gambassi G, Landi F, Lapane KL, Sgadari A, Mor V, Bernabei R. Predictors of mortality in patients with Alzheimer's disease living in nursing homes. Journal of Neurology, Neurosurgery & Psychiatry. 1999;67(1):59-65.

20. Hedinger D, Hammig O, Bopp M, Swiss National Cohort Study G. Social determinants of duration of last nursing home stay at the end of life in Switzerland: a retrospective cohort study. BMC Geriatrics. 2015;15:114.

21. Heppenstall CP, Broad JB, Boyd M, Gott M, Connolly MJ. Progress towards predicting 1-year mortality in older people living in residential long-term care. Age & Ageing. 2015;44(3):497-501.

22. Hjaltadottir I, Hallberg IR, Ekwall AK, Nyberg P. Predicting mortality of residents at admission to nursing home: a longitudinal cohort study. BMC health services research. 2011;11:86.

23. Hui E, Wong EMC, Woo J. Assessing life expectancies of institutionalized older persons in Hong Kong. Gerontology. 2004;50(3):165-70.

24. Lapane KL, Gambassi G, Landi F, Sgadari A, Mor V, Bernabei R. Gender differences in predictors of mortality in nursing home residents with AD. Neurology. 2001;56(5):650-4.

25. Lichtenstein MJ, Federspiel CF, Schaffner W. Factors associated with early demise in nursing home residents: a case control study. Journal of the American Geriatrics Society. 1985;33(5):315-9.

26. Lucchetti G, Lucchetti ALG, Pires SL, Gorzoni ML. Predictors of death among nursing home patients: A 5‐year prospective study. Geriatrics & Gerontology International. 2015;15(2):234-6.

27. Luk JKH, Chan WK, Ng WC, Chiu PKC, Ho C, Chan TC, et al. Mortality and health services utilisation among older people with advanced cognitive impairment living in residential care homes. Hong Kong Medical Journal. 2013;19(6):518-24.

28. McCann M, O'Reilly D, Cardwell C. A Census-based longitudinal study of variations in survival amongst residents of nursing and residential homes in Northern Ireland. Age & Ageing. 2009;38(6):711-7.

29. Mehr DR, Williams BC, Fries BE. Predicting discharge outcomes of VA nursing home residents. Journal of Aging and Health. 1997;9(2):244-65.

30. Mitchell S, Kiely D, Hamel M, Park P, Morris J, Fries B. Estimating prognosis for nursing home residents with advanced dementia. Journal of the American Medical Association. 2004;291(22):2734-40.

31. Mitchell SL, Miller SC, Teno JM, Davis RB, Shaffer ML. The advanced dementia prognostic tool: a risk score to estimate survival in nursing home residents with advanced dementia. Journal of Pain & Symptom Management. 2010;40(5):639-51.

32. Navarro-Gil P, González-Vélez AE, Ayala A, Martín-García S, Martínez-Martín P, Forjaz MJ. Which factors are associated with mortality in institutionalized older adults with dementia? Archives of Gerontology & Geriatrics. 2014;59(3):522-7.

33. Netten A, Bebbington A, Picton-Williams T, Cruttenden L. Length of stay and mortality of nursing home residents. Health & Social Care in the Community. 1995;3(6):383-8.

34. Nygaard HA, Laake K. Lower mortality of demented nursing home residents. A two-year survival study. Scandinavian Journal of Primary Health Care. 1990;8(2):123-6.

35. Porock D, Oliver DP, Zweig S, Rantz M, Mehr D, Madsen R, et al. Predicting death in the nursing home: development and validation of the 6-month Minimum Data Set Mortality Risk Index. Journals of Gerontology Series A: Biological Sciences & Medical Sciences. 2005;60(4):491-8.

36. Rothera IC, Jones R, Harwood R, Avery AJ, Waite J. Survival in a cohort of social services placements in nursing and residential homes: factors associated with life expectancy and mortality. Public Health (Nature). 2002;116(3):160-5.

37. Shah SM, Carey IM, Harris T, DeWilde S, Cook DG. Mortality in older care home residents in England and Wales. Age & Ageing. 2013;42(2):209-15.

38. Sharifi F, Ghaderpanahi M, Fakhrzadeh H, Mirarefin M, Badamchizadeh Z, Tajalizadekhoob Y, et al. Older people's mortality index: Development of a practical model for prediction of mortality in nursing homes. Geriatrics & Gerontology International. 2012;12(1):36-45.

39. Sokejima S, Naruse Y, Yamagami T, Kagamimori S. Recent changes in stroke history, mobility status and life expectancy at admission among nursing home residents in Japan. Health & Social Care in the Community. 1996;4(2):96-102.

40. Spector WD, Takada HA. Characteristics of nursing homes that affect resident outcomes. Journal of Aging & Health. 1991;3(4):427-54.

41. Suh G, Yeon BK, Shah A, Lee J. Mortality in Alzheimer's disease: a comparative prospective Korean study in the community and nursing homes. International Journal of Geriatric Psychiatry. 2005;20(1):26-34.

42. Sund Levander M, Milberg A, Rodhe N, Tingstrom P, Grodzinsky E. Differences in predictors of 5-year survival over a 10-year period in two cohorts of elderly nursing home residents in Sweden. Scandinavian Journal of Caring Sciences. 2016; 30(4):714-720.

43. Sung K. Predictive factors associated with death of elderly in nursing homes. Asian Nursing Research. 2014;8(2):143-9.

44. Troyer JL. Examining differences in death rates for Medicaid and non-Medicaid nursing home residents. Medical Care. 2004;42(10):985-91.

45. van Dijk PTM, Mehr DR, Ooms ME, Madsen R, Petroski G, Frijters DH, et al. Comorbidity and 1-year mortality risks in nursing home residents. Journal of the American Geriatrics Society. 2005;53(4):660-5.

46. Van Dijk PTM, Dippel DWJ, Van Der Meulen JHP, Habbema JDF. Comorbidity and its effect on mortality in nursing home patients with dementia. Journal of Nervous and Mental Disease. 1996;184(3):180-7.

47. Wallace JB, Prevost SS. Two methods for predicting limited life expectancy in nursing homes. Journal of Nursing Scholarship. 2006;38(2):148-53.

48. Woo J, Chan SM, Mak YT, Swaminathan R. Biochemical predictors of short term mortality in elderly residents of chronic care institutions. Journal of Clinical Pathology. 1989;42(12):1241.

49. Zuliani G, Romagnoni F, Soattin L, Leoci V, Volpato S, Fellin R. Predictors of two-year mortality in older nursing home residents. The IRA study. Aging Clinical and Experimental Research. 2001;13(1):3-7.
